# Towards Development of Small Molecule Lipid II Inhibitors as Novel Antibiotics

**DOI:** 10.1371/journal.pone.0164515

**Published:** 2016-10-24

**Authors:** Jamal Chauhan, Steven Cardinale, Lei Fang, Jing Huang, Steven M. Kwasny, M. Ross Pennington, Kelly Basi, Robert diTargiani, Benedict R. Capacio, Alexander D. MacKerell, Timothy J. Opperman, Steven Fletcher, Erik P. H. de Leeuw

**Affiliations:** 1 Department of Pharmaceutical Sciences, University of Maryland, School of Pharmacy, Baltimore, Maryland, United States of America; 2 Computer-Aided Drug Design Center, University of Maryland, School of Pharmacy, Baltimore, Maryland, United States of America; 3 Center for Biomolecular Therapeutics, University of Maryland School of Medicine, Baltimore, Maryland, United States of America; 4 Microbiotix, Inc., One Innovation Drive, Worcester, Massachusetts, United States of America; 5 U.S. Army Medical Research Institute of Chemical Defense, Aberdeen Proving Ground, Maryland, United States of America; 6 Institute of Human Virology & Department of Biochemistry and Molecular Biology of the University of Maryland Baltimore School of Medicine, Baltimore, Maryland, United States of America; University of Cambridge, UNITED KINGDOM

## Abstract

Recently we described a novel di-benzene-pyrylium-indolene (BAS00127538) inhibitor of Lipid II. BAS00127538 (*1-Methyl-2*,*4-diphenyl-6-((1E*,*3E)-3-(1*,*3*,*3-trimethylindolin-2-ylidene)prop-1-en-1-yl)pyryl-1-ium) tetrafluoroborate* is the first small molecule Lipid II inhibitor and is structurally distinct from natural agents that bind Lipid II, such as vancomycin. Here, we describe the synthesis and biological evaluation of 50 new analogs of BAS00127538 designed to explore the structure-activity relationships of the scaffold. The results of this study indicate an activity map of the scaffold, identifying regions that are critical to cytotoxicity, Lipid II binding and range of anti-bacterial action. One compound, 6jc48-1, showed significantly enhanced drug-like properties compared to BAS00127538. 6jc48-1 has reduced cytotoxicity, while retaining specific Lipid II binding and activity against *Enterococcus spp*. *in vitro* and *in vivo*. Further, this compound showed a markedly improved pharmacokinetic profile with a half-life of over 13 hours upon intravenous and oral administration and was stable in plasma. These results suggest that scaffolds like that of 6jc48-1 can be developed into small molecule antibiotic drugs that target Lipid II.

## Introduction

The biosynthesis pathway of the bacterial cell wall is well studied and a validated target for the development of antibacterial agents. Cell wall biosynthesis involves two major processes; 1) the biosynthesis of cell wall teichoic acids and 2) the biosynthesis of peptidoglycan. Key molecules in these pathways, including enzymes and precursor molecules are attractive targets for the development of novel antibacterial agents [1–4]. The cell wall of both Gram-negative and Gram-positive bacteria comprises a peptidoglycan layer which is composed of a polymer of alternating amino sugars, N-acetylglucosamine (GlcNAc) and N-acetylmuramic acid (MurNAc). On the cytoplasmic side of the plasma membrane, the soluble precursor UDP-MurNAc-pentapeptide is linked to the membrane carrier bactoprenol- phosphate (C_55_P) yielding Lipid I. In a second step GlcNac is added by the enzyme MurG to yield Lipid II [1]. Lipid II is essential for cell wall biosynthesis, is synthesized in limited amounts and has a high turnover rate, making it an attractive and established target for antibacterial compounds.

Various classes of natural antibiotic peptides have been discovered that bind Lipid II, including depsipeptides, lantibiotics, cyclic peptides and glycopeptides [1]. Of these, vancomycin and its more recently developed derivatives daptomycin, oritavancin and telavancin are approved as first line treatments for Gram-positive infections [5–8]. However, resistance to these drugs is increasingly reported [9–11]. Several studies on defensins, effector peptides of innate immunity [12], revealed specific interactions with Lipid II, adding another class of natural compounds to the growing list of structurally unrelated peptides that bind this target [13–19]. Based on the interaction between Lipid II and Human Neutrophil Peptide -1, we previously identified, for the first time, low molecular weight synthetic compounds that target Lipid II with high specificity and affinity [20]. One of our lead compounds, BAS00127538, was characterized further and revealed a unique interaction with Lipid II that differs from antibiotics currently in clinical use or development [20]. In this study, we report on the structural and functional relationships of derivatives of BAS00127538.

## Materials and Methods

### Materials and Bacterial Strains

*Staphylococcus aureus* ATCC 29213, *Escherichia coli* ATCC 25922, *Enterococcus faecalis* ATCC 29212, *Streptococcus pneumonia* ATCC 49619 and *Acinetobacter baumanii* ATCC 19606 were obtained from Microbiologics (St. Cloud, MN). *E*. *faecalis* ATCC 51575, ATCC 51299 and REMEL C99707 and *E*. *faecium* ATCC 51559 (MDR), REMEL IH79985 and REMEL C110914 were generously provided by the Laboratory of Pathology, University of Maryland Baltimore School of Medicine. Unless stated otherwise, chemicals and reagents were purchased from Sigma.

### CADD modeling and MD simulations

Molecular modeling, energy minimization and Molecular Dynamics (MD) simulations were performed with the program CHARMM [21] using the CHARMM36 lipid [22] protein [23, 24] and carbohydrate [25, 26] force field for Lipid II, the TIP3P water model [27] along with the CHARMM General force field [28–30] for the ligands. Using the final snapshot from the previously published 10 ns MD simulations of the BAS00127538-Lipid II complex in aqueous solution the aromatic rings of the 48–1 analogs were aligned with those of BAS00127538. The system was then subjected to a short energy minimization following which a 100 ps MD simulation with an integration time step of 0.5 fs was carried out. The system was then subjected to a 20 ns MD simulation run with a time step of 1 fs. Simulations were carried out in the NPT ensemble at 300 K and 1 atm with SHAKE of covalent bonds involving hydrogens, and there were no restraints in the simulations. The final structures from the simulations were used for visualization of the ligand-Lipid II interactions.

### 3-Lipid II purification

Short-chain water-soluble Lipid II containing a lipid tail of three isoprene units was generated and purified essentially as described [31]. Typically, *M*. *flavus* vesicles (120 μmol lipid-Pi) were incubated together with 500 μmol UDP-GlcNAc, 500 μmol UDP-MurNAC-pentapeptide and 400 μmol farnesyl phosphate in 100 mM Tris-HCl pH 8.0, 5 mM MgCl_2_. The incubation lasted two hours at room temperature for 3-P. The synthesis of 3-Lipid II was followed using RP-8 reversed phase TLC (Merck) developed in 75% methanol. For purification, the membranes were removed by centrifugation at 40,000 x g and the supernatant was collected and loaded on a C18 HPLC column and eluted with a linear gradient from 50 mM ammonium bicarbonate to 100% methanol in 30 minutes. Farnesyl-Lipid II (3-Lipid II) eluted at approximately 60% methanol. Its identity was confirmed by mass spectroscopy.

### Surface Plasmon Resonance

Surface Plasmon Resonance binding experiments were carried out on a BIAcore T100 system (BIAcore Inc., Piscataway, NY) at 25°C. The assay buffer was 10 mM HEPES, 150 mM NaCl, 0.05% surfactant P20, pH 7.4 (± 3 mM EDTA) supplemented with 10% DMSO. 3-Lipid II (50 RUs) was immobilized on CM5 sensor chips using the amine-coupling chemistry recommended by the manufacturer. For initial determination of binding, compounds were introduced into the flow-cells (30 μl/min) in the running buffer at 10 μM. Resonance signals were corrected for nonspecific binding by subtracting the background of the control flow-cell. After each analysis, the sensor chip surfaces were regenerated with 50 mM NaOH for 30 s at a flow rate 100 μl/min, and equilibrated with the buffer prior to next injection. For binding kinetics studies, binding isotherms were analyzed with manufacturer-supplied software for BIAcore T100.

### Antibacterial activity assay

Determination of the Minimal Inhibitory Concentrations (MIC) by dilution was carried out by broth dilution according to CLSI standards [32].

### Cytotoxicity

The cytotoxicity concentration of antibacterial compounds that produces half maximal decrease in viability (CC50) against mammalian cells (HeLa, ATCC CCL-2.2) was determined as described [33]. The effect of compounds on HeLa cell viability was assessed in triplicate by measuring the mitochondrial activity using MTS assays according to the manufacturer’s instructions (Cell Titer 96 proliferation assay, Promega). The cells were incubated for 72 hours in RPMI1640 medium containing the compounds at final concentrations ranging from 64 to 0.125 μg/ml. CC50 was determined using a standard curve of serially diluted untreated cells in each experiment.

### Macromolecular synthesis assays

The effect of compounds on the macromolecular synthetic pathways of *E*. *faecalis* EF1509 were measured as follows: Cells were grown at 35°C overnight on Tryptic Soy Agar Broth (Remel, Lenexa, KS), and growth from the plate was used to inoculate 15 ml of Mueller Hinton Broth. The culture was grown to early exponential growth phase (OD_600_ = 0.2 to 0.3) while incubating in a shaker at 35°C and 150 rpm. For each macromolecular assay, the test agents were added at either 0, 0.25, 0.5, 1, 2, or 4, -fold their respective MIC values for *E*. *faecalis* EF1509. As positive control drugs, the following antibiotics were added at 8X MIC in order to validate each assay: Vancomycin (cell wall synthesis); ciprofloxacin (DNA synthesis), rifampin (RNA synthesis), cerulenin (lipid synthesis), and linezolid (protein synthesis).

For DNA and protein synthesis, 100 μl of cell culture reaching early exponential phase was added to triplicate wells containing various concentrations of test compound or control antibiotics (2.5 μl) at 40X the final concentration in 100% DMSO (0.1% methanol in water for Rifampicin). A 2.5% DMSO treated culture served as the “no drug” control for all experiments. Cells were added in 1.25X strength MHB to account for the volume of drug added to each reaction, or in M9 minimal medium for protein synthesis reactions. Following a 5 min incubation at room temperature either [^3^H]Thymidine (DNA synthesis) or [^3^H]Leucine (protein synthesis) was added at 0.5–1.0 μCi per reaction, depending on the experiment. Reactions were allowed to proceed at room temperature for 15–40 min and then stopped by adding 12 μl of cold 5% trichloroacetic acid (TCA) or 5% TCA/2% casamino acids (protein synthesis). Reactions were incubated on ice for 30 min and the TCA precipitated material was collected on a 25 mm GF/1.2 μm PES 96 well filter plate (Corning). After washing five times with 200 μl per well of cold 5% TCA, the filters were allowed to dry, and then counted using a Packard Top Count microplate scintillation counter.

For cell wall synthesis, bacterial cells in early exponential growth phase were transferred to M9 minimal medium and added to 1.5 ml eppendorf tubes (100 μl/tube) containing various concentrations of test compound or control antibiotics (2.5 μl) at 40X the final concentration in 100% DMSO as described above. Following a 5 min incubation at 37°C, [^14^C] N-acetyl-glucosamine (0.4 μCi/reaction) was added to each tube and incubated for 45 min in a 37°C heating block. Reactions were stopped through the addition of 100 μl of 8% SDS to each tube. Reactions were then heated at 95°C for 30 min in a heating block, cooled, briefly centrifuged, and spotted onto pre-wet HA filters (0.45 μM). After washing three times with 5 ml of 0.1% SDS, the filters were rinsed two times with 5 ml of deionized water, allowed to dry, and then counted using a Beckman LS3801 liquid scintillation counter.

For lipid synthesis, bacterial cells were grown to early exponential growth phase in MHB and 100 μl was added to 1.5 ml Eppendorf tubes (in triplicate) containing various concentrations of test compound or control antibiotics as described above. Following a 5 min incubation at room temp., [^3^H] glycerol was added at 0.5 μCi per reaction. Reactions were allowed to proceed at room temperature for 40 min and then stopped through the addition of 375 μl of chloroform/methanol (1:2) followed by vortexing for 20 sec after. Chloroform (125 μl) was then added to each reaction and vortexed, followed by the addition of 125 μl dH_2_O and vortexing. Reactions were centrifuged at 13,000 rpm for 10 min, and then 150 μl of the organic phase was transferred to a scintillation vial and allowed to dry in a fume hood for at least 1 hr. Samples were then counted via liquid scintillation counting. Each data point is the average of three replicates and the error bars represent standard deviation.

### Chemical synthesis

The general procedure for pyrylium salt synthesis is given in Scheme 1.

#### Scheme 1

general procedure for pyrylium salt synthesis. To a substituted acetophenone and acetic anhydride was added boron trifluoride etherate (32.0 mmol) at room temperature. The reaction was heated to 135°C for 4 h, cooled, poured into EtOAc and allowed to stand for 1 h. The yellow solid was filtered and washed with excess EtOAc to give the title compounds as the boron tetrafluoride salts.

The general procedure for the condensation reaction with aldehydes is given in Scheme 2.

#### Scheme 2

General procedure for condensation with aldehydes. Pyrylium salt (0.28 mmol) and aldehyde (0.34 mmol) in MeOH (8 mL) was heated to reflux for 4 h. The reaction was cooled, reduced in vacuo, poured into EtOAc and allowed to stand for 1 h. The dark solid was filtered and washed with excess EtOAc to give the title compounds as the boron tetrafluoride salt.

#### Scheme 3

General synthesis of the BAS00127538 scaffold and variation at the R1 and R2 positions.

Detailed chemical synthesis and characterization of compounds described in this study is listed in the experimental supplemental section.

### *In vitro* ADMET studies

*Liquid Chromatography Tandem Mass Spectrometry Analysis*: For liquid chromatography tandem mass spectrometry (LC-MS/MS) analysis, a Sciex 6500 QTrap Triple Quadrupole Mass Spectrometer (Sciex, Ottawa, Ontario) coupled with an Agilent 1290 Infinity Liquid Chromatograph (Agilent Technologies, Santa Clara, CA) was employed. Separation was performed on a Halo C18 Column (2.7um, 2.1mm x 50mm) (Advanced Materials Technology, Wilmington, DE) with mobile phase A being methanol with 0.1% formic acid and mobile phase B being 0.1% formic acid in water. A chromatographic ramp was employed consisting of 0 min➔ 3 min: 95% mobile phase B ➔ 95% mobile phase A, 3 min ➔ 3.1 min: 95% mobile phase A ➔ 95% mobile phase B, 3.1 min ➔ 6min: 95% mobile phase B. The chromatographic flow rate was 500ul/min. The autosampler compartment was held at 10°C. The mass spectrometer was operated in positive, electrospray mode using multiple reaction monitoring (MRM). The following MS setting were employed: ion source temperature, 600°C; capillary voltage, +5500V; curtain gas, 30; collision assisted dissociation (CAD) gas, medium; ion source gas 1 and 2, were 50 and 70 respectively; declustering potential, 45V; entrance potential, 10V. The ion transitions: 525.9Da ➔ 182.7Da, collision energy = 54eV, collision cell exit potential = 24V and 525.9Da➔ 155.0Da, collision energy = 94eV, collision cell exit potential = 19V were monitored. Peak areas were integrated using Analyst software (Sciex, Ottawa, Ontario).

#### Quality Control

Purity was assessesd was performed on a Sciex 6500 QTrap LC-MS/MS operated in Information Dependent Analysis (IDA) mode. The test compound was prepared at 5 uM and 500 nM in three matrices: 50/50 water/methanol, 50/50 water/methanol + 0.1% formic acid and 50/50 water/methanol + 10 mM ammonium bicarbonate. The IDA mass spectrometric method was designed to perform a full scan (20-700Da) and obtain a product ion spectrum (MS/MS) from each of the 3 most abundant ions in the full scan. This IDA was run in both positive and negative mode. When operated in negative mode a mobile phase of 50/50 water/methanol + 10mM ammonium bicarbonate was used. When operated in positive mode a mobile phase of 50/50 water/methanol + 0.1% formic acid was used. No LC column was employed, but an Agilent 1290 Infinity Liquid Chromatograph (Agilent Technologies, Santa Clara, CA) was used to produce an isocratic flow for introduction of samples directly into the mass spectrometer. Each sample was directly injected into the mass spectrometer, via the autosampler and the total run time for each sample was 2min. Peak areas were analyzed using the Analyst software. MS/MS data was compared to full spectrum data to determine if the most abundant peaks were due to the test compound, impurity, or in-source fragmentation of the test compound. A percent purity was calculated from the ratio of the known peak areas to the total peak areas in each positive and negative mode. Calculated percent purities, in positive and negative modes, were weighted according to the total observed signal in the full scans and averaged.

#### Plasma Stability

Plasma stability of compound 6jc48-1 was determined using heparinized, pooled human plasma (BioreclamationIVT, Hicksville, NY). Test compound was spiked into plasma at a final concentration of 1uM. Test compound solution was subsequently incubated at 37°C for up to 1 hour. Aliquots were removed at 0, 5, 10, 20, 30, 40, 50 and 60 min incubation time and diluted 1:2 in cold acetonitrile. Samples were centrifuged at 4000rpm for 10min. Supernatant was collected and diluted 1:2 in 30% methanol in water. The diluted supernatant was analyzed by LC-MS/MS using the method described above. Stability in plasma was calculated by integrating peak areas of samples using Analyst software (Sciex, Ottawa, Ontario).

#### Solubility

Solubility in water was determined using a NEPHELOstar^plus^ laser nephelometer (BMG Labtech, Cary, NC) at a wavelength of 635 nm and bottom read optics using a 96-well plate format. A solution of 6jc48-1 was prepared at a concentration of 2.5 mg/ml in DMSO. The DMSO solution (10 μl) was added to wells containing water (290 μl) for a final concentration of 125 μg/ml. The plate was incubated at room temperature for two hours prior to reading in the nephalometer. All samples were run in triplicate. Control samples (DMSO with no analyte) were prepared and run in parallel.

#### Cytochrome P450 inhibition

Cytochrome P450 inhibition was conducted according to the method of Paradise et al (2007) with modifications. Briefly, drug inhibition of the test compound was measured on specific cytochrome P450 enzymes using traditional substrates for CYP3A4, CYP2D6 and CYP2C19. Recombinant human CYP450 3A4, 2D6 and 2C19 enzymes (Supersome™) were obtained from Corning®. Supersomes™ typically have a CYP450 content of 1000–2000 pmol/ml. Standard substrates (mephenytoin, dextromethorphan, testosterone) were prepared at 500 μM in acetonitrile. The final concentration of each substrate was 1 μM. Positive control inhibitors and test compound were prepared 50X the final concentration in acetonitrile; 0.25 mM ketoconazole (inhibitor of 3A4), 25 uM quinidine (inhibitor of 2D6) and 5 mM tranylcypromine (inhibitor of 2C19). The typical IC_50_ values for the standard inhibitor/substrate combinations are listed in Table 1. Eight concentrations of a positive control inhibitor, eight concentrations of test compound, a no inhibitor control and a background control were tested. The test compound had a final concentration range from 20 μM to single–digit nanomolar. After a 10 minute pre-incubation at 37°C, a 2X concentrated enzyme/substrate mixture was added to all samples with the exception of the background control. The enzyme/substrate solution contained 100 mM potassium phosphate buffer (pH 7.4), water, substrate and 50 pmol/ml of the respective enzyme. The reactions were quenched with acetonitrile at the appropriate time points.

**Table 1:** Antibacterial activity, cytotoxicity and Lipid II binding of BAS00127538 derivatives.

	**6jc37**	**6jc38**	**6jc39**	**6jc41-1**	**6jc43-1**	**6jc43-2**	**6jc48-1**	**6jc48-2**	**Jc-49-1**	**6jc51-1**	**BAS-00127538**
*S*. *aureus* MRSA 1094	16	64	2	16	4	>=64	32	>=64	8	1	0.5
*S*. *aureus* HFH-30123 (MRSA)	16	>=64	4	32	4	>=64	32	>=64	8	2	0.5
*E*. *faecium* EF1509 (VRE)	64	>=64	4	22.62742	4	64	2.828427	4	16	0.5	2
*E*. *faecium* F118 (VRE)	64	>=64	4	8	4	>=64	5.656854	16	16	2	2
*K*. *pneumoniae* NR-15410 (KPC)	>=64	>=64	64	>=64	>=64	>=64	>=64	>=64	>=64	16	8
*K*. *pneumoniae* NR-15411 (KPC)	>=64	>=64	>=64	>=64	>=64	>=64	>=64	>=64	>=64	64	16
*A*. *baumanii* ATCC 19606	>=64	>=64	16	>=64	45.25483	>=64	>=64	>=64	>=64	8	4
*P*. *aeruginosa* PA01	>=64	>=64	>=64	>=64	>=64	>=64	>=64	>=64	>=64	64	64
*P*. *aeruginosa* X13273	>=64	>=64	>=64	>=64	>=64	>=64	>=64	>=64	>=64	45.25483	>=64
*P*. *aeruginosa* ATCC 27853	>=64	>=64	>=64	>=64	>=64	>=64	>=64	>=64	>=64	64	64
*E*. *cloacae* ATCC 13047	>=64	>=64	>=64	>=64	>=64	>=64	>=64	>=64	>=64	64	32
*E*. *aerogenes* ATCC 13048	>=64	>=64	>=64	>=64	>=64	>=64	>=64	>=64	>=64	64	16
CC_50%_ (72 h HeLa cells)	18.96	>32	1.31	>32	0.93	>32	>100	60.51	2.2	<.78125	0.56
CC_50%/MIC_ (based on S. aureus)	1.185	NA	0.655	>2	0.2325	NA	3.125	<0.945	0.275	<.78125	1.12
Lipid II binding Kd, µM	No	No	39±4	34±4	62±6	ND	0.15±0.03	1.14±0.3	0.17±0.05	9.2±2	1.81±0.3
	**6jc51-2**	**6jc-53-2**	**6jc-58**	**6jc-59-1**	**6jc-59-3**	**6jc-60-1**	**6jc64-1**	**6jc64-2**	**6jc64-3**	**6jc65-1**	**BAS-00127538**
*S*. *aureus* MRSA 1094	2	2	1.41421	2	4	8	4	8	5.66	2.83	0.5
*S*. *aureus* HFH-30123 (MRSA)	4	4	2	2	4	8	4	8	4	2	0.5
*E*. *faecium* EF1509 (VRE)	0.5	8	4	4	5.656854	8	4	5.66	8	2	2
*E*. *faecium* F118 (VRE)	2	5.65685	4	4	8	8	5.66	16	8	4	2
*K*. *pneumoniae* NR-15410 (KPC)	64	>=64	45.2548	>=64	>=64	>=64	>=64	>=64	64	64	8
*K*. *pneumoniae* NR-15411 (KPC)	>=64	>=64	64	>=64	>=64	>=64	>=64	>=64	64	>=64	16
*A*. *baumanii* ATCC 19606	32	>=64	8	>=64	>=64	>=64	16	>=64	>=64	11.31	4
*P*. *aeruginosa* PA01	>=64	>=64	64	>=64	>=64	>=64	>=64	>=64	>=64	64	64
*P*. *aeruginosa* X13273	>=64	>=64	32	>=64	>=64	>=64	>=64	>=64	>=64	64	>=64
*P*. *aeruginosa* ATCC 27853	>=64	>=64	64	>=64	>=64	>=64	>=64	>=64	>=64	64	>=64
*E*. *cloacae* ATCC 13047	>=64	>=64	64	>=64	>=64	>=64	>=64	>=64	64	64	32
*E*. *aerogenes* ATCC 13048	>=64	>=64	32	>=64	>=64	>=64	>=64	>=64	>=64	32	16
CC_50%_ (72 h HeLa cells)	<.78125	1.12	<.78125	<.78125	1.35	<.78125	<.78125	<.78125	3.29	<.78125	0.56
CC_50%/MIC_ (based on S. aureus)	<.39	0.56	0.56	0.39	0.3375	0.097	0.195	0.097	0.581	0.276	1.12
Lipid II binding Kd, µM	9.5±2	10±3	17±4	1.9±0.3	27.9±3	37.9±4	07.28±0.3	23.8±4	4.92±0.5	2.17±0.2	1.81±0.3
	**6jc65-2**	**6jc66-1**	**6jc66-2**	**6jc66-3**	**6jc66-4**	**6jc-67A**	**6jc-69-1**	**6jc-69-3**	**6jc-69-4**	**6jc-69-5**	**BAS-00127538**
*S*. *aureus* MRSA 1094	2	2.83	4	8	2	1	2	4	8	>=64	0.5
*S*. *aureus* HFH-30123 (MRSA)	2	4	4	8	2	1	2	4	8	64	0.5
*E*. *faecium* EF1509 (VRE)	2	4	4	4	2	2	2	2.83	4	6	2
*E*. *faecium* F118 (VRE)	4	8	5.66	11.31	4	1.41	2	4	5.66	11.31	2
*K*. *pneumoniae* NR-15410 (KPC)	64	>=64	64	>=64	64	16	>=64	>=64	>=64	>=64	8
*K*. *pneumoniae* NR-15411 (KPC)	>=64	>=64	>=64	>=64	>=64	32	>=64	>=64	>=64	>=64	16
*A*. *baumanii* ATCC 19606	32	45.25	64	>=64	64	4	32	>=64	>=64	>=64	4
*P*. *aeruginosa* PA01	>=64	>=64	>=64	>=64	>=64	64	>=64	>=64	>=64	>=64	64
*P*. *aeruginosa* X13273	>=64	>=64	>=64	>=64	>=64	32	>=64	>=64	>=64	>=64	>=64
*P*. *aeruginosa* ATCC 27853	>=64	>=64	>=64	>=64	>=64	32	>=64	>=64	>=64	>=64	>=64
*E*. *cloacae* ATCC 13047	64	>=64	>=64	>=64	>=64	32	>=64	>=64	>=64	>=64	32
*E*. *aerogenes* ATCC 13048	64	>=64	>=64	>=64	>=64	16	>=64	>=64	>=64	>=64	16
CC_50%_ (72 h HeLa cells)	0.94	1.36	1.42	5.68	0.92	<.78125	<.78125	<.78125	3.09	4.43	0.56
CC_50%/MIC_ (based on S. aureus)	0.47	0.48	0.355	0.71	0.46	0.78	0.39	0.195	0.38	0.06	1.12
Lipid II binding Kd, µM	3.9±0.4	2.9±0.3	1.6±0.2	60±11	27.9±2	7.89±0.2	16.1±0.3	0.6±0.1	30.3±0.5	32.7±2	1.81±0.3

All samples were centrifuged for 3 minutes at 13,000 x g at room temperature. The supernatant was collected for analysis by LC/MS/MS. Computation of IC_50_ values:The background of no-enzyme samples to determine the background value were averaged. The positive control or full-reaction samples to determine the signal value were averaged.

The percent activity of each sample was calculated as follows:

[(Test compound metabolite–average background)/(average signal–average background)] X 100 = % activity

GraphPad Prism software was used to plot the calculated percent activity values versus the log concentrations of test compound. The the IC50 value using non-linear regression was calculated.

IC50 values for standard inhibitors, calculated in-house, are shown in Table 2. Although IC_50_ ‘s may vary slightly, literature reports demonstrate similar values [34].

**Table 2:** Activity of 6jc48-1 against *Enterococcus spp*.

Organism:	MMX#-ATCC#	6jc48-1	Vancomycin
***E*. *faecium* REMEL**	**IH79985**	8	ND
***E*. *faecium* REMEL**	**C110914**	4	ND
***E*. *faecium***	**S1559**	2	ND
***E*. *faecalis***	**51575**	2	ND
***E*. *faecalis***	**51299**	4	ND
***E*. *faecalis* REMEL**	**C99707**	2	ND
*E*. faecium (n=5)*	**clinical isolates**	4 to 16	>32
*E*. faecalis (n=5)*	**clinical isolates**	4 to 16	>32

ND-Not Determined

* clinical isolates sensitive to linezolid and daptomycin

#### Liver Microsome Stability

The *in vitro* microsome stability assay was performed using human liver microsomes and a Biomek FXP liquid handling workstation to deliver reagents to a 96 deep well plate on a shaking peltier with temperature controls. Human liver microsomes were purchased from Corning®. The test compound was incubated in an aqueous reaction mixture (200 ul total volume) consisting of human liver microsomes (150 mixed donor pool) and NADPH Regenerating System Solutions A and B (Corning®) in the presence of 100 mM potassium phosphate buffer (pH 7.4). The NADPH solution A comprises nicotinamide adenine dinucleotide phosphate (NADP+) and glucose 6-phosphate and solution B contains glucose-6-phosphate dehydrogenase. Solutions A and B were combined prior to adding to the reaction plate to generate a supply of NADPH. The NADPH was added last to simultaneously initiate the reactions. The final concentration of the test compound was 10 uM and the microsomal protein concentration was 0.5 mg/ml. After incubation at 37°C, the reaction was terminated at 0, 5, 10, 20, 30, 40, 50 and 60 minutes, respectively, by the addition of 600 uL of acetonitrile. Three replicates were run for each time point. The quenched reaction plate was centrifuged at 4500 rpm for 10 minutes. The supernatant was diluted 1:2 in 30% Methanol in water for LC/MS/MS analyses to monitor substrate depletion. Water was substituted for NADPH for the zero time-point samples. A control plate, without NADPH cofactor, was completed on the same day, using the same conditions.

The half-life (t1/2) was obtained using the equation below:

$$t_{1/2}=\frac{-0.693}{\mathrm{slope}}$$

Where slope is the slope of the line formed by ln(% remaining test compound) vs. time. The in vitro intrinsic clearance (CLint) value was calculated using equation:

$${CL}_{\mathrm{int}}=\left(\frac{0.693}{t_{1/2}}\right)\left(\frac{\mathrm{incubation volume}\;(ul)}{\mathrm{total microsomal protein}\;(mg)}\right)$$

The data was compared to a positive control, dextromethorphan, which exhibited a t1/2 of 40 minutes and an intrinsic clearance value of 31 ul/min/mg, consistent with literature values (McNaney et al, ASSAY and Drug Development Technologies, Bolume 6, Number 1, 2008).

#### Plasma Protein Binding

Human plasma protein binding was determined using TRANSIL^XL^ PPB plates (Sovicell, Leipzig, Germany). The compound 6jc48-1 (15 ul, 32% DMSO stock solution) was added to each well of a column (8 wells total) on a room temperature equilibrated plate. The plate was incubated for twelve minutes on a shaker at 1000 rpm then centrifuged for 10 minutes at 750 × *g* to sediment the beads from the suspension. Aliquots (100 ul) were transferred from the supernatants to 96-well plate for MS analysis. Plasma protein binding data analysis was completed by using the supplied spreadsheet from the manufacturer (Sovicell, User Guide TRANSIL PPB binding kit V2.01, 2013).

#### Plasma stability and Pharmacokinetic Study of 48–1

A solution of 6jc48-1was prepared at 2.5 mg/ml in 10% DMSO, 50% PEG in PBS and administered at 2.5 mg/kg intravenous (tail vein) to male CD1 mice (N = 3 per group). ~0.02 ml of blood was collected at 5 min, 15 min, 30 min, 1h, 2h, 4h, 6h, 8h and 14h post-treatment in centrifuge tubes containing 2 μl heparin (1,000 units). Compound was quantified by LC/MS/MS using working solutions of 10, 20, 50, 100, 500, 1,000, 5,000 and 10,000 ng/ml 6jc48-1 prepared in blank CD1 mouse plasma as internal standards. No adverse clinical observations were made for the duration of the experiment. For plasma stability measurements, 6jc48-1 (10 μg/ml) was incubated for 24 hours in the presence of serum (50%). Samples were taken after 2 min, 5min, 15min, 30min, 1h, 2h and 19h. Stability of compound was quantified by LC/MS/MS. Experiments were carried out by Pharmaron, Inc., (Beijing, China).

### Murine peritoneal sepsis model

#### Ethics statement

Care of the mice met or exceeded the standards set forth by the National Institute of Health Guide for the care and use of laboratory animals and the AVMA panel on Euthanasia. All procedures in this study have been approved by the Institutional Animal Care and Use Committee (IACUC) at the University of Maryland Baltimore School of Medicine (Protocol number 02122005). Adult C57BL/6J mice (~18 grams, 8–10 weeks old) were used for all experiments. Mice were obtained from the Jackson Laboratory (Bar Harbor, Maine, USA) and housed in the IHV SPC animal core facility. Mice were fed standard chow (Harlan Laboratories) and water ad libitum. To assess the protective potency of defensin mimetic 6jc48-1, groups of 5 mice were inoculated intraperitoneally ~ 5 x 10^8^ CFU/ animal of *Enterococcus faecalis* EF1509 in 500 μL saline solution plus 4.5% (w/v) porcine gastric mucin (Sigma Chemical Co., St. Louis, MO). Infected animals (*n = 5*) were subsequently treated by intraperitoneal injection 30 min post-infection with 100 mg/kg of compound in 300 μL sterile saline solution, 5% Tween 80, 10% DMSO (V/V), ampicillin (300mg/kg), or vehicle (sterile saline solution, 5% Tween 80, 10% DMSO (V/V)) as positive and negative controls, respectively. Animals were closely observed during a period of 24 h and mice that show signs of severe sepsis were humanely euthanized. Mice were anesthetized by intraperitoneal injection of ketamine (80-100mg/kg) and xylazine (10–15 mg/kg). Once sedated, blood was collected by cardiac puncture. Immediately after blood collection the mice were euthanized by cervical dislocation. Spleens were harvested aseptically, weighed and homogenized in 500 μl of sterile saline solution using an IKA T10 basic disperser (IKA, Wilmington NC). Spleen homogenates were serially diluted and plated onto BHI agar plates. Bacterial counts were determined following 24 h incubation at 37°C and expressed as CFU per gram for spleen.

## Results

### Ligand Design Strategy

**Fig 1:**
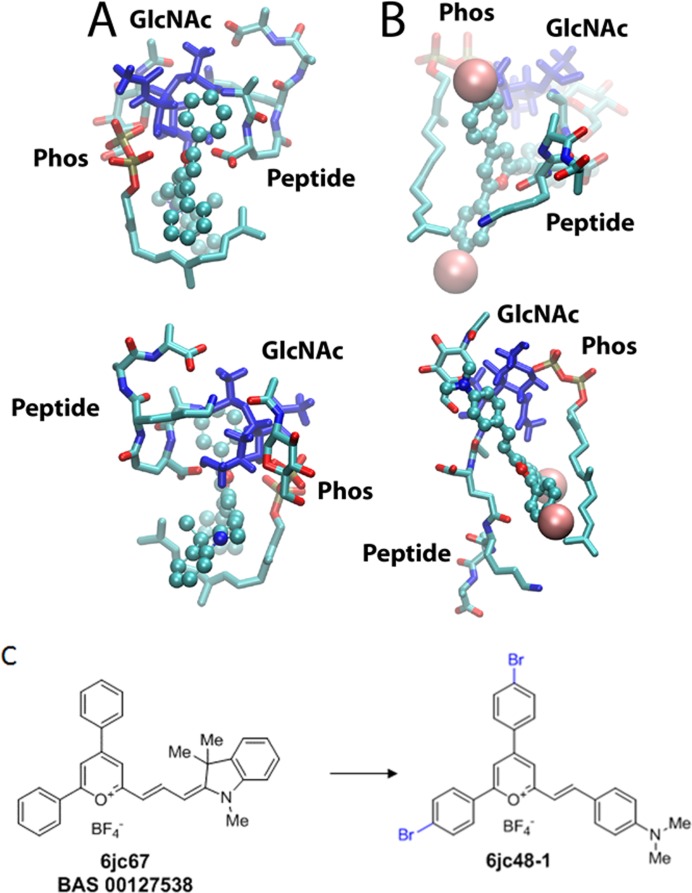
Models of BAS00127538 and 6jc48-1 in complex with a Lipid II analog. The compounds are shown in CPK atom colored format, with the Br atoms for 6jc48-1 shown as vdW spheres, and the Lipid II is in licorice representation with atom type coloring with the N-acetylglucoseamine sugars shown in blue. The phosphate (Phos), sugars (GlcNAc) and pentapeptide (Peptide) are indicated. The upper and lower panels are approximately 180° rotations of the two complexes. (C) Chemical structure of *de novo* synthesized BAS00127538 and the 6jc-48-1 derivative.

In our previous study a molecular model of the interaction of BAS00127538 with a lipid II analog was obtained that was consistent the NMR data obtained in that study [20]. That model is shown in Fig 1A with Lipid II. In the model the two phenyl rings and the pyrylium wrap around Lipid II with the positive charge of the pyrylium being in the vicinity of the Lipid II phosphates, one phenyl ring interacting with the sugar moiety and the second interacting with the top of the aliphatic tail. In addition, the indolene moiety also interacts with the aliphatic tail. Based on this interaction motif we hypothesized that increased hydrophobicity of the phenyl groups would lead to more favorable interactions with the sugar moiety and aliphatic tail of lipid II. Similarly, the presence and nature of the indolene was varied as well as the pyrylium to ring linker length and composition to understand their impact the SAR. Throughout the positively charged pyrilium was maintained given that in a previous study we showed the pyridinium was not active. This led to the design, synthesis and experimental validation of the compounds shown in the experimental supplement and S1 Table. Subsequent modeling of the top synthesized compound (6jc48-1) showed the binding orientation to be similar to that of BAS00127538 (Fig 1B and 1C), though some variation in the Lipid II conformation upon binding of compounds within the complex occurs. These include additional interactions of the bromophenyl moieties with the sugar and aliphatic moieties. In addition, the dimethylaniline analog in 6jc48-1 interacts with the peptidic portion of Lipid II and the ligand is shifted further away from the phosphate moieties. In contrast, the indolene moiety of BAS00127538 seemingly interacts with the aliphatic chain of the C55 only.

### General chemical synthesis

The general strategy for preparing new analogs of BAS00127538 is shown in Schemes 1, 2 and 3 in the methods section. This method allowed for independent variation of R^1^ and the R^2^ groups in the BAS00127538 scaffold (Scheme 3). We note that due to detailed description of the individual syntheses of compounds in this study is given in the S1 File.

### Functional characterization

The functional consequences of chemical modifications of the substituents around the di-phenyl pyrylium core were initially evaluated in two functional assays: **1**) anti-bacterial activity; **2**) Lipid II binding as assayed by Surface Plasmon Resonance; (**S1 Table**). Based on these functional assays, select compounds were further assayed for: **3**) broad-range antibacterial activity; **4**) Cellular cytotoxicity against mammalian cells (HeLa) expressed as CC_50_, the concentration at which cell viability is decreased by 50%. Measured Lipid II binding was also further qualified by determining the binding constant of these compounds. The data from these assays are presented in Table 1.

### Effects of modifications of the R^2^ indolene moiety

To explore the role of the indolene group on potency, toxicity and Lipid II binding, a series of analogs were synthesized in which the indolene moiety was replaced by varying aldehydes at the R^2^ position (Scheme 3 and Table 1). Based on anti-bacterial activity, substitution at the R^2^ position can be ranked from highest to lowest potency as: julolidine derivative (6jc65-1) > *N*-methyl-3-indolyl (6jc-53-2) > 3-indolyl (6jc53-2) > 4-dimethylaminophenyl (6jc51-1) > *N*,*N*-dimethyl-4-vinylaniline (6jc51-2). Antibacterial killing potency was correlated with cytotoxicity. Notably, with the exception of compounds 6jc51-1, 6jc58 and 6jc67A, all compounds displayed markedly reduced activity against Gram-negative species.

### Effect of modification on the R^1^ positions of the di-phenyl moiety

The effects of modification to the R^1^ positions at the two phenyl rings of the pyrlium core are summarized in Scheme 3 and Table 1. Compared to the parent scaffold, none of the R^1^ substitutions markedly enhanced potency or breadth of antibacterial activity. Irrespective of variations at the R^2^ position, *para*-methyl (cmpnds: 51–1, 51–2, 53–2, 65–1, 65–2), *meta*,*para*-dimethyl (cmpnds: 59–1, 59–2, 59–3, 66–4, 69–3) or *para*-ethyl (cmpnds: 64–1, 64–2, 64–3, 66–2, 69–1) at the R^1^ position retained antibacterial activity most potently. Substitution of the R^1^ moiety with *tert*-butyl, chloride or bromide in the *para* position significantly reduced antibacterial killing. Although in general antibacterial activity correlated with Lipid II and cellular cytotoxicity, the 48–1 and 48–2 compounds were a notable exception. These compounds revealed high affinity Lipid II binding and markedly reduced cellular cytotoxicity and a surprisingly specific anti-*Enteroccocci* activity.

**Fig 2:**
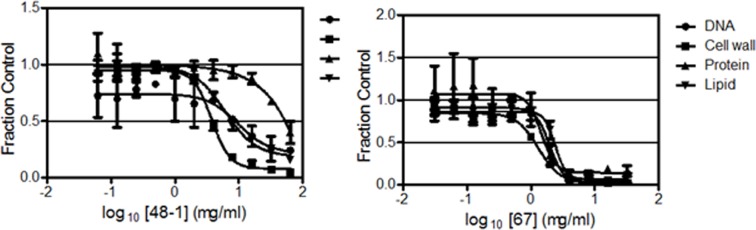
MMS analysis of 6jc48-1. The effects of 6jc48-1 (MIC 4 μg/ml) and Jc-67 (BAS00127538) (MIC 2 μg/ml) on the macromolecular synthetic pathways for DNA, Cell wall, protein, and lipid.

Based on its markedly decreased cytotoxicity (Table 1), 6jc48-1 was selected for further analysis. First, given its specific potency against *Enterococci*, 6jc48-1 was tested for potency against a wider array of *E*. *faecium* and *E*. *faecalis* strains (Table 2). The compound was effective in killing these drug-resistant strains, confirming its specific activity against these species. Next, to gain insight into its mechanism-of-action, MMS analyses were performed using the *E*. *faecalis* EF1509 strain (Fig 2). 6jc48-1 (MIC: 4 μg/ml) most potently inhibited cell wall synthesis (IC_50_ of 3.8 μg/ml), followed by inhibition of lipid and DNA synthesis (IC_50_ of 7.8 and 8.3 μg/ml respectively). Compound 6jc-67 (MIC: 2 μg/ml), the *de novo* synthesized parent BAS00127538 scaffold also most potently inhibited cell wall synthesis (IC_50_ of 1.1 μg/ml), followed by inhibition of DNA (IC_50_ of 1.8 μg/ml) and lipid synthesis (IC_50_ of 2.3 μg/ml), as previously reported for the commercially available chemical. Surprisingly, 6jc48-1 showed a markedly reduced inhibition of protein synthesis (IC_50_ of 50.4 μg/ml) compared to the JC-67, parent BAS00127538 scaffold (IC_50_ of 1.6 μg/ml).

Due to the presence of a pyrylium moiety in 6jc48-1, it is possible that the compound is reactive toward nucleophiles, including water, amines, and thiols. We therefore tested the drug-like properties of 6jc48-1 in *in vitro* assays. Analysis showed that the 6jc48-1 compound has favorable purity and solubility, liver microsome stability, and with the exception of CYP3A4/BFC, did not inhibit P450 enzyme activity at ≥10 μM. Further, Plasma protein binding was found to be 89% (Table 3). Due to incompatibility with solubilization conditions, membrane permeability and hepatotoxicity could not be determined (not shown).

**Table 3:** *In vitro* drug-like properties of 6jc48-1.

assay	6jc48-1
**Purity (LC-MS-MS)**	>95%
**Solubility (laser nephelometry)**	>50 μg/ml (n=3)
**Liver microsome stability (human; 1h 37** ^**o**^**C)**	Half-life >60 min;
	clearance <23 μl/min/mg
**Plasma protein binding (human, Transil**^**TM**^**)**	89%±3
**P450 enzyme inhibition (IC**_**50**_ **fluorescence)**	
**CYP3A4/ DBF**	>10 μM
**CYP3A4/ BFC**	1.3 μM
**CYP2D6/ AMMC**	>10 μM
**CYP2C19/CEC**	>10 μM

### *In vivo* stability of 6jc48-1

**Fig 3:**
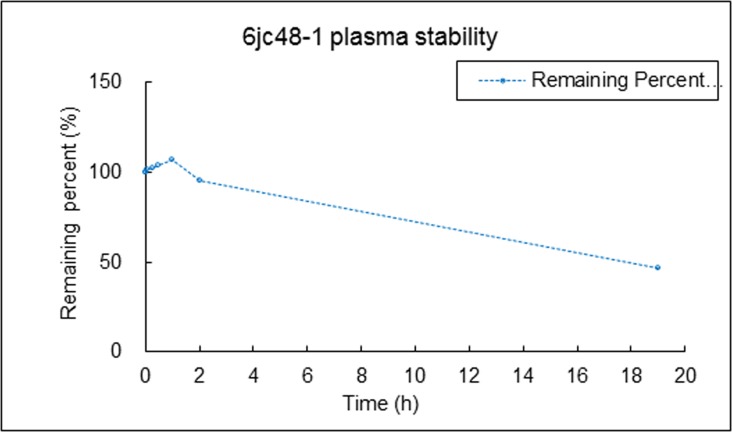
Compound 6jc48-1 plasma stability. Stability was tested by LC/MS/MS after 2 min, 5 min, 15 min, 30 min, 1h, 2h and 19h in the presence of 50% serum. Compound 6jc48-1 was fully stable after 2 hours in serum and after 19h, 46% of compound remained, indicating that plasma stability is long-lasting.

**Fig 4:**
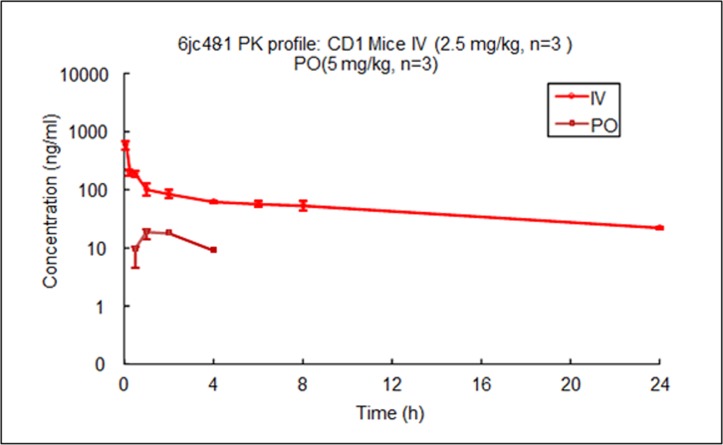
Pharmacokinetics of 6jc48-1 *in vivo*. Compound was administered at 2.5 mg/kg (IV) or 5 mg/kg (PO) to male CD1 mice (n = 3) in 10% DMSO and 50% PEG 400 in PBS. Half-life was determined by measuring the plasma concentration of compound by LC/MS/MS at the time points indicated.

We tested the chemical stability of 6jc48-1 directly *in vivo* by determining its stability in plasma as well as its pharmacokinetic profile. Compound plasma stability was tested by LC/MS/MS after 2 min, 5 min, 15 min, 30 min, 1h, 2h and 19h. Fig 3 shows that 6jc48-1 was stable after 2 hours in serum and after 19h, 46% of compound remained, indicating that plasma stability is long lasting. We next compared the pharmacokinetic (PK) profile of 6jc48-1 to parent BAS00127538 [35]. To determine the PK parameters, compounds were administered as a single dose of 2.5 mg/kg by intravenous injection or as a single oral dose of 5 mg/kg, and the plasma concentration over time was determined by LC/MS/MS. Upon intravenous administration, compound 6jc48-1 was very stable *in vivo* and could be readily detected after 4h (PO) or 24h (IV) (Fig 4). Based on these observations, the PK parameters were calculated for both compounds (Table 4). Compared to BAS00127538, compound 6jc48-1 showed markedly improved half-life (>13 h vs 0.22h), maximum concentration (1039 vs 101 ng/ml), increased volume of distribution (~23 vs 12.2 L/kg) and decreased clearance (23.6 vs 711 ml/min/kg). Upon oral administration, 6jc48-1 had a half-life of ~3h with a calculated bioavailability of ~2.5%, whereas compound BAS00127538 could not be detected (not shown).

**Table 4:** Pharmacokinetic properties of 6jc48-1.

	BAS00127538 (IV)	6jc48-1 (IV)	6jc48-1 (PO)
**T**_**1/2**_ **(h)**	0.227	13.3±1.8	2.78
**C**_**max**_ **(ng/mL)**	101	1039±323	19.1
**AUC**_**last**_ (h*ng/mL)	26.9	1340±117	46.9
**AUC**_**Inf**_ (h*ng/mL)	27.9	1769±120	90
**AUC**_**Extrap**_ **(%)**	4.38	24.3±3.1	48.6
**AUC**_**last**_/D (h*mg/mL)	26.9	536±47	9.4
**Vss_obs (L/Kg)**	12.2	22.8±2.9	1.75
**Cl_ob*s* (mL/min/Kg)**	711	23.6±1.7	NA
**MRT (h)**	0.226	7.29±0.27	NA
**F**_**last**_ **(%)**	NA	NA	1.9
**F**_**inf**_ **(%)**	NA	NA	2.54

T_1/2_: half-life; Cmax: Maximum observed concentration; AUC: area under the curve; D: Dose; Vss; volume of distribution; Cl: clearance; MRT: mean residence time; F: bioavailability

### *In vivo* efficacy of 6jc48-1

**Fig 5:**
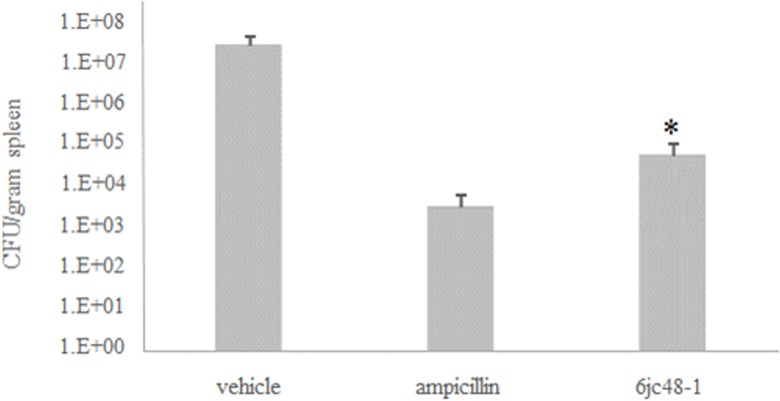
*In vivo* efficacy of 6jc48-1 in murine sepsis. Spleen samples were collected from vehicle-treated, ampicillin (300 mg/kg) treated or 6jc48-1 (100 mg/kg) treated animals at 20 h post-infection with 5 x 10^8^ CFU/animal of *E*. *faecalis* EF1509. * One animal treated with compound did not survive.

We established a murine model for sepsis to evaluate the efficacy of 6jc48-1 as an antibiotic agent *in vivo*. Preliminary maximum tolerated dose studies indicated that compound 6jc48-1 could be safely administered intraperitoneally at 100 mg/kg. Solubility of the compound restricted testing concentrations above 100 mg/kg (not shown). Based on the data obtained in the pharmacokinetic analysis, mice (n = 5) were inoculated intraperitoneally with *E*. *faecalis* EF1509 (~5 x 10^8^ CFU/ animal) and treated after 30 min with compound 6jc48-1 (100 mg/kg IP), ampicillin (300 mg/kg IP) or vehicle. Animals were monitored for survival and after 24 h spleen samples were collected and analyzed for the presence of bacteria. Bacterial counts were determined by plating serial dilutions on BHI agar plates and compared to control treatment with ampicillin as measures of efficacy (Fig 5). Animals treated with vehicle did not survive the length of the experiment. Four out of five animals treated with 6jc48-1 and all animals treated with ampicillin survived the length of the experiment. Bacterial counts measured in spleen revealed significant bacterial clearance in both cases, indicative of *in vivo* antibiotic efficacy.

## Discussion

In this study, we expand on our previous work in identifying and optimizing small molecule antagonists of Lipid II [20, 35, 36]. One of our most promising lead compounds, BAS00127538, was optimized to reduce cytotoxicity, increase *in vivo* stability and retain activity against *Enterococci* bacterial pathogens. In the United States, *Enterococci* infections in hospital settings are the second most common and vancomycin resistance is on the rise [37]. The observation that compound 6jc48-1 retained activity against *Enterococci spp*. specifically is somewhat surprising. The parent scaffold BAS00127538 is most potent against *S*. *aureus* and *Enterococci*, yet displays broad-range antibacterial activity, including activity against Gram-negative species [20, 36]. One possible explanation could be variations of Lipid II composition between different bacterial species [38]. For example, amidation of the D-iso-glutamine residue of Lipid II has been described in strains of S. *aureus* resulting in reduced sensitivity to the glycopeptides such as vancomycin [39, 40]. This modification has not been found in vancomycin-resistant *Enterococci spp* [41, 42]. Additional variations, such as differences in amino acid linkage between peptidoglycan subunits or variations in the MurNac/GlnNac moieties of Lipid II could further potentiate binding of the benzoaldehyde moiety of 6jc48-1, but not the indolene moiety in the BAS00127538 scaffold. This is consistent with our model of the 6jc48-1 interactions shown in Fig 1. More detailed structural studies of our small molecule antagonists in complex with Lipid II isolated from different species will be needed to answer these questions. The second major difference between parent BAS00127538 and 6jc48-1 is reduced cellular cytotoxicity. Mechanism-of-action studies revealed that 6jc48-1 does not inhibit protein synthesis to the same extent as BAS00127538. Since the incorporation of bromines in the *para* positions of the phenyl rings of the parent scaffold did not reduce cytotoxicity (6jcJC-67A), the indolene moiety in BAS00127538 likely contributes to cytotoxicity. Our model revealed similar interactions of parent scaffold and 6jc48-1 with Lipid II, possibly suggesting that the indolene moiety in the parent BAS00127538 molecule contributes to the interference of protein synthesis as a cause for cytotoxicity. Our *in vitro* and *in vivo* data indicate that the oxonium moiety in 6jc48-1 is chemically stable. In a previous study, we showed that replacement of the positively charged oxygen with nitrogen increased antibacterial activity and Lipid II binding, but did not lead to an improvement of cytotoxicity in the BAS00127538 scaffold [35]. Introducing this change in the 6jc48-1 scaffold could further enhance its antibacterial spectrum while maintaining low cytotoxicity.

## Conclusions

An SAR study of the small molecule Lipid II antagonist BAS00127538 has identified one compound, 6jc48-1, which displays improved drug-like properties compared to the parent scaffold. 6jc48-1 is stable and efficacious *in vivo*, has low toxicity and can be administered intravenously and orally. Molecular models of BAS00127538 and 6jc48-1 complexed with Lipid II, while qualitative in nature, indicate that the overall interaction pattern of the two compounds with Lipid II are similar, though specific differences are present, suggesting that further variations of the scaffold may lead to further improvements in activity. The 6jc48-1 scaffold, together with increased understanding of scaffold functionality that impact Lipid II interactions as well as bioavailability considerations, will facilitate the development of the first small molecule antibiotic that targets Lipid II.

## Supporting Information

S1 File Detailed description of the medicinal chemistry approaches and characterization of the compounds generated for this study. For each compound, scheme of synthesis, nomenclature and NMR is given.
(PDF)

S1 Table Chemical and functional overview of compounds generated in this study. For each compound, chemical structure, formula and molecular weight is provided. Additionally, compounds were assayed for binding to Lipid II by SPR and tested for activity against *S*. *aureus*.
(PDF)

## References

[1] Breukink E, de Kruijff B. Lipid II as a target for antibiotics. Nat Rev Drug Discov. 2006;5(4):321–32. Epub 2006/03/15. nrd2004 [pii] 10.1038/nrd2004 . 16531990

[2] Breukink E, Wiedemann I, van Kraaij C, Kuipers OP, Sahl H, de Kruijff B. Use of the cell wall precursor lipid II by a pore-forming peptide antibiotic. Science. 1999;286(5448):2361–4. Epub 1999/12/22. 8123 [pii]. . 10600751 10.1126/science.286.5448.2361

[3] den Blaauwen T, Andreu JM, Monasterio O. Bacterial cell division proteins as antibiotic targets. Bioorg Chem. 2014;55:27–38. 10.1016/j.bioorg.2014.03.007 . 24755375

[4] Schneider T, Sahl HG. Lipid II and other bactoprenol-bound cell wall precursors as drug targets. Curr Opin Investig Drugs. 2010;11(2):157–64. . 20112165

[5] Holland TL, Arnold C, Fowler VG Jr. Clinical management of Staphylococcus aureus bacteremia: a review. JAMA. 2014;312(13):1330–41. 10.1001/jama.2014.9743 25268440 PMC4263314

[6] Munita JM, Murray BE, Arias CA. Daptomycin for the treatment of bacteraemia due to vancomycin-resistant enterococci. Int J Antimicrob Agents. 2014;44(5):387–95. 10.1016/j.ijantimicag.2014.08.002 25261158 PMC4417356

[7] Cardona AF, Wilson SE. Skin and soft-tissue infections: a critical review and the role of telavancin in their treatment. Clin Infect Dis. 2015;61 Suppl 2:S69–78. 10.1093/cid/civ528 . 26316560

[8] Roberts KD, Sulaiman RM, Rybak MJ. Dalbavancin and Oritavancin: An Innovative Approach to the Treatment of Gram-Positive Infections. Pharmacotherapy. 2015;35(10):935–48. 10.1002/phar.1641 . 26497480

[9] Howden BP, Davies JK, Johnson PD, Stinear TP, Grayson ML. Reduced vancomycin susceptibility in Staphylococcus aureus, including vancomycin-intermediate and heterogeneous vancomycin-intermediate strains: resistance mechanisms, laboratory detection, and clinical implications. Clin Microbiol Rev. 2010;23(1):99–139. 10.1128/CMR.00042-09 20065327 PMC2806658

[10] O'Driscoll T, Crank CW. Vancomycin-resistant enterococcal infections: epidemiology, clinical manifestations, and optimal management. Infection and drug resistance. 2015;8:217–30. 10.2147/IDR.S54125 26244026 PMC4521680

[11] Tran TT, Munita JM, Arias CA. Mechanisms of drug resistance: daptomycin resistance. Ann N Y Acad Sci. 2015;1354(1):32–53. 10.1111/nyas.12948 . 26495887 PMC4966536

[12] Ganz T. Defensins: antimicrobial peptides of innate immunity. Nat Rev Immunol. 2003;3(9):710–20. 10.1038/nri1180 12949495

[13] de Leeuw E, Li C, Zeng P, Diepeveen-de Buin M, Lu WY, Breukink E, et al Functional interaction of human neutrophil peptide-1 with the cell wall precursor lipid II. FEBS Lett. 2010;584(8):1543–8. Epub 2010/03/11. S0014-5793(10)00198-5 [pii] 10.1016/j.febslet.2010.03.004 . 20214904 PMC3417325

[14] Schneider T, Kruse T, Wimmer R, Wiedemann I, Sass V, Pag U, et al Plectasin, a fungal defensin, targets the bacterial cell wall precursor Lipid II. Science. 2010;328(5982):1168–72. Epub 2010/05/29. 328/5982/1168 [pii] 10.1126/science.1185723 . 20508130

[15] Sass V, Schneider T, Wilmes M, Korner C, Tossi A, Novikova N, et al Human beta-defensin 3 inhibits cell wall biosynthesis in Staphylococci. Infect Immun. 2010;78(6):2793–800. Epub 2010/04/14. IAI.00688-09 [pii] 10.1128/IAI.00688-09 . 20385753 PMC2876548

[16] Schmitt P, Wilmes M, Pugniere M, Aumelas A, Bachere E, Sahl HG, et al Insight into invertebrate defensin mechanism of action: oyster defensins inhibit peptidoglycan biosynthesis by binding to lipid II. J Biol Chem. 2010;285(38):29208–16. Epub 2010/07/08. M110.143388 [pii] 10.1074/jbc.M110.143388 . 20605792 PMC2937951

[17] Oppedijk SF, Martin NI, Breukink E. Hit 'em where it hurts: The growing and structurally diverse family of peptides that target lipid-II. Biochim Biophys Acta. 2015 10.1016/j.bbamem.2015.10.024 . 26523408

[18] Essig A, Hofmann D, Munch D, Gayathri S, Kunzler M, Kallio PT, et al Copsin, a novel peptide-based fungal antibiotic interfering with the peptidoglycan synthesis. J Biol Chem. 2014;289(50):34953–64. 10.1074/jbc.M114.599878 25342741 PMC4263892

[19] Oeemig JS, Lynggaard C, Knudsen DH, Hansen FT, Norgaard KD, Schneider T, et al Eurocin, a new fungal defensin: structure, lipid binding, and its mode of action. J Biol Chem. 2012;287(50):42361–72. 10.1074/jbc.M112.382028 23093408 PMC3516779

[20] Varney KM, Bonvin AM, Pazgier M, Malin J, Yu W, Ateh E, et al Turning defense into offense: defensin mimetics as novel antibiotics targeting lipid II. PLoS Pathog. 2013;9(11):e1003732 10.1371/journal.ppat.1003732 24244161 PMC3820767

[21] Brooks BR, Brooks CL III, MacKerell AD Jr, Nilsson L, Petrella RJ, Roux B, et al CHARMM: the biomolecular simulation program. J Comput Chem. 2009;30(10):1545–614. Epub 2009/05/16. 10.1002/jcc.21287 19444816 PMC2810661

[22] Klauda JB, Venable RM, Freites JA, O'Connor JW, Tobias DJ, Mondragon-Ramirez C, et al Update of the CHARMM all-atom additive force field for lipids: validation on six lipid types. J Phys Chem B. 2010;114(23):7830–43. Epub 2010/05/26. 10.1021/jp101759q 20496934 PMC2922408

[23] MacKerell AD Jr, Bashford D, Bellott M, Dunbrack RL Jr, Evanseck J, Field MJ, et al All-atom empirical potential for molecular modeling and dynamics studies of proteins. J Phys Chem B. 1998;102:3586–616. 10.1021/jp973084f 24889800

[24] Best RB, Zhu X, Shim J, Lopes PEM, Mittal J, Feig M, et al Optimization of the additive CHARMM all-atom protein force field targeting improved sampling of the backbone φ, ψ and side-chain χ1 and χ2 dihedral angles. J Chem Theory and Comp. 2012;8:3257–73. . 10.1021/ct300400x PMC3549273 23341755

[25] Guvench O, Mallajosyula SS, Raman EP, Hatcher E, Vanommeslaeghe K, Foster TJ, et al CHARMM additive all-atom force field for carbohydrate derivatives and its utility in polysaccharide and carbohydrate-protein modeling. J Chem Theory Comput. 2011;7(10):3162–80. Epub 2011/11/30. 10.1021/ct200328p 22125473 PMC3224046

[26] Guvench O, Hatcher ER, Venable RM, Pastor RW, Mackerell AD. CHARMM Additive All-Atom Force Field for Glycosidic Linkages between Hexopyranoses. J Chem Theory Comput. 2009;5(9):2353–70. 10.1021/ct900242e 20161005 PMC2757763

[27] Jorgensen WL. Transferable Intermolecular Potential Functions for Waters, Alcohols, and Ethers. Application to Liquid Water. J Am Chem Soc. 1981;103:335.

[28] Vanommeslaeghe K, Hatcher E, Acharya C, Kundu S, Zhong S, Shim J, et al CHARMM general force field: A force field for drug-like molecules compatible with the CHARMM all-atom additive biological force fields. J Comp Chem. 2010;31(4):671–90. Epub 2009/07/04. 10.1002/jcc.21367 19575467 PMC2888302

[29] Vanommeslaeghe K, Mackerell AD Jr. Automation of the CHARMM General Force Field (CGenFF) I: Bond Perception and Atom Typing. J Chem Inf Model. 2012;52(12):3144–54. Epub 2012/11/14. 10.1021/ci300363c 23146088 PMC3528824

[30] Vanommeslaeghe K, Raman EP, MacKerell AD Jr. Automation of the CHARMM General Force Field (CGenFF) II: Assignment of Bonded Parameters and Partial Atomic Charges. J Chem Inf Model. 2012;52(12):3155–68. Epub 2012/11/14. 10.1021/ci3003649 23145473 PMC3528813

[31] Breukink E, van Heusden HE, Vollmerhaus PJ, Swiezewska E, Brunner L, Walker S, et al Lipid II is an intrinsic component of the pore induced by nisin in bacterial membranes. J Biol Chem. 2003;278(22):19898–903. 10.1074/jbc.M301463200 12663672

[32] CLSI. Methods for Dilution Antimicrobial Susceptibility Tests for Bacteria That Grow Aerobically; Approved Standard—Eighth Edition.2009.

[33] Butler MM, Lamarr WA, Foster KA, Barnes MH, Skow DJ, Lyden PT, et al Antibacterial activity and mechanism of action of a novel anilinouracil-fluoroquinolone hybrid compound. Antimicrob Agents Chemother. 2007;51(1):119–27. 10.1128/AAC.01311-05 17074800 PMC1797695

[34] Paradise E, Chaturvedi P, Ter-Ovanesyan E. Cytochrome P450 inhibition assays using traditional and fluorescent substrates. Curr Protoc Pharmacol. 2007;Chapter 7:Unit7 11. 10.1002/0471141755.ph0711s39 . 21948171

[35] Fletcher S, Yu W, Huang J, Kwasny SM, Chauhan J, Opperman TJ, et al Structure-activity exploration of a small-molecule Lipid II inhibitor. Drug Des Devel Ther. 2015;9:2383–94. 10.2147/DDDT.S79504 25987836 PMC4422293

[36] de Leeuw EP. Efficacy of the small molecule inhibitor of Lipid II BAS00127538 against Acinetobacter baumannii. Drug Des Devel Ther. 2014;8:1061–4. 10.2147/DDDT.S68020 25143710 PMC4134019

[37] Arias CA, Murray BE. The rise of the Enterococcus: beyond vancomycin resistance. Nat Rev Microbiol. 2012;10(4):266–78. 10.1038/nrmicro2761 22421879 PMC3621121

[38] van Heijenoort J. Lipid intermediates in the biosynthesis of bacterial peptidoglycan. Microbiol Mol Biol Rev. 2007;71(4):620–35. Epub 2007/12/08. 71/4/620 [pii] 10.1128/MMBR.00016-07 . 18063720 PMC2168651

[39] Munch D, Roemer T, Lee SH, Engeser M, Sahl HG, Schneider T. Identification and in vitro analysis of the GatD/MurT enzyme-complex catalyzing lipid II amidation in Staphylococcus aureus. PLoS Pathog. 2012;8(1):e1002509 10.1371/journal.ppat.1002509 22291598 PMC3266927

[40] Hanaki H, Labischinski H, Inaba Y, Kondo N, Murakami H, Hiramatsu K. Increase in glutamine-non-amidated muropeptides in the peptidoglycan of vancomycin-resistant Staphylococcus aureus strain Mu50. J Antimicrob Chemother. 1998;42(3):315–20. . 9786471 10.1093/jac/42.3.315

[41] Allen NE, Hobbs JN Jr, Nicas TI. Inhibition of peptidoglycan biosynthesis in vancomycin-susceptible and -resistant bacteria by a semisynthetic glycopeptide antibiotic. Antimicrob Agents Chemother. 1996;40(10):2356–62. 8891144 10.1128/aac.40.10.2356 PMC163534

[42] Belley A, Harris R, Beveridge T, Parr T Jr, Moeck G. Ultrastructural effects of oritavancin on methicillin-resistant Staphylococcus aureus and vancomycin-resistant Enterococcus. Antimicrob Agents Chemother. 2009;53(2):800–4. 10.1128/AAC.00603-08 19029329 PMC2630643

