# Supplementary material for: Towards Development of Small Molecule Lipid II Inhibitors as Novel Antibiotics
Source: PLoS One. 2016 Oct 24;11(10):e0164515. doi: 10.1371/journal.pone.0164515 (PMC5077133; doi:10.1371/journal.pone.0164515)
Supplement: S1 File — For each compound, scheme of synthesis, nomenclature and NMR is given. (PDF) [file pone.0164515.s001.pdf]

Experimental supplement

General procedure for pyrylium salt synthesis

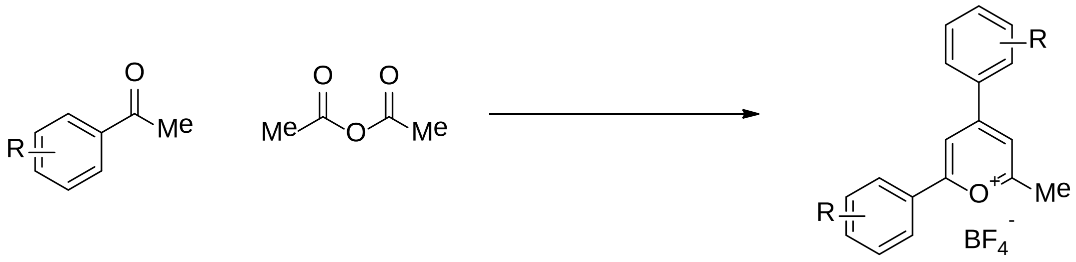

To substituted acetophenone and acetic anhydride was added boron trifluoride etherate (32.0 mmol) at room temperature. The reaction was heated to 135 degrees C for 4 h, cooled and poured into EtOAc and allowed to stand for 1 h. The yellow solid was filtered and washed with excess EtOAc to give the title compounds as the boron tetrafluoride salt.

General procedure for the condensation reaction with aldehydes

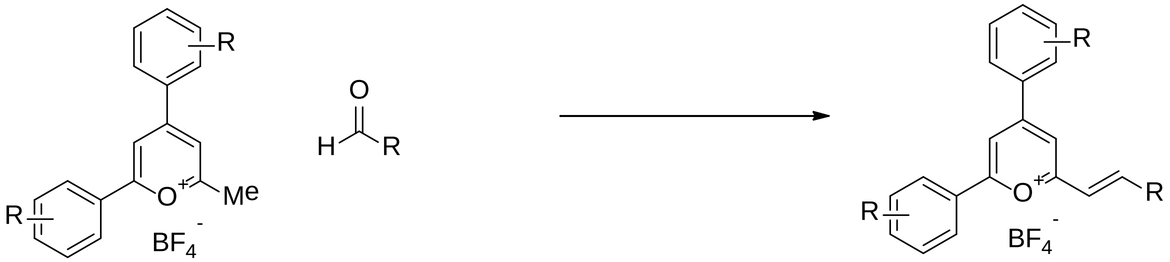

Pyrylium salt (0.28 mmol) and aldehyde (0.34 mmol) in MeOH (8 mL) was heated to reflux for 4 h. The reaction was cooled, reduced in vacuo, poured into EtOAc and allowed to stand for 1 h. The dark solid was filtered and washed with excess EtOAc to give the title compounds as the boron tetrafluoride salt.

2-methyl-4,6-diphenylpyrylium boron tetrafluoride salt **6jc26**

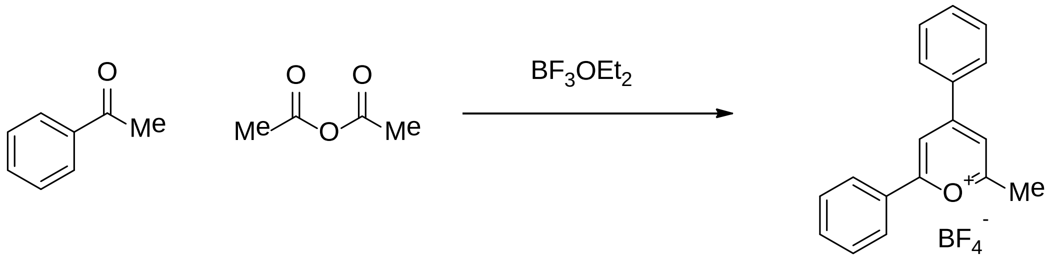

6jc26

To acetophenone (2 g, 16.67 mmol) and acetic anhydride (1.57 mL, 16.67 mmol) was added boron trifluoride etherate (3.40 mL, 22.50 mmol) at room temperature. The reaction was heated to 135 degrees C for 2 hr, cooled, poured into EtOAc and the yellow solid filtered. Recrystallization from AcOH gave yellow solid 813 mg.

δ_H(MeOH-*d4*, 400 MHz) 8.95 (s, 1 H, Ar), 8.43 (s, 1 H, Ar), 8.41 (d, 2 H, J = 8.0), 8.30 (d, 2 H, J = 8.0), 7.82 (t, 2 H, J = 7.2, Ar), 7.73 (t, 4 H, J = 7.6, Ar), 3.04 (s, 3 H, Me, exchanges with deuterium over time)

2,4-bis(4-chlorophenyl)-6-methylpyrylium boron tetrafluoride salt **6jc32-1**

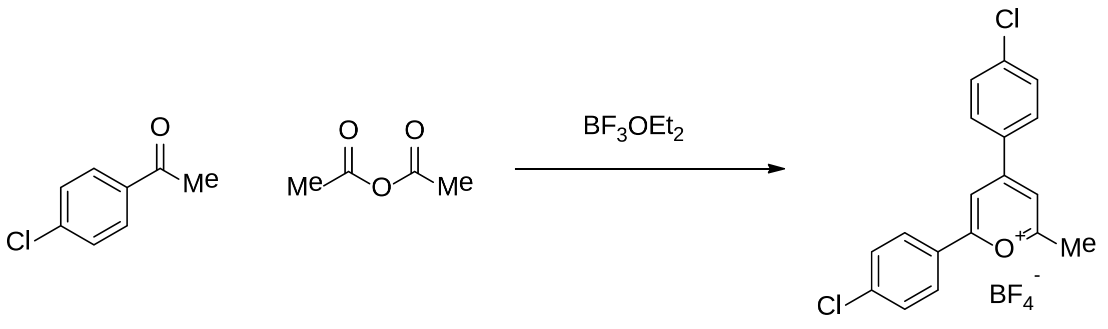

6jc32-1

To p-chloro-acetophenone (2 g, 13.0 mmol) and acetic anhydride (1.22 mL, 13.0 mmol) was added boron trifluoride etherate (4 mL, 32.0 mmol) at room temperature. The reaction was heated to 135 degrees C for 2 hr, cooled, poured into EtOAc and the yellow solid filtered to give 644 mg.

δ_H(MeOH-*d4*, 400 MHz) 8.95 (s, 1 H, Ar), 8.44 (s, 1 H, Ar), 8.40 (d, 2 H, J = 8.8), 8.29 (d, 2 H, J = 8.8), 7.75 (d, 4 H, J = 7.2, Ar), 3.04 (s, 3 H, Me).

2,4-bis(4-methoxyphenyl)-6-methylpyrylium boron tetrafluoride salt **6jc32-2**

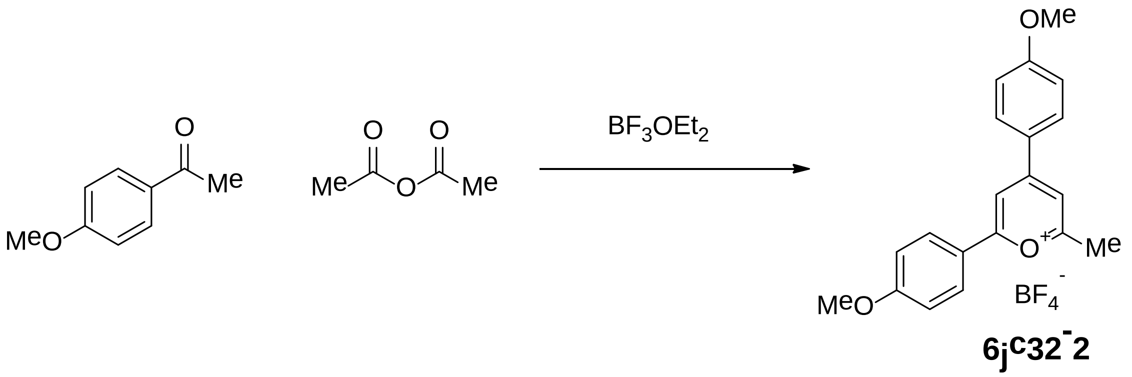

6jc32-2

To p-methoxy-acetophenone (2 g, 13.0 mmol) and acetic anhydride (1.22 mL, 13.0 mmol) was added boron trifluoride etherate (4 mL, 32.0 mmol) at room temperature. The reaction was heated to 135 degrees C for 2 hr, cooled, poured into EtOAc and the red solid filtered to give 313 mg.

δ_H(MeOH-d_4, 400 MHz) 8.67 (s, 1 H, Ar), 8.44 (s, 1 H, Ar), 8.37 (d, 2 H, J = 9.6), 8.32 (d, 2 H, J = 9.2), 8.15 (s, 1 H, Ar), 7.25-7.21 (m, 4 H, Ar), 3.97 (s, 3 H, OMe), 3.96 (s, 3 H, OMe), 2.91 (s, 3 H, Me).

2,4,6-trimethylpyrylium boron tetrafluoride salt **6jc36**

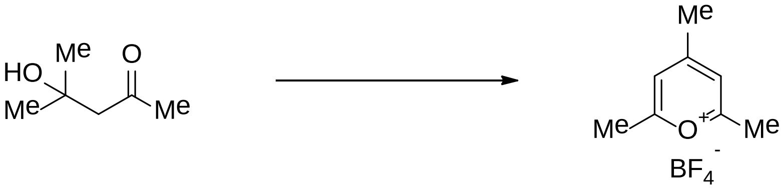

6jc36

To pentanone (1.16 g, 10.0 mmol) and acetic anhydride (9.5 mL, 100.0 mmol) was added boron trifluoride etherate (2.5 mL, 10.0 mmol) at room temperature. The reaction was heated to 135 degrees C for 2 hr, cooled, poured into EtOAc and the white solid filtered to give 980 mg.

δ_H(MeOH-d_4, 400 MHz) 7.86 (s, 2 H, Ar), 2.89 (s, 6 H, 2 x Me), 2.70 (s, 3 H, Me).

(E)-4-(4-(dimethylamino)styryl)-2,6-dimethylpyrylium boron tetrafluoride salt **6jc37**

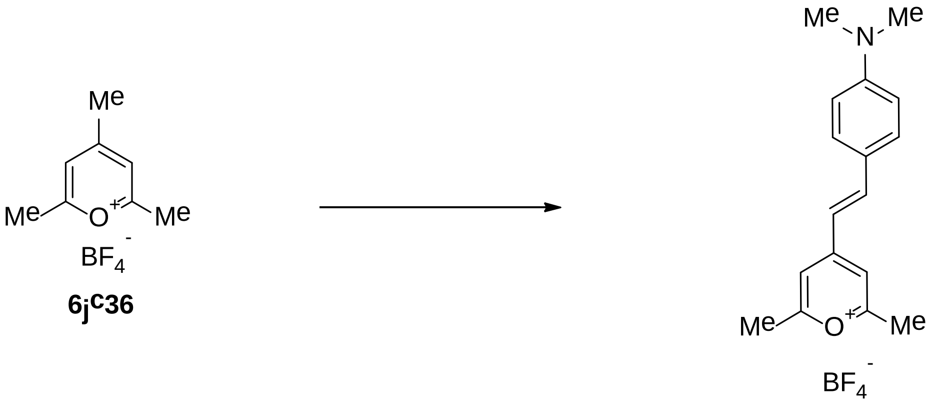

6jc37

To pyrylium salt **6jc36** (210 mg, 1.00 mmol) and p-dimethylamino benzaldehyde (150 mg, 1.00 mmol) in MeOH (10 mL) was stirred at room temperature overnight. The solvent was removed and the residue was suspended in ether and filtered to give blue solid. Recrystallization from EtOH gave blue solid of 110 mg.

δ_H(DMSO-d_6, 400 MHz) 8.35 (d, 1 H, J = 14.8, H-vinyl), 7.76 (d, 2 H, J = 8.4, Ar), 7.69 (s, 2 H, Ar), 7.13 (d, 1 H, J = 14.8, H-vinyl), 6.91 (d, 2 H, J = 8.4, Ar), 3.16 (s, 6 H, 2 x Me), 2.63 (s, 6 H, NMe_2).

4-((1E,3E)-4-(4-(dimethylamino)phenyl)buta-1,3-dien-1-yl)-2,6-dimethylpyrylium boron tetrafluoride salt **6jc38**

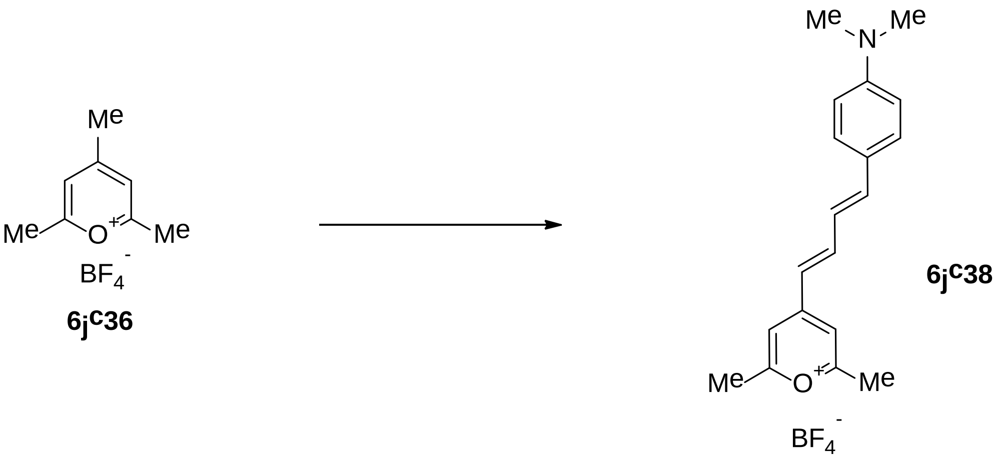

To pyrylium salt **6jc36** (210 mg, 1.00 mmol) and p-dimethylamino benzaldehyde (176 mg, 1.00 mmol) in MeOH (10 mL) was stirred at room temperature overnight. The solvent was removed and the residue was suspended in ether and filtered to give blue solid. Recrystallization from EtOH gave green solid of 110 mg.

δ_H(DMSO-d_6, 400 MHz) 8.25 (dd, 1 H, J = 14.8, 14.4, H-vinyl), 7.73 (s, 2 H, Ar), 7.65 (d, 2 H, J = 8.8, Ar), 7.39 (d, 1 H, J = 14.8, H-vinyl), 7.27 (t, 1 H, J = 14.8, H-vinyl), 6.81 (d, 2 H, J = 8.8, Ar), 6.63 (d, 1 H, J = 14.4, H-vinyl), 3.10 (s, 6 H, 2 x Me), 2.64 (s, 6 H, NMe_2).

(E)-2-(4-(dimethylamino)styryl)-4,6-diphenylpyrylium boron tetrafluoride salt **6jc39**

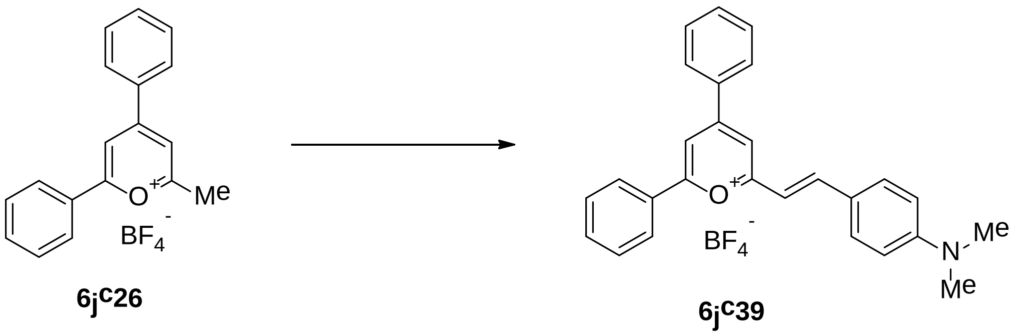

Pyrylium salt **6jc26** (100 mg, 0.30 mmol) and p-dimethylamino benzaldehyde (50 mg, 0.32 mmol) in MeOH (6 mL) was stirred at room temperature overnight. The solvent removed and the residue was suspended in ether and filtered to give green solid of 81 mg.

δ_H(DMSO-d_6, 400 MHz) 8.62 (s, 1 H, Ar), 8.51-8.42 (m, 4 H, Ar, HC=), 8.34 (d, 2 H, J = 7.2, Ar), 7.86 (d, 2 H, J = 9.6, Ar), 7.84-7.70 (m, 6 H, Ar), 7.42 (d, 1 H, J = 15.6, HC=), 6.93 (d, 2 H, J = 8.4, Ar), 3.17 (s, 6 H, 2 x Me).

(E)-2,4-bis(4-chlorophenyl)-6-(4-(dimethylamino)styryl)pyrylium boron tetrafluoride salt **6jc41-1**

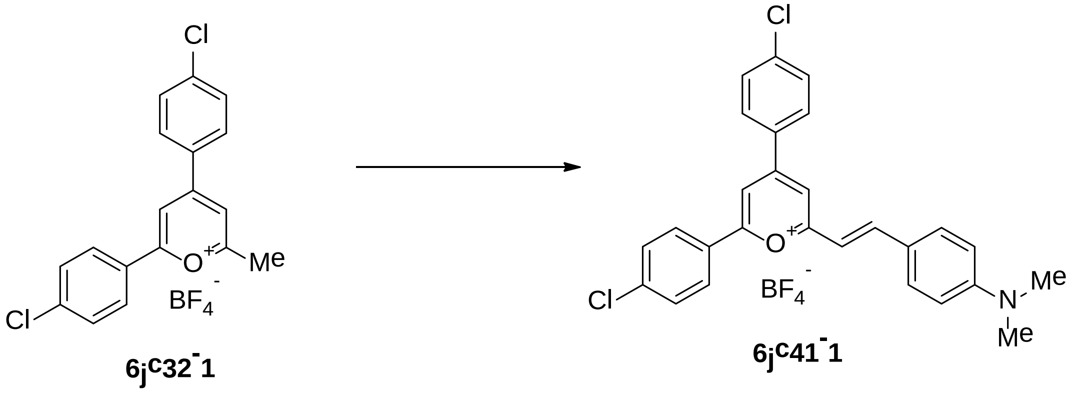

6jc32-1 6jc41-1

Pyrylium salt **6jc32-1** (134 mg, 0.33 mmol) and p-dimethylamino benzaldehyde (50 mg, 0.33 mmol) in MeOH (6 mL) was stirred at room temperature overnight. The solvent removed and the residue was suspended in ether and filtered to give dark blue solid of 105 mg.

δ_H(DMSO-d_6, 400 MHz) 8.57 (s, 1 H, Ar), 8.49-8.40 (m, 4 H, Ar, HC=), 8.34 (d, 2 H, J = 8.4, Ar), 7.84-7.77 (m, 6 H, Ar), 7.37 (d, 1 H, J = 15.6, HC=), 6.92 (d, 2 H, J = 8.4, Ar), 3.17 (s, 6 H, 2 x Me).

2-((1E,3E)-4-(4-(dimethylamino)phenyl)buta-1,3-dien-1-yl)-4,6-diphenylpyrylium boron tetrafluoride salt **6jc43-1**

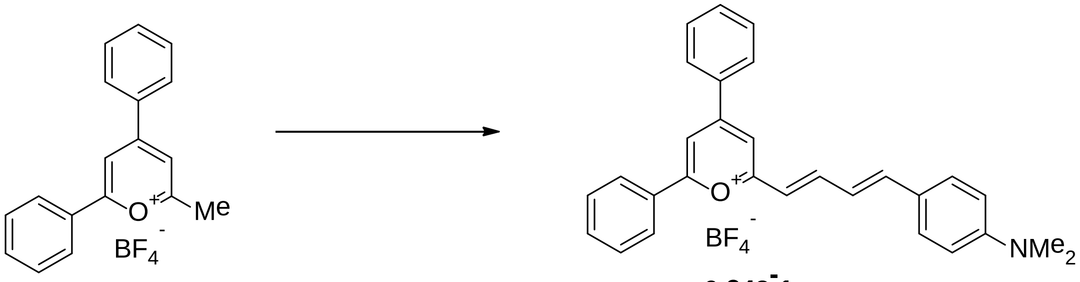

6jc26 6jc43-1

Pyrylium salt **6jc26** (94 mg, 0.28 mmol) and p-dimethylamino cinnamaldehyde (60 mg, 0.34 mmol) in MeOH (8 mL) was heated to reflux for 2 hrs. The reaction was cooled, solvent removed and the residue was suspended in ether, washed with EtOAc and filtered to give dark blue solid of 55 mg.

δ_H(DMSO-d_6, 400 MHz) 8.67 (s, 1 H, Ar), 8.54-8.44 (m, 3 H, Ar), 8.36 (d, 2 H, J = 8.0, Ar), 8.31 (t, 1 H, J = 14.8, H-vinyl), 7.85-7.70 (m, 6 H, Ar), 7.65 (d, 2 H, J = 8.4, Ar), 7.57 (d, 1 H, J = 14.8, H-vinyl), 7.31 (t, 1 H, J = 14.8, H-vinyl), 6.88 (d, 1 H, J = 14.8, H-vinyl), 6.83 (d, 2 H, J = 8.4, Ar), 3.09 (s, 6 H, NMe_2).

2,4-bis(4-chlorophenyl)-6-((1E,3E)-4-(4-(dimethylamino)phenyl)buta-1,3-dien-1-yl)pyrylium boron tetrafluoride salt **6jc43-2**

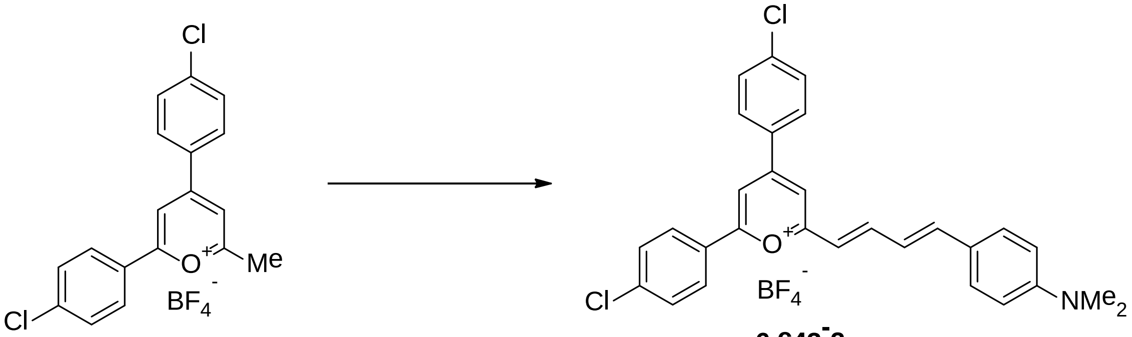

6jc32-1 6jc43-2

Pyrylium salt **6jc32-1** (112 mg, 0.28 mmol) and p-dimethylamino cinnamaldehyde (60 mg, 0.34 mmol) in MeOH (8 mL) was heated to reflux for 2 hrs. The reaction was cooled, solvent removed and the residue was suspended in ether, washed with EtOAc and filtered to give dark blue solid of 46 mg.

δ_H(DMSO-d_6, 400 MHz) 8.62 (s, 1 H, Ar), 8.54-8.44 (m, 3 H, Ar), 8.37 (d, 2 H, J = 7.2, Ar), 8.28 (t, 1 H, J = 13.6, H-vinyl), 7.89-7.72 (m, 4 H, Ar), 7.64 (d, 2 H, J = 8.0, Ar), 7.55 (d, 1 H, J = 15.2, H-vinyl), 7.30 (t, 1 H, J = 13.2, H-vinyl), 6.69-6.73 (m, 3 H, Ar, H-vinyl), 3.10 (s, 6 H, NMe_2).

2,4-bis(4-bromophenyl)-6-methylpyrylium boron tetrafluoride salt **6jc47**

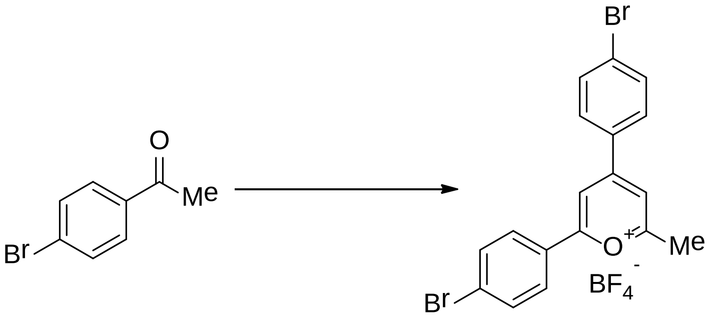

6jc47

To p-bromo-acetophenone (2.42 g, 12.12 mmol) and acetic anhydride (1.14 mL, 12.12 mmol) was added boron trifluoride etherate (3.68 mL, 32.0 mmol) at room temperature. The reaction was heated to 135 degrees C for 2 hr, cooled, poured into EtOAc and the yellow solid filtered to give 705 mg.

δ_H(MeOH-d_4, 400 MHz) 8.95 (s, 1 H, Ar), 8.45 (s, 1 H, Ar), 8.30 (d, 2 H, J = 8.8, Ar), 8.20 (d, 2 H, J = 8.8), 8.00-7.86 (m, 4 H, Ar), 3.03 (s, 3 H, Me).

(E)-2,4-bis(4-bromophenyl)-6-(4-(dimethylamino)styryl)pyrylium boron tetrafluoride salt **6jc48-1**

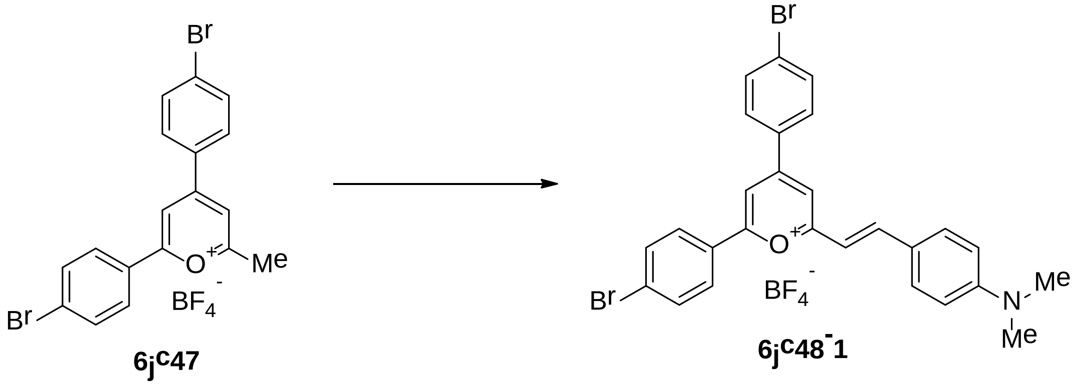

To pyrylium salt **6jc47** (100 mg, 0.20 mmol) and p-dimethylamino benzaldehyde (32 mg, 0.20 mmol) in MeOH (10 mL) was stirred at room temperature overnight. The solvent was removed

and the residue was suspended in ether and filtered to give blue solid. Recrystallization from EtOH gave blue solid of 95 mg.

δ_H(DMSO-d_6, 400 MHz) 8.56 (s, 1 H, Ar), 8.47 (s, 1 H, Ar), 8.43-8.38 (m, 3 H, Ar, HC=), 8.27 (d, 2 H, J = 8.4, Ar), 8.00-7.90 (m, 4 H, Ar), 7.84 (d, 2 H, J = 8.4, Ar), 7.39 (d, 1 H, J = 15.6, HC=), 6.94 (d, 2 H, J = 8.8, Ar), 3.18 (s, 6 H, 2 x Me).

2,4-bis(4-bromophenyl)-6-((1E,3E)-4-(4-(dimethylamino)phenyl)buta-1,3-dien-1-yl)pyrylium boron tetrafluoride salt **6jc48-2**

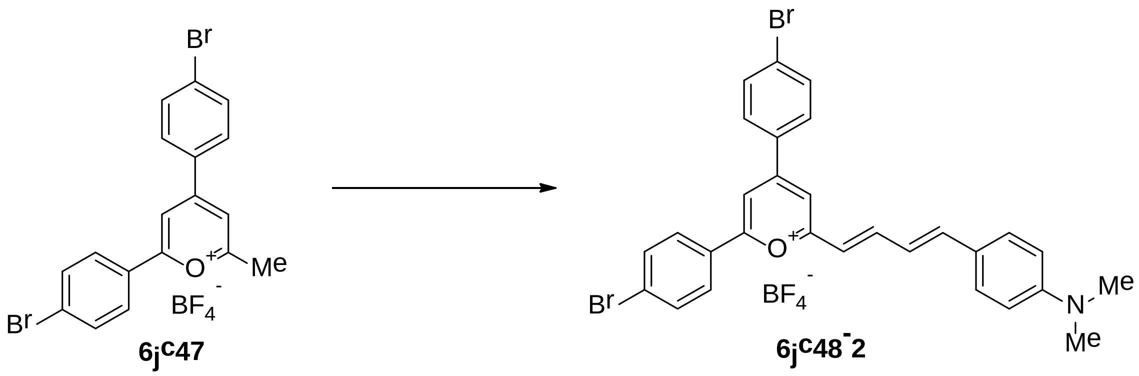

6jc47 **6jc48-2**

To pyrylium salt **6jc47** (100 mg, 0.20 mmol) and p-dimethylamino cinnamaldehyde (38 mg, 0.20 mmol) in MeOH (10 mL) was stirred at room temperature overnight. The solvent was removed and the residue was suspended in ether and filtered to give blue solid. Recrystallization from EtOH gave blue solid of 41 mg.

δ_H(DMSO-d_6, 400 MHz) 8.64 (s, 1 H, Ar), 8.49 (s, 1 H, Ar), 8.39 (d, 2 H, J = 8.4, Ar), 8.35-8.24 (m, 3 H, Ar, HC=), 8.00-7.89 (m, 4 H, Ar), 7.65 (d, 2 H, J = 8.4, Ar), 7.56 (d, 1 H, J = 14.8, HC=), 7.32 (t, 1 H, J = 14.8, HC=), 6.90-6.81 (m, 3 H, Ar, HC=), 3.10 (s, 6 H, NMe_2).

(E)-2-(2-(1H-indol-3-yl)vinyl)-4,6-diphenylpyrylium boron tetrafluoride salt **6jc49-1**

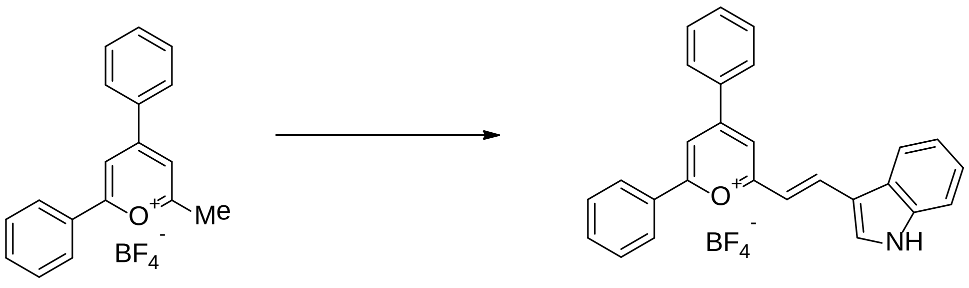

6jc26 **6jc49-1**

To pyrylium salt **6jc26** (100 mg, 0.30 mmol) and indole-3-carboxaldehyde (45 mg, 0.30 mmol) in MeOH (8 mL) was stirred at room temperature overnight. The solvent was removed and the residue was suspended in ether and filtered to give blue solid. Recrystallization from EtOH gave blue solid of 32 mg.

δ_H(DMSO-d_6, 400 MHz) 12.65 (br s, 1 H, NH), 8.75 (d, 1 H, J = 16.4, HC=), 8.64 (s, 1 H, Ar), 8.60 (s, 1 H, Ar), 8.53 (d, 2 H, J = 7.6, Ar), 8.47 (s, 1 H, Ar), 8.40-8.26 (m, 3 H, Ar), 7.86-7.70 (m, 6 H, Ar), 7.66-7.60 (m, 1 H, Ar), 7.54 (d, 1 H, J = 16.4, HC=), 7.45-7.36 (m, 2 H, Ar).

2-methyl-4,6-di-p-tolylpyrylium boron tetrafluoride salt **6jc50-2**

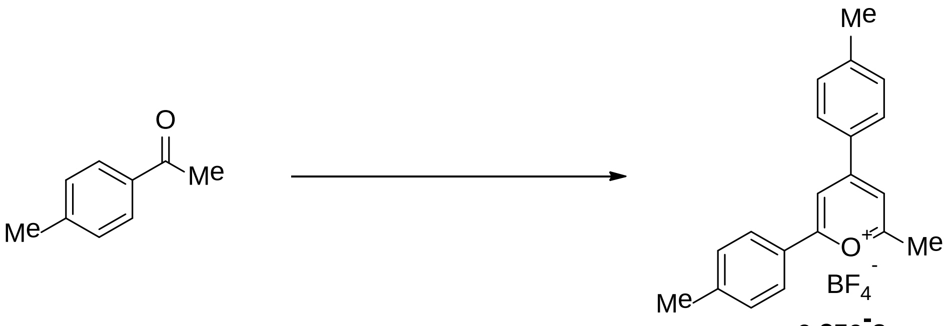

6jc50-2

To p-methyl-acetophenone (2.00 g, 15.0 mmol) and acetic anhydride (1.40 mL, 15.0 mmol) was added boron trifluoride etherate (4.55 mL, 36.9 mmol) at room temperature. The reaction was heated to 135 degrees C for 2 hr, cooled, poured into EtOAc and the yellow solid filtered to give 609 mg.

δ_H(MeOH-d_4, 400 MHz) 8.83 (s, 1 H, Ar), 8.32 (s, 1 H, Ar), 8.29 (d, 2 H, J = 8.0, Ar), 8.20 (d, 2 H, J = 8.0, Ar), 7.59-7.50 (m, 4 H, Ar), 2.98 (s, 3 H, Me), 2.50 (s, 6 H, 2 x Me).

(E)-2-(4-(dimethylamino)styryl)-4,6-di-p-tolylpyrylium boron tetrafluoride salt **6jc51-1**

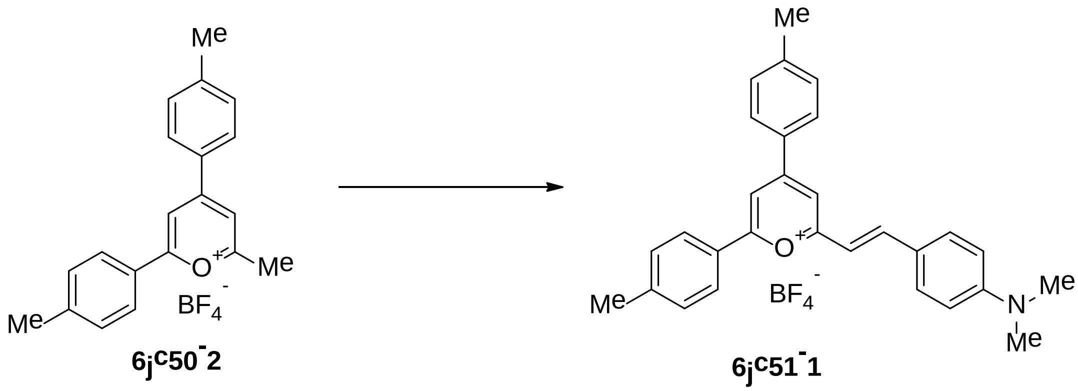

6jc50-2 **6jc51-1**

To pyrylium salt **6jc50-2** (100 mg, 0.27 mmol) and p-dimethylamino benzaldehyde (43 mg, 0.27 mmol) in MeOH (8 mL) was stirred at room temperature overnight. The solvent was removed and the residue was suspended in ether and filtered to give blue solid. Recrystallization from EtOH gave blue solid of 73 mg.

δ_H(DMSO-d_6, 400 MHz) 8.58 (s, 1 H, Ar), 8.43 (s, 1 H, Ar), 8.42-8.35 (m, 3 H, Ar, HC=), 8.28 (d, 2 H, J = 8.0, Ar), 7.83 (d, 2 H, J = 8.8, Ar), 7.58-7.50 (m, 4 H, Ar), 7.38 (d, 1 H, J = 15.6, HC=), 6.91 (d, 2 H, J = 8.8, Ar), 3.15 (s, 6 H, NMe_2), 2.49 (s, 6 H, 2 x Me).

2-((1E,3E)-4-(4-(dimethylamino)phenyl)buta-1,3-dien-1-yl)-4,6-di-p-tolylpyrylium boron tetrafluoride salt **6jc51-2**

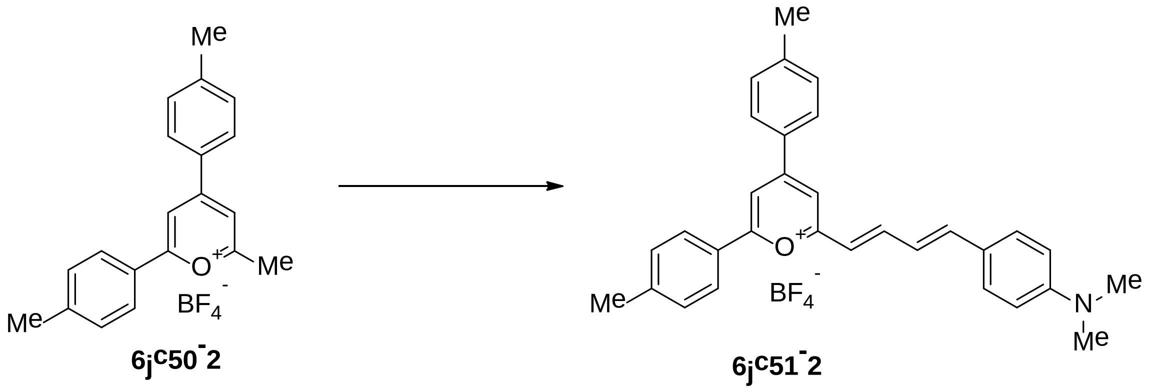

6jc50-2 **6jc51-2**

To pyrylium salt **6jc50-2** (100 mg, 0.27 mmol) and p-dimethylamino cinnamaldehyde (50 mg, 0.27 mmol) in MeOH (8 mL) was stirred at room temperature overnight. The solvent was removed and the residue was suspended in ether and filtered to give blue solid. Recrystallization from EtOH gave blue solid of 58 mg.

δ_H(DMSO-d_6, 400 MHz) 8.63 (s, 1 H, Ar), 8.44 (s, 1 H, Ar), 8.39 (d, 2 H, J = 7.6, Ar), 8.31 (d, 2 H, J = 7.6, Ar), 8.24 (t, 1 H, HC=), 7.62 (d, 2 H, J = 8.8, Ar), 7.58-7.43 (m, 5 H, Ar, HC=), 7.27 (t, 1 H, J = 14.8, HC=), 6.90-6.78 (m, 3 H, Ar, HC=), 3.08 (s, 6 H, NMe_2), 2.49 (s, 6 H, 2 x Me).

(E)-2-(2-(1H-indol-3-yl)vinyl)-4,6-bis(4-chlorophenyl)pyrylium boron tetrafluoride salt **6jc53-1**

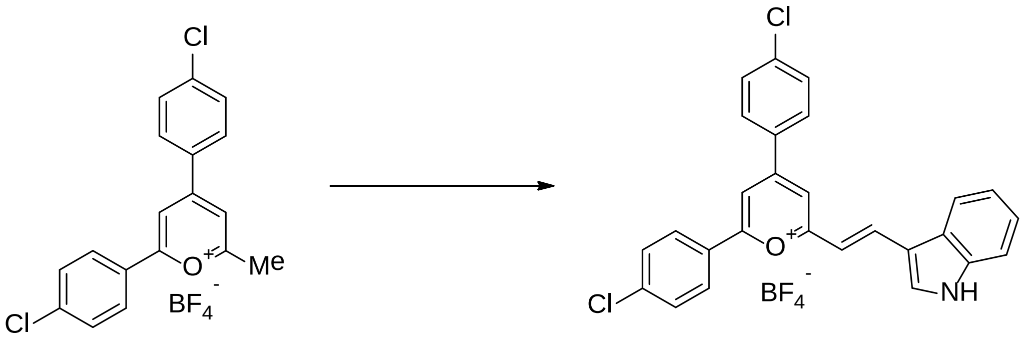

6jc32-1 6jc53-1

To pyrylium salt **6jc32-1** (138 mg, 0.34 mmol) and indole-3-carboxaldehyde (50 mg, 0.34 mmol) in MeOH (8 mL) was stirred at room temperature overnight. The solvent was removed and the residue was suspended in ether and filtered to give blue solid. Recrystallization from EtOH gave purple solid of 53 mg.

δ_H(DMSO-*d*$_6$, 400 MHz) 12.70 (br s, 1 H, NH), 8.78 (d, 1 H, J = 14.8, HC=), 8.64 (s, 1 H, Ar), 8.61 (s, 1 H, Ar), 8.53 (d, 2 H, J = 8.4, Ar), 8.47 (s, 1 H, Ar), 8.37 (d, 2 H, J = 8.8, Ar), 8.31-8.26 (m, 1 H, Ar), 7.89-7.80 (m, 4 H, Ar), 7.68-7.60 (m, 1 H, Ar), 7.52 (d, 1 H, J = 14.8, HC=), 7.45-7.36 (m, 2 H, Ar).

(E)-2-(2-(1H-indol-3-yl)vinyl)-4,6-di-p-tolylpyrylium boron tetrafluoride salt **6jc53-2**

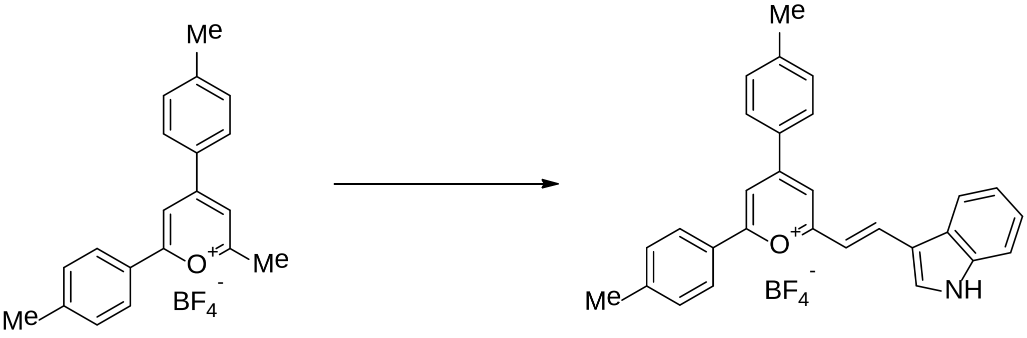

6jc50-2 6jc53-2

To pyrylium salt **6jc50-2** (123 mg, 0.34 mmol) and indole-3-carboxaldehyde (50 mg, 0.34 mmol) in MeOH (8 mL) was stirred at room temperature overnight. The solvent was removed and the residue was suspended in ether and filtered to give blue solid. Recrystallization from EtOH gave purple solid of 50 mg.

δH(DMSO-*d6*, 400 MHz) 12.55 (br s, 1 H, NH), 8.68 (d, 1 H, J = 15.6 HC=), 8.60 (s, 1 H, Ar), 8.55 (s, 1 H, Ar), 8.46-8.40 (m, 3 H, Ar), 8.30-8.22 (m, 3 H, Ar), 7.66-7.53 (m, 5 H, Ar), 7.50 (d, 1 H, J = 15.6, HC=), 7.43-7.35 (m, 2 H, Ar), 2.50 (2 x Me).

(E)-2-(2-(1H-indol-3-yl)vinyl)-4,6-bis(4-bromophenyl)pyrylium boron tetrafluoride salt **6jc53-3**

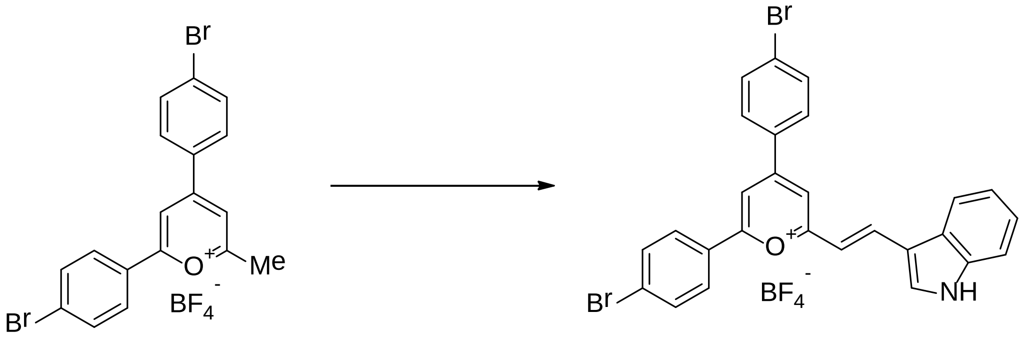

6jc47 **6jc53-3**

To pyrylium salt **6jc47** (169 mg, 0.34 mmol) and indole-3-carboxaldehyde (50 mg, 0.34 mmol) in MeOH (8 mL) was stirred at room temperature overnight. The solvent was removed and the residue was suspended in ether and filtered to give blue solid. Recrystallization from EtOH gave purple solid of 38 mg.

δH(DMSO-*d6*, 400 MHz) 12.70 (br s, 1 H, NH), 8.79 (d, 1 H, J = 14.8, HC=), 8.64 (s, 1 H, Ar), 8.62 (s, 1 H, Ar), 8.50-8.42 (m, 3 H, Ar), 8.43-8.24 (m, 3 H, Ar), 8.04-7.92 (m, 4 H, Ar), 7.66-7.60 (m, 1 H, Ar), 7.52 (d, 1 H, J = 14.8, HC=), 7.45-7.36 (m, 2 H, Ar).

2,4-bis(4-(tert-butyl)phenyl)-6-methylpyrylium boron tetrafluoride salt **6jc56**

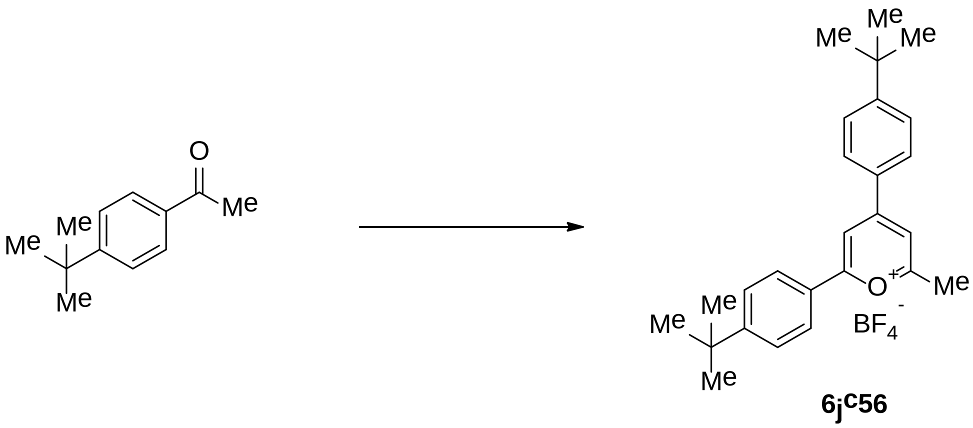

6jc56

To p-tButyl-acetophenone (2.00 g, 11.4 mmol) and acetic anhydride (1.07 mL, 11.4 mmol) was added boron trifluoride etherate (3.46 mL, 28.0 mmol) at room temperature. The reaction was heated to 135 degrees C for 2 hr, cooled, poured into EtOAc and the yellow solid filtered to give 494 mg.

δ_H(MeOH-*d*$_4$, 400 MHz) 8.86 (s, 1 H, Ar), 8.36-8.32 (m, 3 H, Ar), 8.26 (d, 2 H, J = 8.8, Ar), 7.77 (d, 4 H, J = 7.6, Ar), 3.00 (s, 3 H, Me), 1.40 (s, 18 H, 2 x tBu).

2,4-bis(3,4-dimethylphenyl)-6-methylpyrylium boron tetrafluoride salt **6jc57**

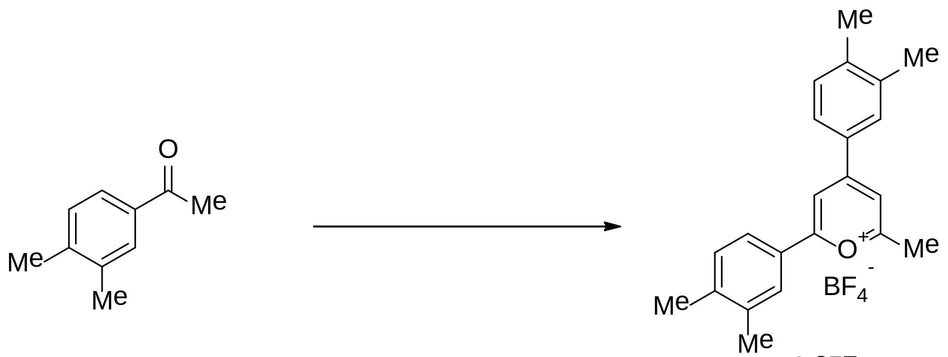

6jc57

To 3,4-dimethyl-acetophenone (2.00 g, 13.5 mmol) and acetic anhydride (1.28 mL, 13.5 mmol) was added boron trifluoride etherate (4.10 mL, 33.2 mmol) at room temperature. The reaction was heated to 135 degrees C for 3 hr, cooled, poured into EtOAc and the yellow solid filtered to give 417 mg.

δ_H(MeOH-*d*$_4$, 400 MHz) 8.82 (s, 1 H, Ar), 8.30 (s, 1 H, Ar), 8.16-8.09 (m, 2 H, Ar), 8.05 (d, 1 H, J = 7.6, Ar), 7.48 (d, 2 H, J = 8.0, Ar), 2.97 (s, 3 H, Me), 2.44 (s, 6 H, 2 x Me), 2.42 (s, 6 H, 2 x Me).

(E)-2-(2-(1,2,3,5,6,7-hexahydropyrido[3,2,1-ij]quinolin-9-yl)vinyl)-4,6-diphenylpyrylium boron tetrafluoride salt **6jc58**

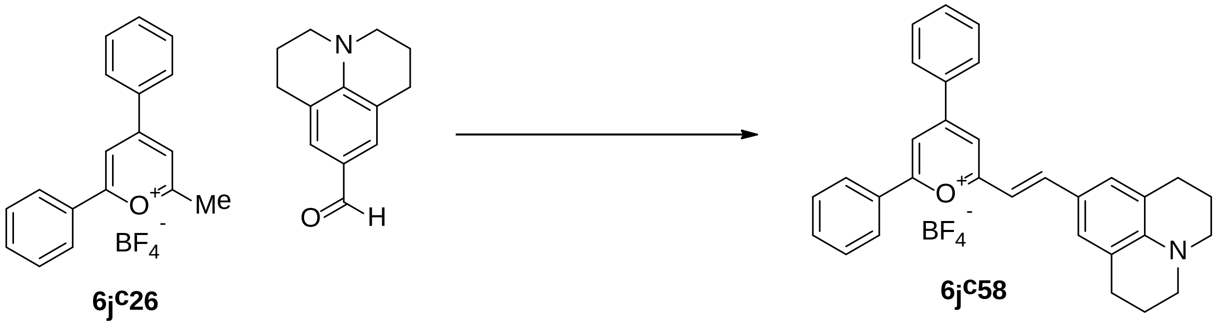

6jc26

6jc58

To pyrylium salt **6jc26** (120 mg, 0.37 mmol) and 9-CHO-julolidine (34 mg, 0.22 mmol) in MeOH (8 mL) was stirred at room temperature overnight. The solvent was removed and the residue was suspended in ether and filtered to give solid. Recrystallization from EtOH gave purple solid of 96 mg.

δ_H(DMSO-d_6, 400 MHz) 8.44-8.14 (m, 7 H, Ar, HC=), 7.75-7.60 (m, 6 H, Ar), 7.47 (s, 2 H, Ar), 7.22 (d, 1 H, J = 15.2, HC=), 3.50-3.40 (m, 4 H, 2 x CH_2), 2.78-2.70 (m, 4 H, 2 x CH_2), 2.01-1.85 (m, 4 H, 2 x CH_2).

(E)-2-(4-(dimethylamino)styryl)-4,6-bis(3,4-dimethylphenyl)pyrylium boron tetrafluoride salt **6jc59-1**

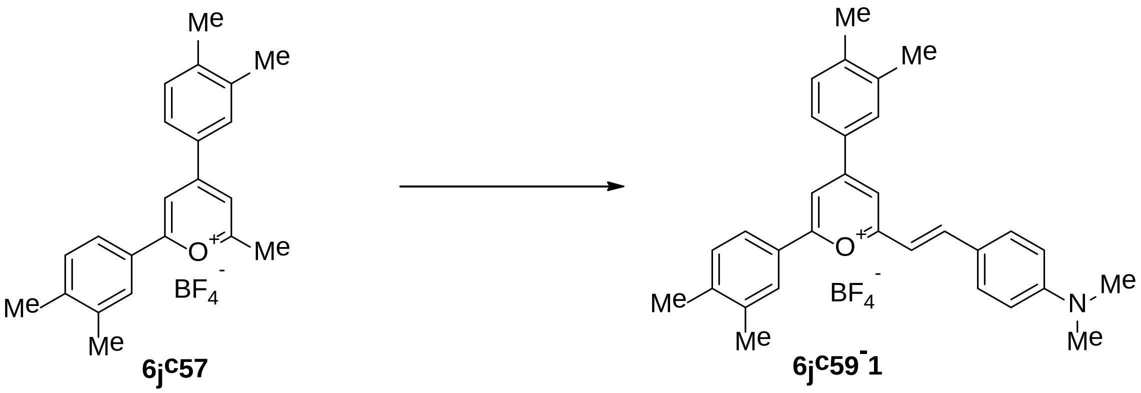

To pyrylium salt **6jc57** (100 mg, 0.22 mmol) and p-dimethylamino benzaldehyde (34 mg, 0.22 mmol) in MeOH (8 mL) was stirred at room temperature overnight. The solvent was removed and the residue was suspended in ether and filtered to give blue solid. Recrystallization from EtOH gave green solid of 79 mg.

δ_H(DMSO-d_6, 400 MHz) 8.53 (s, 1 H, Ar), 8.41 (s, 1 H, Ar), 8.36 (d, 1 H, J = 15.6, HC=), 8.26 (s, 1 H, Ar), 8.23 (d, 1 H, J = 8.8, Ar), 8.16 (s, 1 H, Ar), 8.12 (d, 1 H, J = 7.6, Ar), 7.83 (d, 2 H, J = 8.8, Ar), 7.52-7.43 (m, 2 H, Ar), 7.37 (d, 1 H, J = 15.6, HC=), 6.91 (d, 2 H, J = 8.8, Ar), 3.15 (s, 6 H, NMe_2), 2.43 (s, 3 H, Me), 2.42 (s, 3 H, Me), 2.40 (s, 6 H, 2 x Me).

2-((1E,3E)-4-(4-(dimethylamino)phenyl)buta-1,3-dien-1-yl)-4,6-bis(3,4-dimethylphenyl)pyrylium boron tetrafluoride salt **6jc59-2**

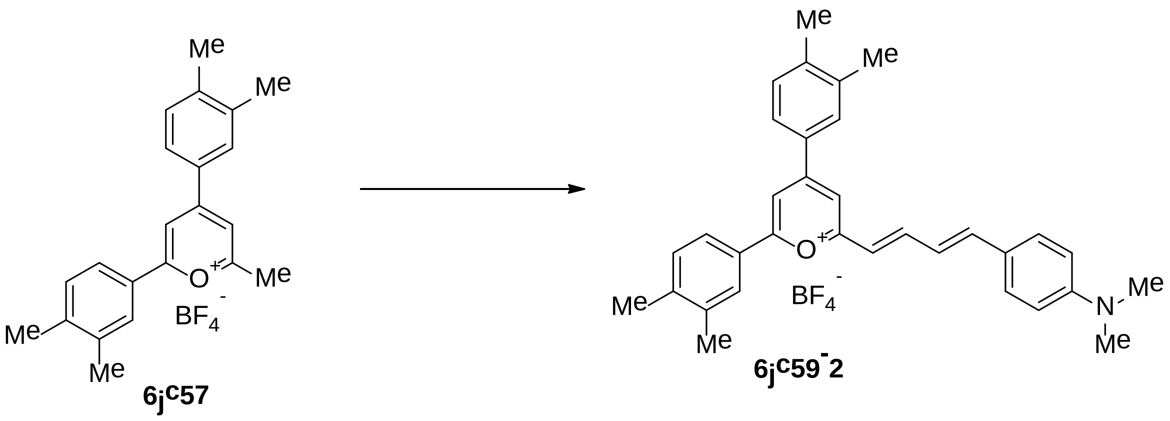

To pyrylium salt **6jc57** (100 mg, 0.22 mmol) and p-dimethylamino cinnamaldehyde 39 mg, 0.22 mmol) in MeOH (8 mL) was stirred at room temperature overnight. The solvent was removed and the residue was suspended in ether and filtered to give blue solid. Recrystallization from EtOH gave green solid of 70 mg.

δ_H(DMSO-d_6, 400 MHz) 8.58 (s, 1 H, Ar), 8.42 (s, 1 H, Ar), 8.30-8.16 (m, 4 H, Ar, HC=), 8.14 (d, 1 H, J = 8.0, Ar), 7.62 (d, 2 H, J = 8.4, Ar), 7.53-7.46 (m, 3 H, Ar, HC=), 7.25 (t, 1 H, J = 14.0, HC=), 6.90-6.77 (m, 3 H, Ar, HC=), 3.07 (s, 6 H, NMe_2), 2.44 (s, 3 H, Me), 2.41 (s, 3 H, Me), 2.40 (s, 6 H, 2 x Me).

(E)-2-(2-(1H-indol-3-yl)vinyl)-4,6-bis(3,4-dimethylphenyl)pyrylium boron tetrafluoride salt **6jc59-3**

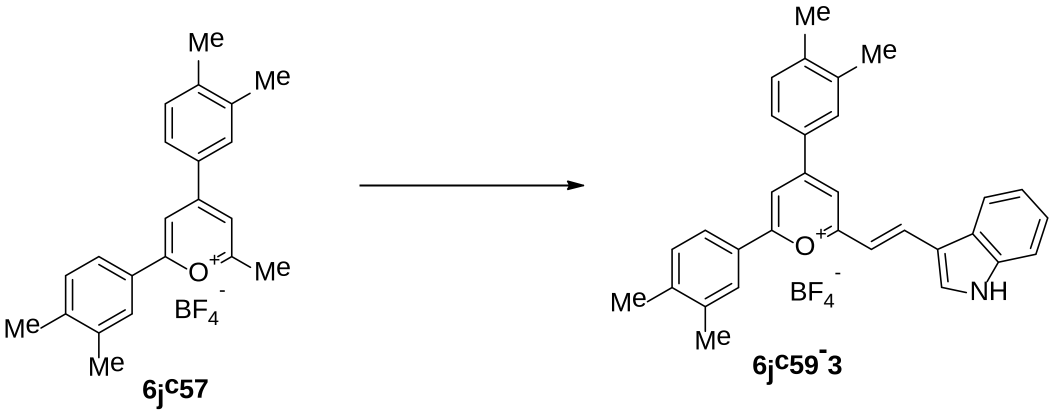

To pyrylium salt **6jc57** (100 mg, 0.22 mmol) and indole-3-carboxaldehyde (32 mg, 0.22 mmol) in MeOH (8 mL) was stirred at room temperature for 2 days. The solvent was removed and the

residue was suspended in ether and filtered to give blue solid. Recrystallization from EtOH gave green solid of 55 mg.

δ_H(DMSO-d_6, 400 MHz) 12.54 (br s, 1 H, NH), 8.64 (d, 1 H, J = 15.6, HC=), 8.55 (s, 1 H, Ar), 8.52 (s, 1 H, Ar), 8.43 (s, 1 H, Ar), 8.32-8.22 (m, 3 H, Ar), 8.16 (s, 1 H, Ar), 8.12 (d, 1 H, J = 8.4, Ar), 7.63-7.59 (m, 1 H, Ar), 7.56-7.45 (m, 3 H, Ar, HC=), 7.43-7.34 (m, 2 H, Ar), 2.45 (s, 3 H, Me), 2.43 (s, 3 H, Me), 2.41 (s, 6 H, 2 x Me).

(E)-2,4-bis(4-(tert-butyl)phenyl)-6-(4-(dimethylamino)styryl)pyrylium boron tetrafluoride salt **6jc60-1**

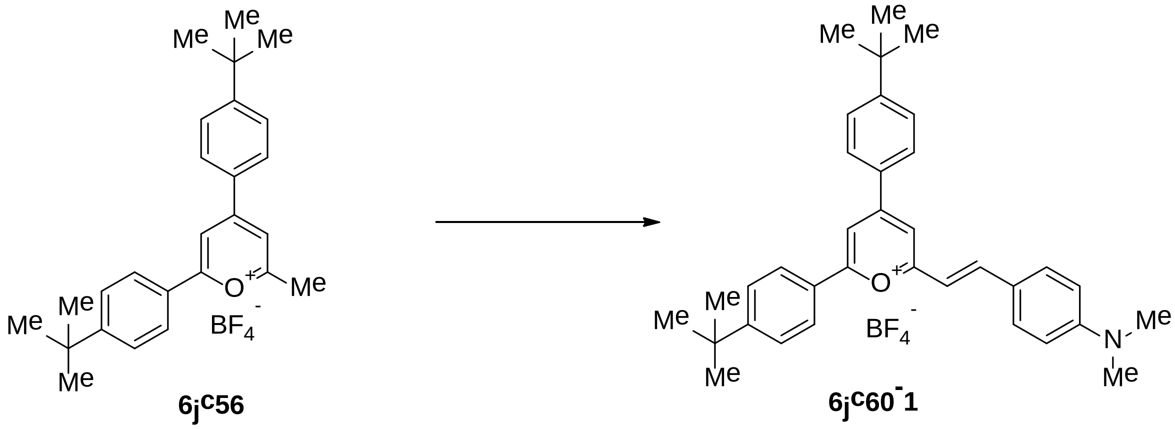

To pyrylium salt **6jc56** (100 mg, 0.22 mmol) and p-dimethylamino benzaldehyde (40 mg, 0.22 mmol) in MeOH (8 mL) was stirred at room temperature overnight. The solvent was removed and the residue was suspended in ether and filtered to give solid. Recrystallization from EtOH gave green solid of 67 mg.

δ_H(DMSO-d_6, 400 MHz) 8.56 (s, 1 H, Ar), 8.41 (s, 1 H, Ar), 8.40-8.33 (m, 3 H, Ar, HC=), 8.26 (d, 2 H, J = 8.4, Ar), 7.83 (d, 2 H, J = 9.6, Ar), 7.78-7.71 (m, 4 H, Ar), 7.37 (d, 1 H, J = 15.6, HC=), 6.91 (d, 2 H, J = 8.8, Ar), 3.15 (s, 6 H, NMe_2), 1.39 (s, 9 H, tBu), 1.38 (s, 9 H, tBu).

2,4-bis(4-(tert-butyl)phenyl)-6-((1E,3E)-4-(4-(dimethylamino)phenyl)buta-1,3-dien-1-yl)pyrylium boron tetrafluoride salt **6jc60-2**

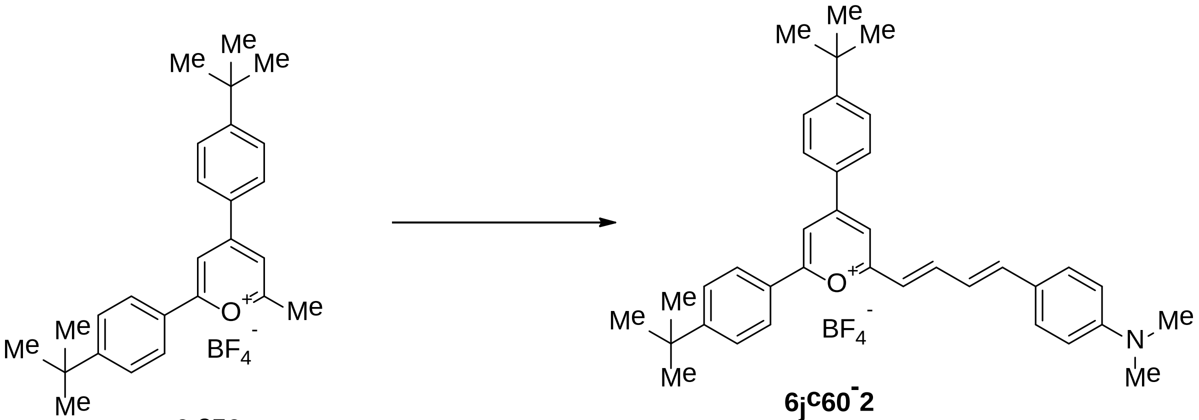

6jc56 6jc60-2

To pyrylium salt **6jc56** (100 mg, 0.22 mmol) and p-dimethylamino cinnamaldehyde (44 mg, 0.22 mmol) in MeOH (8 mL) was stirred at room temperature overnight. The solvent was removed and the residue was suspended in ether and filtered to give solid. Recrystallization from EtOH gave green solid of 50 mg.

δ_H(DMSO-*d*$_6$, 400 MHz) 8.61 (s, 1 H, Ar), 8.42-8.37 (m, 3 H, Ar), 8.29 (d, 2 H, J = 8.8, Ar), 8.22 (t, 1 H, J = 14.8, HC=), 7.54 (d, 4 H, J = 8.0, Ar), 7.62 (d, 2 H, J = 9.2, Ar), 7.52 (d, 1 H, J = 14.8, HC=), 7.27 (t, 1 H, J = 15.2, HC=), 6.86 (d, 1 H, J = 15.2, HC=), 6.81 (d, 2 H, J = 8.8, Ar), 3.07 (s, 6 H, NMe_2), 1.39 (s, 9 H, tBu), 1.38 (s, 9 H, tBu).

(E)-2-(2-(1H-indol-3-yl)vinyl)-4,6-bis(4-(tert-butyl)phenyl)pyrylium boron tetrafluoride salt **6jc60-3**

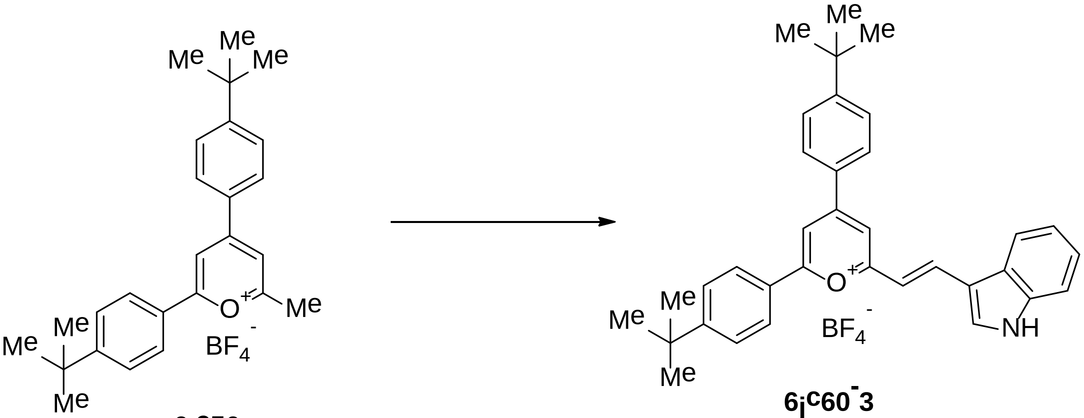

6jc56 6jc60-3

To pyrylium salt **6jc56** (100 mg, 0.22 mmol) and indole-3-carboxaldehyde (32 mg, 0.22 mmol) in MeOH (8 mL) was stirred at room temperature overnight. The solvent was removed and the

residue was suspended in ether and filtered to give solid. Recrystallization from EtOH gave green solid of 50 mg.

δ_H(DMSO-d_6, 400 MHz) 12.56 (br s, 1 H, NH), 8.67 (d, 1 H, J = 15.6, HC=), 8.58 (s, 1 H, Ar), 8.52 (s, 1 H, Ar), 8.46-8.40 (m, 3 H, Ar), 8.32-8.24 (m, 3 H, Ar), 7.80-7.72 (m, 4 H, Ar), 7.66-7.59 (m, 1 H, Ar), 7.51 (d, 1 H, J = 15.6, HC=), 7.43-7.35 (m, 2 H, Ar), 1.39 (s, 18 H, 2 x tBu).

2,4-bis(4-ethylphenyl)-6-methylpyrylium boron tetrafluoride salt **6jc61**

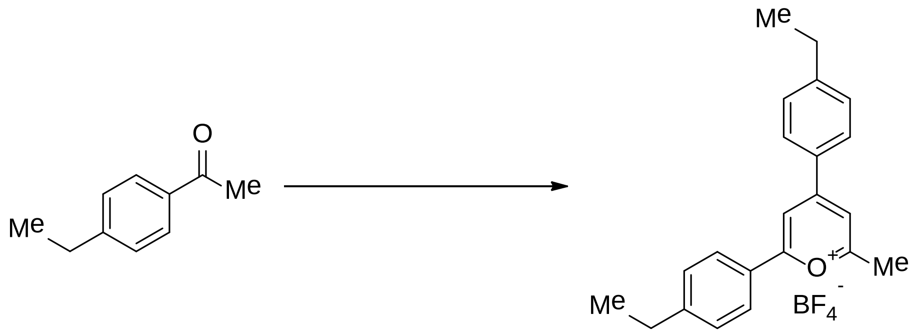

6jc61

To p-ethyl-acetophenone (2.00 g, 13.51 mmol) and acetic anhydride (1.28 mL, 13.51 mmol) was added boron trifluoride etherate (4.17 mL, 33.8 mmol) at room temperature. The reaction was heated to 135 degrees C for 3 hr, cooled, poured into EtOAc and the yellow solid filtered to give 214 mg.

δ_H(MeOH-d_4, 400 MHz) 8.85 (s, 1 H, Ar), 8.35-8.30 (m, 3 H, Ar), 8.24 (d, 2 H, J = 8.4, Ar), 7.57 (d, 4 H, J = 8.0, Ar), 2.99 (s, 3 H, Me), 2.81 (q, 4 H, J = 8.0, 2 x CH_2), 1.30 (t, 6 H, J = 8.0, 2 x CH_3).

(E)-2-(4-(dimethylamino)styryl)-4,6-bis(4-ethylphenyl)pyrylium boron tetrafluoride salt **6jc64-1**

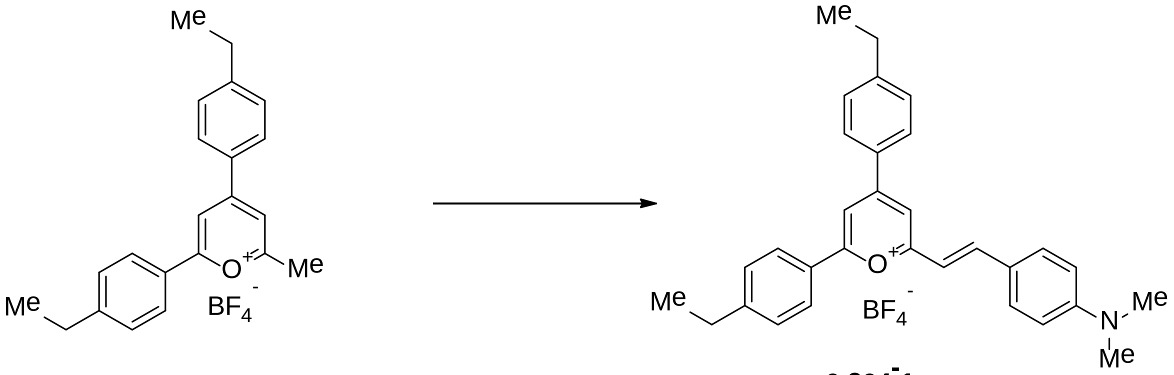

6jc61 **6jc64-1**

To pyrylium salt **6jc61** (50 mg, 0.13 mmol) and p-dimethylamino benzaldehyde (20 mg, 0.13 mmol) in MeOH (8 mL) was stirred at room temperature overnight. The solvent was removed and the residue was suspended in ether and filtered to give blue solid. Recrystallization from EtOH gave brown solid of 41 mg.

δ_H(DMSO-d_6, 400 MHz) 8.56 (s, 1 H, Ar), 8.46-8.33 (m, 4 H, Ar, HC=), 8.29 (d, 2 H, J = 8.0, Ar), 7.84 (d, 2 H, J = 8.4, Ar), 7.62-7.54 (m, 4 H, Ar), 7.38 (d, 1 H, J = 16.4, HC=), 6.92 (d, 2 H, J = 8.4, Ar), 3.15 (s, 6 H, NMe_2), 2.79 (q, 4 H, J = 7.2, 2 x CH_2), 1.28 (t, 6 H, J = 7.2, 2 x CH_3).

2-((1E,3E)-4-(4-(dimethylamino)phenyl)buta-1,3-dien-1-yl)-4,6-bis(4-ethylphenyl)pyrylium boron tetrafluoride salt **6jc64-2**

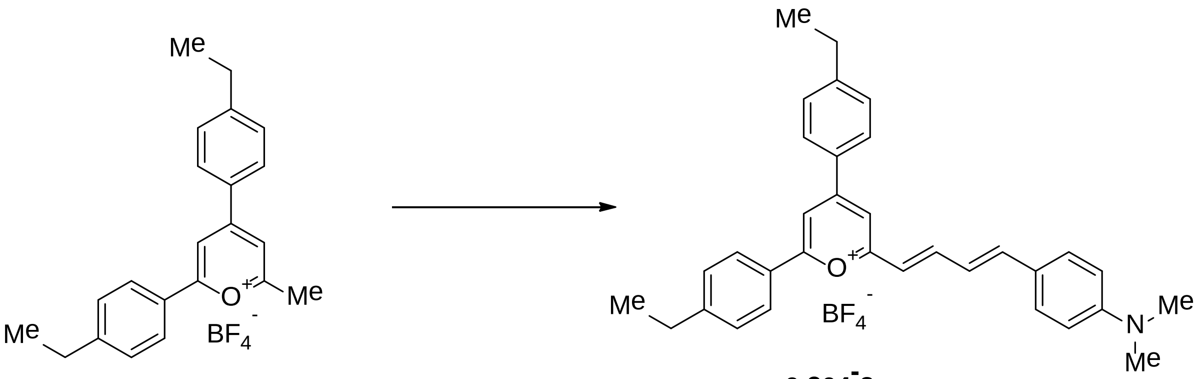

6jc61 **6jc64-2**

To pyrylium salt **6jc61** (50 mg, 0.13 mmol) and p-dimethylamino cinnamaldehyde (23 mg, 0.13 mmol) in MeOH (8 mL) was stirred at room temperature overnight. The solvent was removed and the residue was suspended in ether and filtered to give blue solid. Recrystallization from EtOH gave brown solid of 31 mg.

δ_H(DMSO-d_6, 400 MHz) 8.63 (s, 1 H, Ar), 8.43 (s, 1 H, Ar), 8.40 (d, 2 H, J = 7.6, Ar), 8.32 (d, 2 H, J = 7.6, Ar), 8.24 (t, 1 H, J = 14.0, HC=), 7.67-7.56 (m, 6 H, Ar), 7.52 (d, 1 H, J = 14.8, HC=), 7.27 (t, 1 H, J = 14.8, HC=), 6.86 (d, 1 H, J = 16.0, HC=), 6.81 (d, 2 H, J = 8.8, Ar), 3.08 (s, 6 H, NMe_2), 2.79 (q, 4 H, J = 7.2, 2 x CH_2), 1.27 (t, 6 H, J = 7.2, 2 x CH_3).

(E)-2-(2-(1H-indol-3-yl)vinyl)-4,6-bis(4-ethylphenyl)pyrylium boron tetrafluoride salt **6jc64-3**

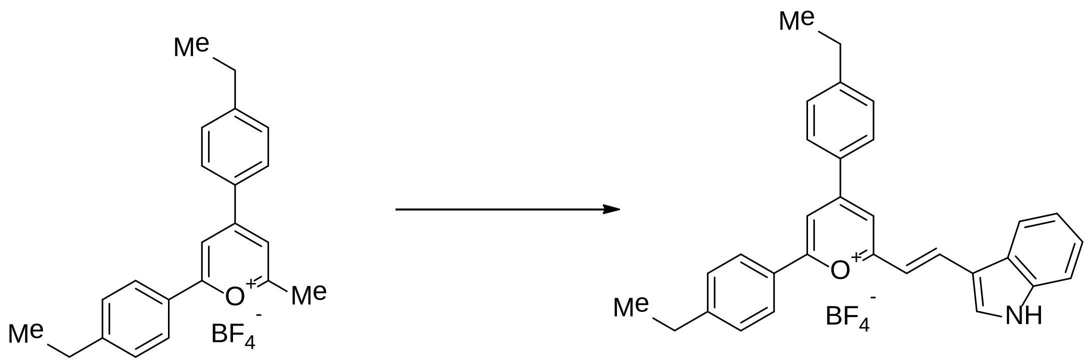

6jc61 6jc64-3

To pyrylium salt **6jc61** (50 mg, 0.13 mmol) and indole-3-carboxaldehyde (19 mg, 0.13 mmol) in MeOH (8 mL) was stirred at room temperature for 2 days. The solvent was removed and the residue was suspended in ether and filtered to give blue solid. Recrystallization from EtOH gave brown solid of 24 mg.

δ_H(DMSO-d_6, 400 MHz) 12.56 (br s, 1 H, NH), 8.68 (d, 1 H, J = 15.6, HC=), 8.59 (s, 1 H, Ar), 8.54 (s, 1 H, Ar), 8.47-8.41 (m, 3 H, Ar), 8.34-8.24 (m, 3 H, Ar), 7.66-7.57 (m, 5 H, Ar), 7.51 (d, 1 H, J = 15.6, HC=), 7.43-7.37 (m, 2 H, Ar), 2.80 (q, 4 H, J = 8.0, 2 x CH_2), 1.28 (t, 6 H, J = 8.0, 2 x CH_3).

(E)-2-(2-(1,2,3,5,6,7-hexahydropyrido[3,2,1-ij]quinolin-9-yl)vinyl)-4,6-di-p-tolylpyrylium boron tetrafluoride salt **6jc65-1**

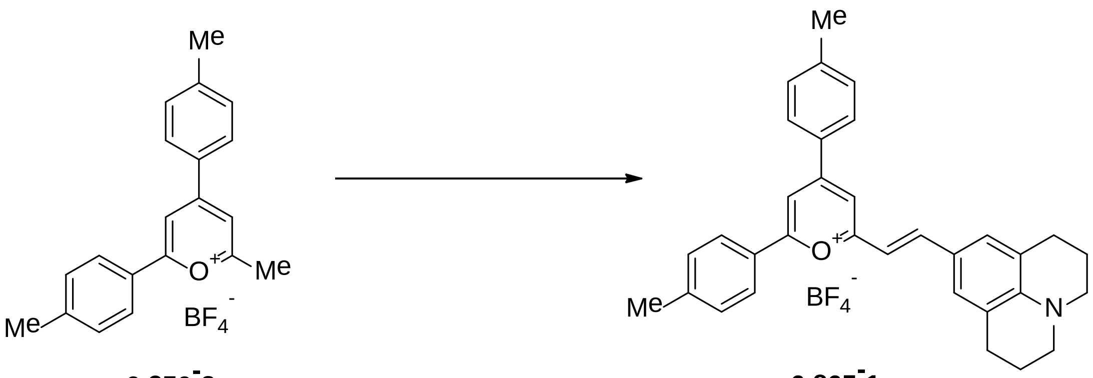

6jc50-2 6jc65-1

To pyrylium salt **6jc50-2** (50 mg, 0.14 mmol) and 9-CHO-julolidine (28 mg, 0.14 mmol) in MeOH (8 mL) was stirred at room temperature overnight. The solvent was removed and the residue was suspended in ether and filtered to give solid. Recrystallization from EtOH gave red solid of 48 mg.

δH(DMSO-*d6*, 400 MHz) 8.37-8.27 (m, 3 H, Ar, HC=), 8.21-8.15 (m, 4 H, Ar), 7.54-7.46 (m, 4 H, Ar), 7.41 (s, 2 H, Ar), 7.17 (d, 1 H, J = 15.6, HC=), 3.43-3.3.38 (m, 4 H, 2 x CH_2), 2.79-2.70 (m, 4 H, 2 x CH_2), 1.96-1.86 (m, 4 H, 2 x CH_2).

(E)-2-(2-(1-methyl-1H-indol-3-yl)vinyl)-4,6-di-p-tolylpyrylium boron tetrafluoride salt **6jc65-2**

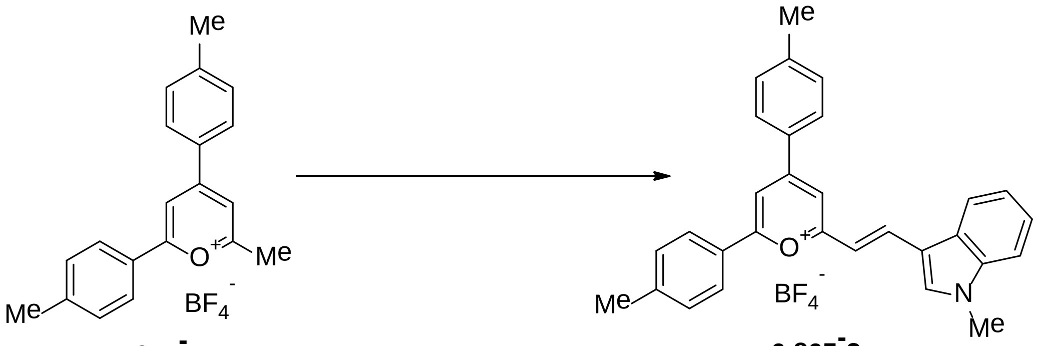

6jc50-2 **6jc65-2**

To pyrylium salt **6jc50-2** (50 mg, 0.14 mmol) and N-methyl-indole-3-carboxaldehyde (23 mg, 0.14 mmol) in MeOH (8 mL) was stirred at room temperature for 2 days. The solvent was removed and the residue was suspended in ether and filtered to give blue solid. Recrystallization from EtOH gave purple solid of 40 mg.

δH(DMSO-*d6*, 400 MHz) 8.64 (d, 1 H, J = 15.6, HC=), 8.57 (s, 1 H, Ar), 8.53 (s, 1 H, Ar), 8.45-8.37 (m, 3 H, Ar), 8.32-8.23 (m, 3 H, Ar), 7.70 (d, 1 H, J = 6.8, Ar), 7.56 (t, 4 H, J = 6.8, Ar), (m, 5 H, Ar), 7.50-7.40 (m, 3 H, Ar, HC=), 3.97 (s, 3 H, NMe), 2.50 (s, 6 H, 2 x CH_3).

(E)-2-(2-(1-methyl-1H-indol-3-yl)vinyl)-4,6-diphenylpyrylium boron tetrafluoride salt **6jc66-1**

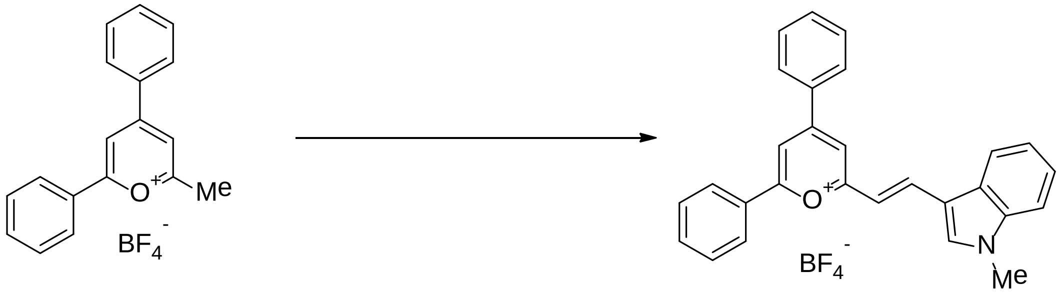

6jc66-1

To pyrylium salt **6jc26** (53 mg, 0.16 mmol) and N-methyl-indole-3-carboxaldehyde (25 mg, 0.16 mmol) in MeOH (8 mL) was stirred at room temperature for 2 days. The solvent was removed and the residue was suspended in ether and filtered to give blue solid. Recrystallization from EtOH gave purple solid of 34 mg.

δH(DMSO-*d6*, 400 MHz) 8.72 (d, 1 H, J = 15.6, HC=), 8.63 (s, 1 H, Ar), 8.59 (s, 1 H, Ar), 8.53 (d, 2 H, J = 7.6, Ar), 8.45 (s, 1 H, Ar), 8.38-8.29 (m, 3 H, Ar), 7.84-7.70 (m, 7 H, Ar), 7.52 (d, 1 H, J = 15.6, HC=), 7.49-7.43 (m, 2 H, Ar), 3.99 (s, 3 H, NMe).

(E)-2,4-bis(4-ethylphenyl)-6-(2-(1-methyl-1H-indol-3-yl)vinyl)pyrylium boron tetrafluoride salt **6jc66-2**

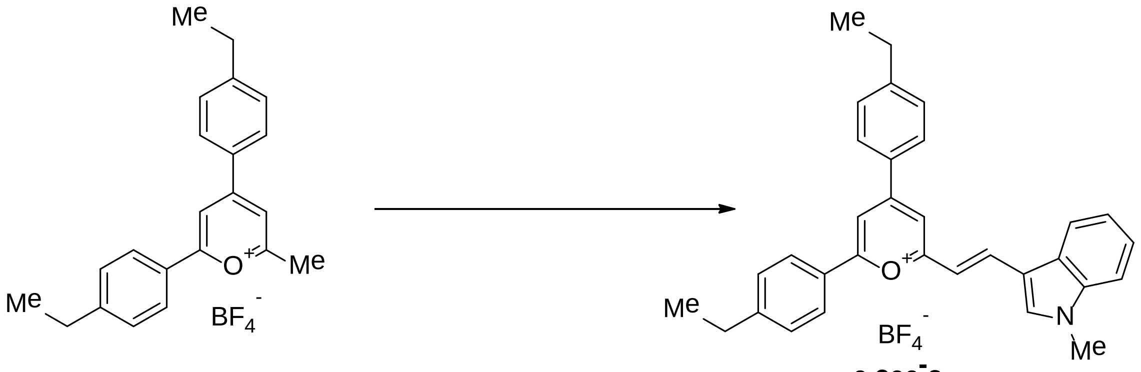

6jc66-2

To pyrylium salt **6jc61** (62 mg, 0. 16 mmol) and N-methyl-indole-3-carboxaldehyde (25 mg, 0. 16 mmol) in MeOH (8 mL) was stirred at room temperature for 2 days. The solvent was removed and the residue was suspended in ether and filtered to give blue solid. Recrystallization from EtOH gave purple solid of 13 mg.

δH(DMSO-*d6*, 400 MHz) 8.65 (d, 1 H, J = 15.6, HC=), 8.59 (s, 1 H, Ar), 8.54 (s, 1 H, Ar), 8.45 (d, 2 H, J = 7.6, Ar), 8.41 (s, 1 H, Ar), 8.34-8.26 (m, 3 H, Ar), 7.74-7.68 (m, 1 H, Ar), 7.64-7.56 (m, 4 H, Ar), 7.52-7.42 (m, 3 H, Ar), 3.98 (s, 3 H, NMe), 3.00-2.75 (m, 4 H, 2 x CH_2), 1.29 (t, 6 H, J = 7.2, 2 x CH_3).

(E)-2,4-bis(4-(tert-butyl)phenyl)-6-(2-(1-methyl-1H-indol-3-yl)vinyl)pyrylium boron tetrafluoride salt **6jc66-3**

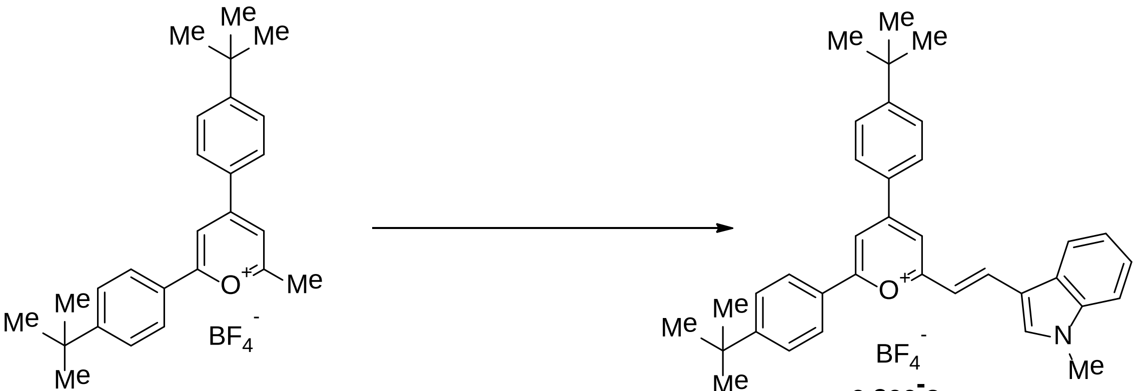

6jc66-3

To pyrylium salt **6jc56** (71 mg, 0.16 mmol) and N-methyl-indole-3-carboxaldehyde (25 mg, 0.16 mmol) in MeOH (8 mL) was stirred at room temperature for 2 days. The solvent was removed and the residue was suspended in ether and filtered to give blue solid. Recrystallization from EtOH gave purple solid of 27 mg.

δ_H(DMSO-d_6, 400 MHz) 8.64 (d, 1 H, J = 15.6, HC=), 8.58 (s, 1 H, Ar), 8.52 (s, 1 H, Ar), 8.47-8.40 (m, 3 H, Ar), 8.32-8.24 (m, 3 H, Ar), 7.81-7.68 (m, 5 H, Ar), 7.53-7.42 (m, 3 H, Ar, HC=), 3.99 (s, 3 H, NMe), 1.40 (s, 18 H, 6 x CH_3).

(E)-2,4-bis(3,4-dimethylphenyl)-6-(2-(1-methyl-1H-indol-3-yl)vinyl)pyrylium boron tetrafluoride salt **6jc66-4**

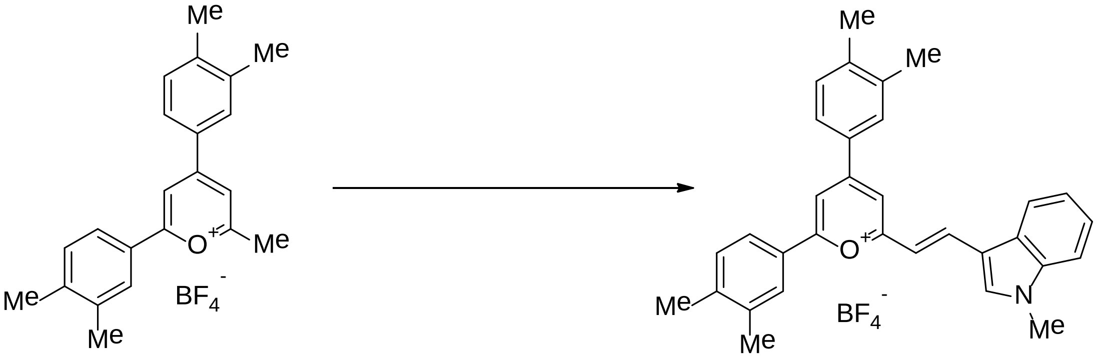

6jc66-4

To pyrylium salt **6jc57** (67 mg, 0.16 mmol) and N-methyl-indole-3-carboxaldehyde (25 mg, 0.16 mmol) in MeOH (8 mL) was stirred at room temperature for 2 days. The solvent was removed and the residue was suspended in ether and filtered to give blue solid. Recrystallization from EtOH gave green solid of 38 mg.

δH(DMSO-*d6*, 400 MHz) 8.61 (d, 1 H, J = 16.0, HC=), 8.54 (s, 1 H, Ar), 8.51 (s, 1 H, Ar), 8.40 (s, 1 H, Ar), 8.34-8.8.22 (m, 3 H, Ar), 8.19-8.8.06 (m, 2 H, Ar), 7.70 (d, 1 H, J = 6.8, Ar), 7.58-7.40 (m, 5 H, Ar, HC=), 3.97 (s, 3 H, NMe), 2.45 (s, 3 H, CH_3), 2.43 (s, 3 H, CH_3), 2.41 (s, 6 H, 2 x CH_3).

2,4-diphenyl-6-((1E,3E)-3-(1,3,3-trimethylindolin-2-ylidene)prop-1-en-1-yl)pyrylium boron tetrafluoride salt **6jc67**

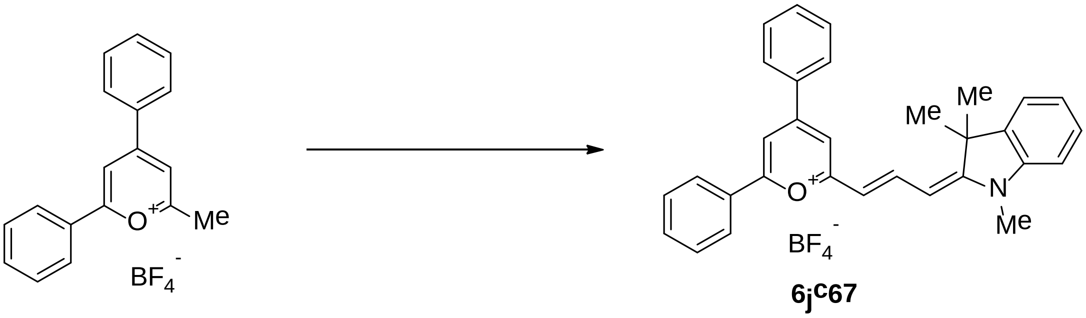

6jc67

To pyrylium salt **6jc26** (50 mg, 0.14 mmol) and 2-(1,3,3-Trimethylindolin-2-ylidene)acetaldehyde (28 mg, 0.14 mmol) in acetic anhydride (2 mL) was stirred at reflux for 1 hr. The reaction was cooled and co- evaporated with toluene (x 3), washed with ether and decanted (x 2). The residue was purified by column chromatography DCM/MeOH and transferred to a vial to give a blue solid 69 mg.

δH(DMSO-*d6*, 400 MHz) 8.47 (t, 1 H, J = 13.2, HC=), 8.26-8.10 (m, 4 H, Ar), 7.95 (s, 1 H, Ar), 7.90-7.59 (m, 8 H, Ar), 7.53-7.44 (m, 2 H, Ar), 7.35 (t, 1 H, J = 7.6, Ar), 6.58 (d, 1 H, J = 13.2, HC=), 6.39 (br s, 1 H, HC=), 3.73 (s, 3 H, NMe), 1.74 (s, 6 H, 2 x CH_3).

2,4-di-p-tolyl-6-((1E,3E)-3-(1,3,3-trimethylindolin-2-ylidene)prop-1-en-1-yl)pyrylium boron tetrafluoride salt **6jc67-A**

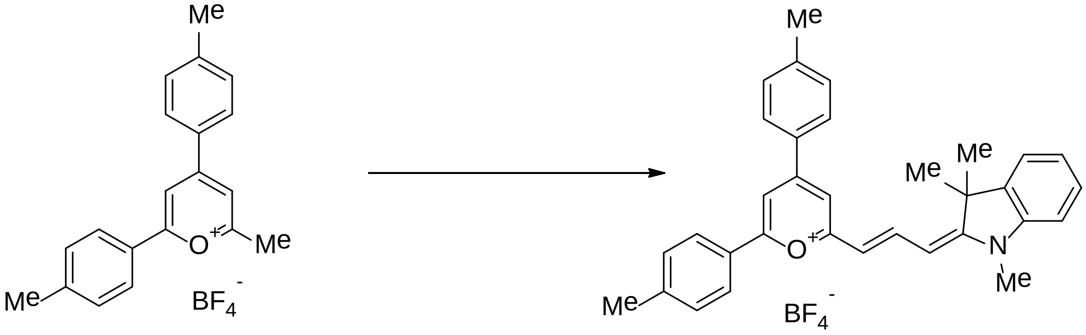

6jc67-A

To pyrylium salt **6jc50-2** (50 mg, 0.14 mmol) and 2-(1,3,3-Trimethylindolin-2-ylidene)acetaldehyde (28 mg, 0.14 mmol) in acetic anhydride (2 mL) was stirred at reflux for 30 mins. The reaction was cooled and co evaporated with toluene (x 3), washed with ether and decanted (x 2). The blue solid was transferred to a vial to give 71 mg.

δ_H(DMSO-d_6, 400 MHz) 8.41 (t, 1 H, J = 14.0, HC=), 8.09 (d, 1 H, J = 8.0, Ar), 8.05 (d, 1 H, J = 8.0, Ar), 7.91 (s, 1 H, Ar), 7.88-7.72 (m, 1 H, Ar), 7.62 (d, 1 H, J = 8.0, Ar), 7.51-7.37 (m, 6 H, Ar), 7.30-7.24 (m, 1 H, Ar), 6.45 (d, 1 H, J = 14.0, HC=), 6.35 (br s, 1 H, HC=), 3.65 (s, 3 H, NMe), 2.43 (s, 3 H, $ArCH_3$), 2.40 (s, 3 H, $ArCH_3$), 1.69 (s, 6 H, 2 x CH_3).

(E)-2,4-bis(4-chlorophenyl)-6-(2-(1-methyl-1H-indol-3-yl)vinyl)pyrylium boron tetrafluoride salt **6jc68-1**

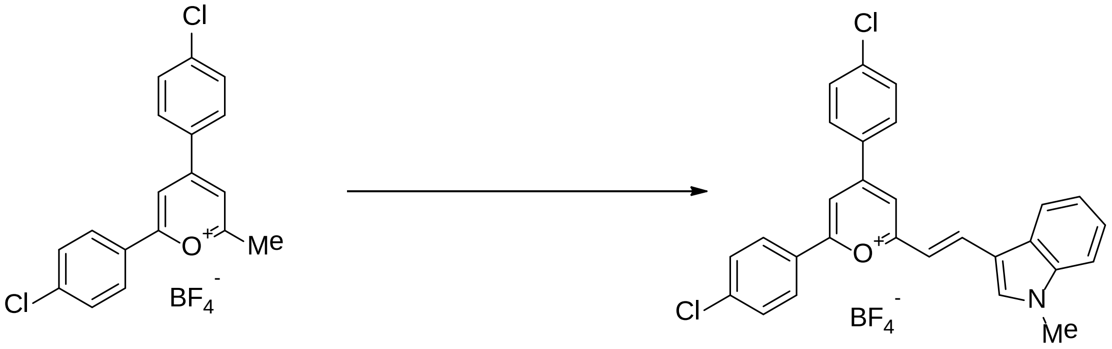

6jc68-1

To pyrylium salt **6jc32-1** (52 mg, 0.14 mmol) and N-methyl-indole-3-carboxaldehyde (23 mg, 0.14 mmol) in MeOH (8 mL) was stirred at room temperature for 2 days. The solvent was removed and the residue was suspended in ether and filtered to give blue solid. Recrystallization from EtOH gave purple solid of 27 mg.

δ_H(DMSO-d_6, 400 MHz) 8.75 (d, 1 H, J = 16.0, HC=), 8.63 (s, 1 H, Ar), 8.60 (s, 1 H, Ar), 8.54 (d, 2 H, J = 8.4, Ar), 8.45 (s, 1 H, Ar), 8.40-8.28 (m, 3 H, Ar), 7.88-7.80 (m, 4 H, Ar), 7.76-7.01 (m, 1 H, Ar), 7.55-7.43 (m, 3 H, Ar, HC=), 4.00 (s, 3 H, NMe).

(E)-2,4-bis(4-bromophenyl)-6-(2-(1-methyl-1H-indol-3-yl)vinyl)pyrylium boron tetrafluoride salt **6jc68-2**

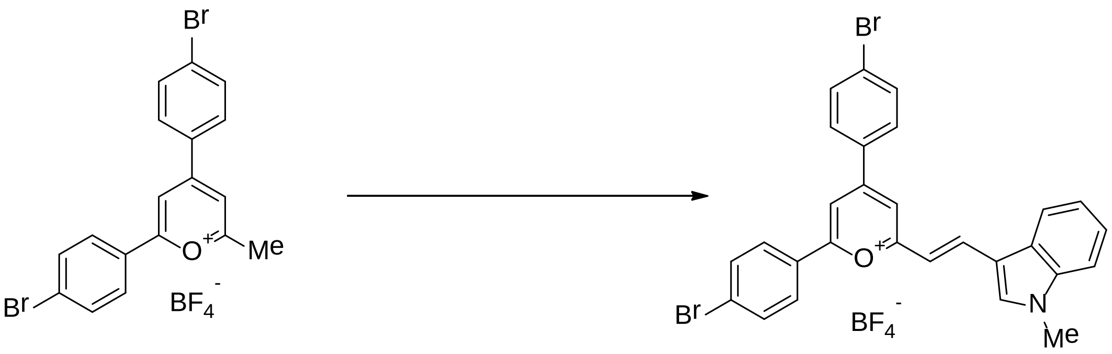

6jc68-2

To pyrylium salt **6jc47** (63 mg, 0.14 mmol) and N-methyl-indole-3-carboxaldehyde (23 mg, 0.14 mmol) in MeOH (8 mL) was stirred at room temperature for 2 days. The solvent was removed and the residue was suspended in ether and filtered to give blue solid. Recrystallization from EtOH gave purple solid of 20 mg.

δ_H(DMSO-*d*$_6$, 400 MHz) 8.74 (d, 1 H, J = 16.0, HC=), 8.62 (s, 1 H, Ar), 8.61 (s, 1 H, Ar), 8.48-8.42 (m, 3 H, Ar), 8.32-8.23 (m, 3 H, Ar), 8.06-7.92 (m, 4 H, Ar), 7.76-7.00 (m, 1 H, Ar), 7.54-7.42 (m, 3 H, Ar, HC=), 4.00 (s, 3 H, NMe).

(E)-2,4-bis(4-ethylphenyl)-6-(2-(1,2,3,5,6,7-hexahydropyrido[3,2,1-ij]quinolin-9-yl)vinyl)pyrylium boron tetrafluoride salt **6jc69-1**

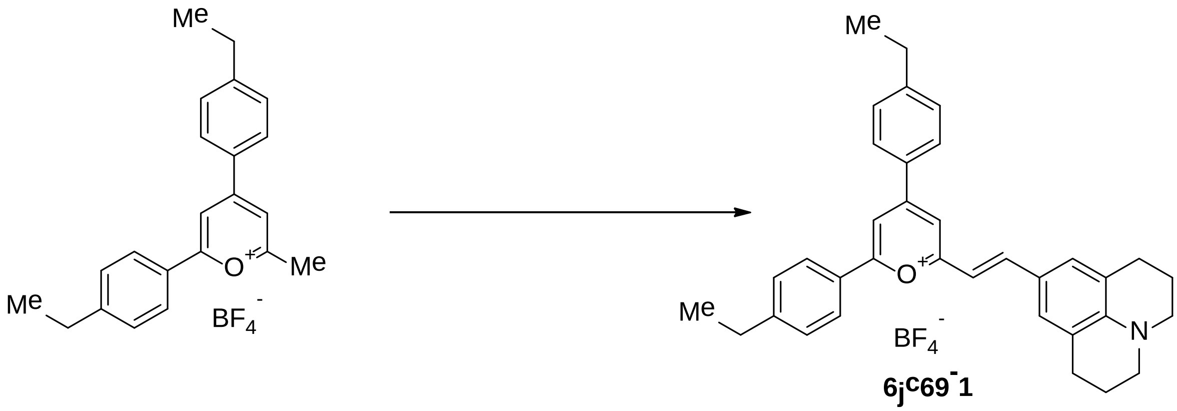

6jc69-1

To pyrylium salt **6jc61** (46 mg, 0.12 mmol) and 9-CHO-julolidine (25 mg, 0.12 mmol) in MeOH (8 mL) was stirred at room temperature overnight. The solvent was removed and the residue was suspended in ether and filtered to give solid. Recrystallization from EtOH gave purple solid of 44 mg.

δH(DMSO-*d*6, 400 MHz) 8.40-8.32 (m, 3 H, Ar, HC=), 8.27-8.18 (m, 4 H, Ar), 7.60-7.52 (m, 4 H, Ar), 7.45 (s, 2 H, Ar), 7.21 (d, 1 H, J = 15.6, HC=), 3.50-3.40 (m, 4 H, 2 x CH_2), 2.83-2.70 (m, 8 H, 4 x CH_2), 1.99-1.87 (m, 4 H, 2 x CH_2), 1.27 (t, 6 H, J = 7.2, 2 x CH_3).

(E)-2,4-bis(4-(tert-butyl)phenyl)-6-(2-(1,2,3,5,6,7-hexahydropyrido[3,2,1-ij]quinolin-9-yl)vinyl)pyrylium boron tetrafluoride salt **6jc69-2**

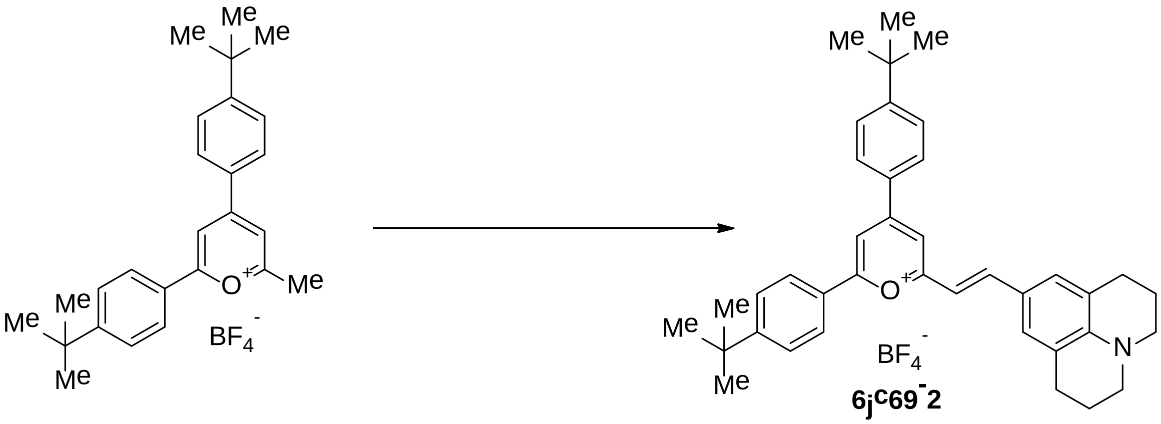

To pyrylium salt **6jc56** (53 mg, 0.12 mmol) and 9-CHO-julolidine (25 mg, 0.12 mmol) in MeOH (8 mL) was stirred at room temperature overnight. The solvent was removed and the residue was suspended in ether and filtered to give solid. Recrystallization from EtOH gave purple solid of 74 mg.

δH(DMSO-*d*6, 400 MHz) 8.38-8.31 (m, 3 H, Ar, HC=), 8.24-8.15 (m, 4 H, Ar), 7.78-7.67 (m, 4 H, Ar), 7.45 (s, 2 H, Ar), 7.19 (d, 1 H, J = 15.6, HC=), 3.49-3.40 (m, 4 H, 2 x CH_2), 2.80-2.72 (m, 4 H, 2 x CH_2), 1.97-1.88 (m, 4 H, 2 x CH_2), 1.38 (s, 18 H, 9 x CH_3).

(E)-2,4-bis(3,4-dimethylphenyl)-6-(2-(1,2,3,5,6,7-hexahydropyrido[3,2,1-ij]quinolin-9-yl)vinyl)pyrylium boron tetrafluoride salt **6jc69-3**

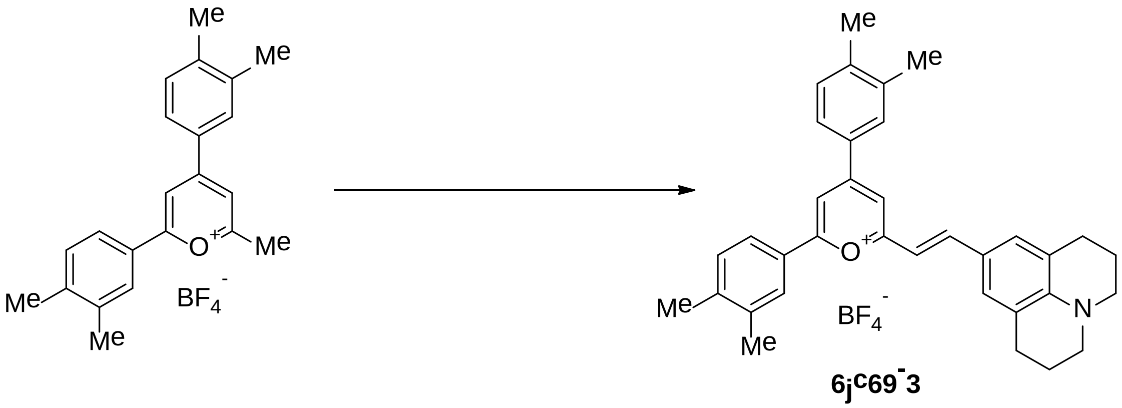

To pyrylium salt **6jc57** (50 mg, 0.12 mmol) and 9-CHO-julolidine (25 mg, 0.12 mmol) in MeOH (8 mL) was stirred at room temperature overnight. The solvent was removed and the residue was suspended in ether and filtered to give solid. Recrystallization from EtOH gave green solid of 64 mg.

δ_H(DMSO-*d*$_6$, 400 MHz) 8.33 (s, 1 H, Ar), 8.25-8.10 (m, 4 H, Ar, HC=), 8.08 (s, 1 H, Ar), 8.04 (d, 1 H, J =7.6, Ar), 7.50-7.43 (m, 2 H, Ar), 7.42 (s, 2 H, Ar), 7.18 (d, 1 H, J = 16.0, HC=), 3.48-3.40 (m, 4 H, 2 x CH_2), 2.79-2.70 (m, 4 H, 2 x CH_2), 2.42 (s, 3 H, CH_3), 2.40 (s, 3 H, CH_3), 2.38 (s, 6 H, 2 x CH_3), 1.99-1.88 (m, 4 H, 2 x CH_2).

(E)-2,4-bis(4-chlorophenyl)-6-(2-(1,2,3,5,6,7-hexahydropyrido[3,2,1-ij]quinolin-9-yl)vinyl)pyrylium boron tetrafluoride salt **6jc69-4**

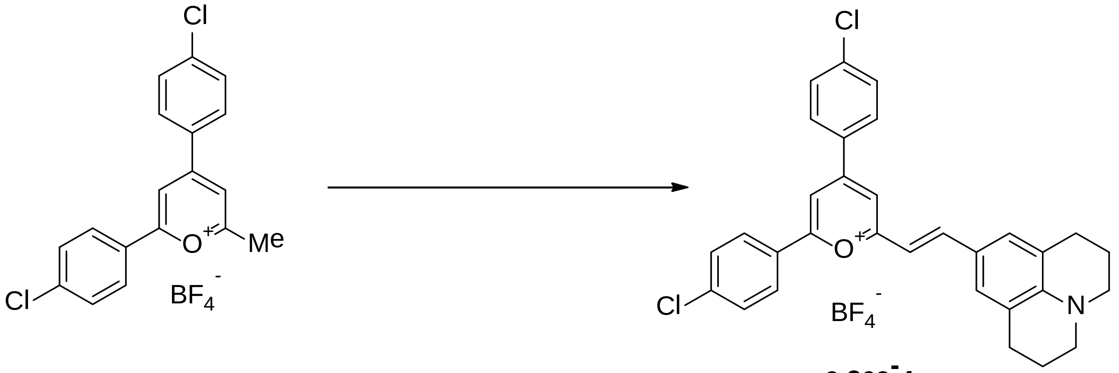

6jc69-4

To pyrylium salt **6jc32-1** (48 mg, 0.12 mmol) and 9-CHO-julolidine (25 mg, 0.12 mmol) in MeOH (8 mL) was stirred at room temperature overnight. The solvent was removed and the residue was suspended in ether and filtered to give solid. Recrystallization from EtOH gave purple solid of 39 mg.

δ_H(DMSO-*d*$_6$, 400 MHz) 8.40 (d, 2 H, J =8.4, Ar), 8.32 (s, 1 H, Ar), 8.30-8.17 (m, 4 H, Ar, HC=), 7.83-7.51 (m, 4 H, Ar), 7.45 (s, 2 H, Ar), 7.18 (d, 1 H, J = 15.2, HC=), 3.52-3.42 (m, 4 H, 2 x CH_2), 2.81-2.70 (m, 4 H, 2 x CH_2), 1.99-1.86 (m, 4 H, 2 x CH_2).

(E)-2,4-bis(4-bromophenyl)-6-(2-(1,2,3,5,6,7-hexahydropyrido[3,2,1-ij]quinolin-9-yl)vinyl)pyrylium boron tetrafluoride salt **6jc69-5**

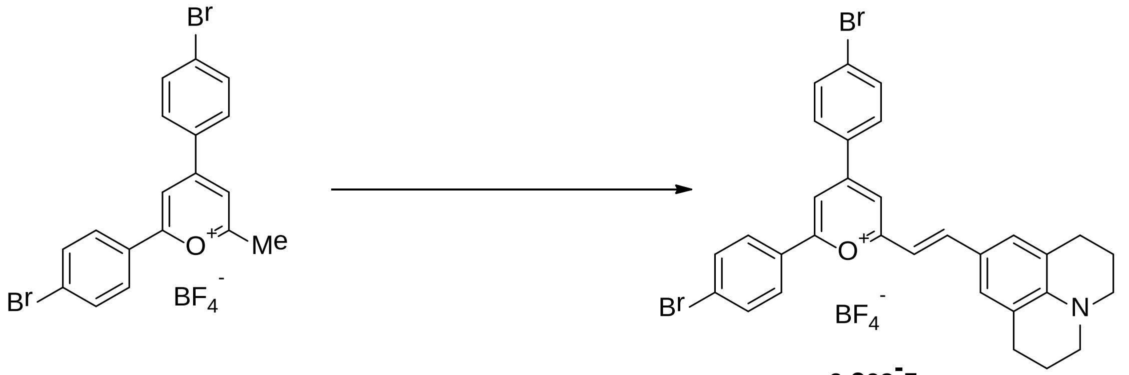

6jc69-5

To pyrylium salt **6jc47** (59 mg, 0.12 mmol) and 9-CHO-julolidine (25 mg, 0.12 mmol) in MeOH (8 mL) was stirred at room temperature overnight. The solvent was removed and the residue was suspended in ether and filtered to give solid. Recrystallization from EtOH gave green solid of 47 mg.

δ_H(DMSO-d_6, 400 MHz) 8.36-8.13 (m, 7 H, Ar, HC=), 7.94-7.85 (m, 4 H, Ar), 7.45 (s, 2 H, Ar), 7.18 (d, 1 H, J = 14.8, HC=), 3.52-3.43 (m, 4 H, 2 x CH_2), 2.82-2.71 (m, 4 H, 2 x CH_2), 1.98-1.88 (m, 4 H, 2 x CH_2).
