# Supplementary material for: Towards Development of Small Molecule Lipid II Inhibitors as Novel Antibiotics
Source: PLoS One. 2016 Oct 24;11(10):e0164515. doi: 10.1371/journal.pone.0164515 (PMC5077133; doi:10.1371/journal.pone.0164515)
Supplement: S1 Table — For each compound, chemical structure, formula and molecular weight is provided. Additionally, compounds were assayed for binding to Lipid II by SPR and tested for activity against S. aureus. (PDF) [file pone.0164515.s002.pdf]

ID and structural formula	chemical formula	MW (g/ mol)	IUPAC name	Lipid II binding	*S. aureu* s killing
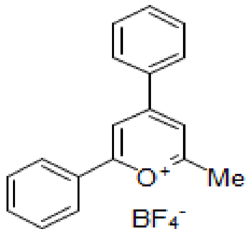 **6jc26**	$C_{18}H_{15}BF_4O$	334.12	2-methyl-4,6-diphenylpyrylium boron tetrafluoride salt	no	>64
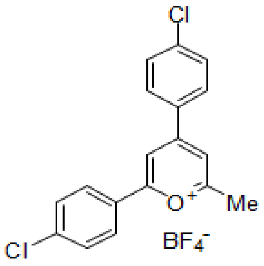 **6jc32-1**	$C_{18}H_{13}BCl_2F_4O$	403.01	2,4-bis(4-chlorophenyl)-6-methylpyrylium boron tetrafluoride salt	no	>64
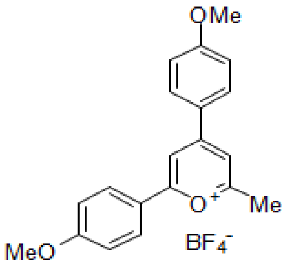 **6jc32-2**	$C_{20}H_{19}BF_4O_3$	394.17	2,4-bis(4-methoxyphenyl)-6-methylpyrylium boron tetrafluoride salt	no	>64

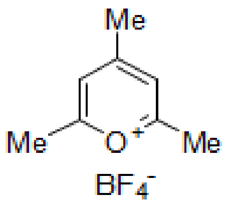 6jc36	$C_8H_{11}BF_4O$	209.98	2,4,6-trimethylpyrylium boron tetrafluoride salt	no	>64
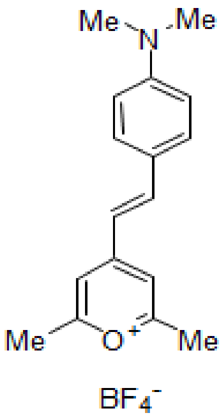 6jc37	$C_{17}H_{20}BF_4NO$	341.15	(E)-4-(4-(dimethylamino)styryl)-2,6-dimethylpyrylium boron tetrafluoride salt	no	16
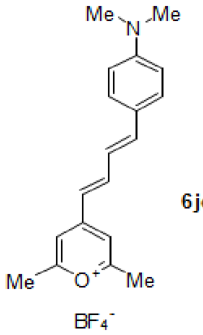 6jc38	$C_{19}H_{22}BF_4NO$	367.19	4-((1E,3E)-4-(4-(dimethylamino)phenyl)buta-1,3-dien-1-yl)-2,6-dimethylpyrylium boron tetrafluoride salt	no	32

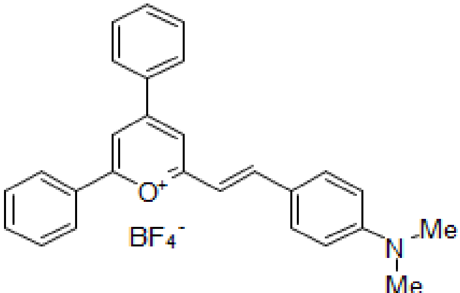 **6jc39**	$C_{27}H_{24}BF_4NO$	465.29	(E)-2-(4-(dimethylamino)styryl)-4,6-diphenylpyrylium boron tetrafluoride salt	yes	2
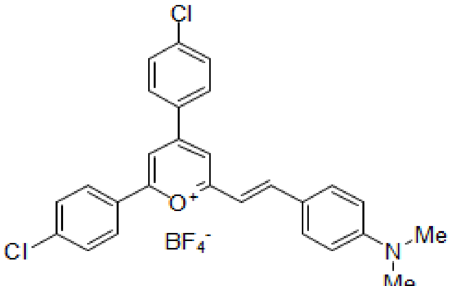 **6jc41-1**	$C_{27}H_{22}Cl_2NO^+$	534.18	(E)-2,4-bis(4-chlorophenyl)-6-(4-(dimethylamino)styryl)pyrylium boron tetrafluoride salt	yes	32
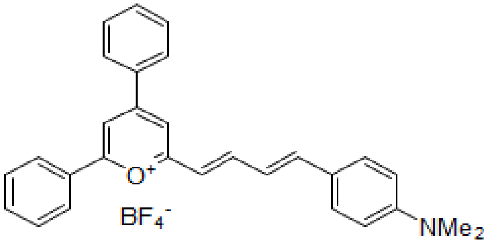 **6jc43-1**	$C_{29}H_{26}BF_4NO$	491.33	2-((1E,3E)-4-(4-(dimethylamino)phenyl)buta-1,3-dien-1-yl)-4,6-diphenylpyrylium boron tetrafluoride salt	yes	4

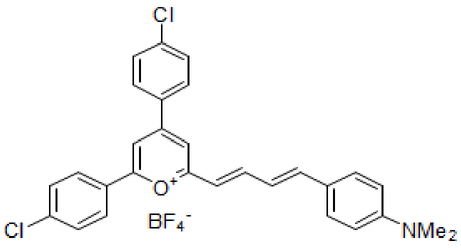 **6jc43-2**	$C_{29}H_{24}BCl_2F_4NO$	560.22	2,4-bis(4-chlorophenyl)-6-((1E,3E)-4-(4-(dimethylamino)phenyl)buta-1,3-dien-1-yl)pyrylium boron tetrafluoride salt	ND	>64
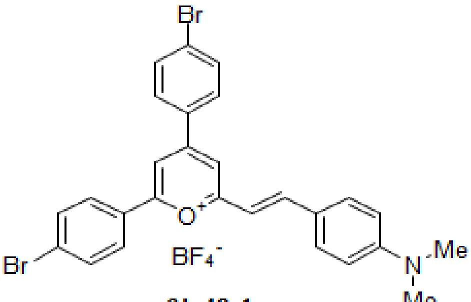 **6jc48-1**	$C_{27}H_{22}BBr_2F_4NO$	623.08	(E)-2,4-bis(4-bromophenyl)-6-(4-(dimethylamino)styryl)pyrylium boron tetrafluoride salt	yes	32
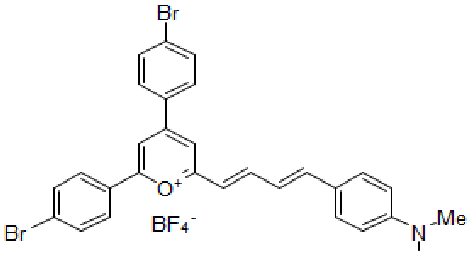 **6jc48-2**	$C_{29}H_{24}BBr_2F_4NO$	649.12	2,4-bis(4-bromophenyl)-6-((1E,3E)-4-(4-(dimethylamino)phenyl)buta-1,3-dien-1-yl)pyrylium boron tetrafluoride salt	yes	>64

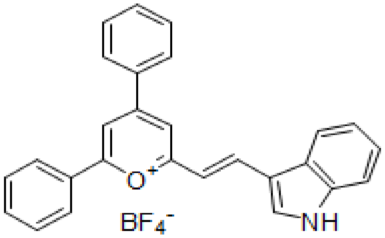 6jc49-1	$C_{27}H_{20}BF_4NO$	461.26	(E)-2-(2-(1H-indol-3-yl)vinyl)-4,6-diphenylpyrylium boron tetrafluoride salt	yes	1
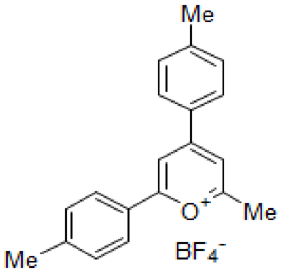 6jc50-2	$C_{20}H_{19}BF_4O$	362.17	2-methyl-4,6-di-p-tolylpyrylium boron tetrafluoride salt	no	>64
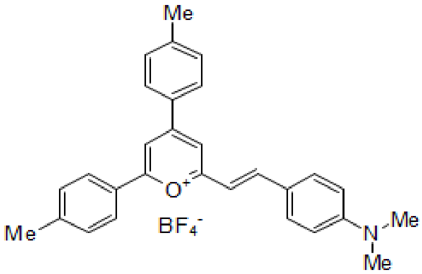 6jc51-1	$C_{29}H_{28}BF_4NO$	493.34	(E)-2-(4-(dimethylamino)styryl)-4,6-di-p-tolylpyrylium boron tetrafluoride salt	yes	0.5

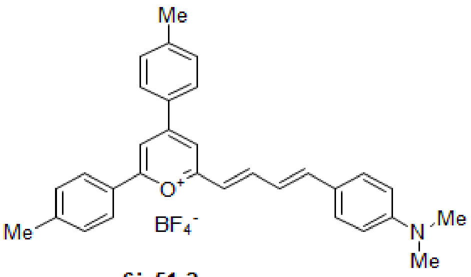 **6jc51-2**	$C_{31}H_{30}BF_4NO$	519.38	2-((1E,3E)-4-(4-(dimethylamino)phenyl)buta-1,3-dien-1-yl)-4,6-di-p-tolylpyrylium boron tetrafluoride salt	yes	2
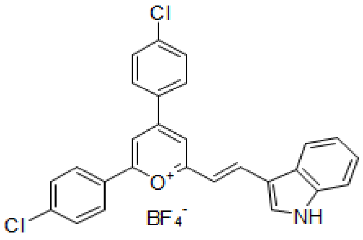 **6jc53-1**	$C_{27}H_{18}BCl_2F_4NO$	530.15	(E)-2-(2-(1H-indol-3-yl)vinyl)-4,6-bis(4-chlorophenyl)pyrylium boron tetrafluoride salt	yes	16
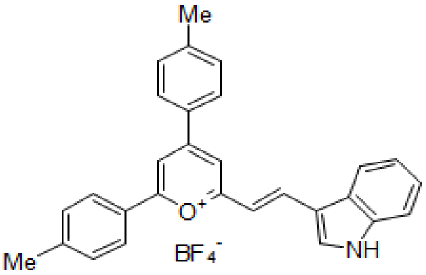 **6jc53-2**	$C_{29}H_{24}BF_4NO$	489.31	(E)-2-(2-(1H-indol-3-yl)vinyl)-4,6-di-p-tolylpyrylium boron tetrafluoride salt	yes	4

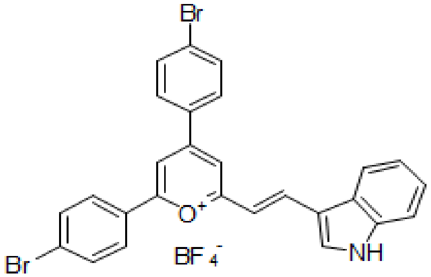 **6jc53-3**	$C_{27}H_{18}BBr_2F_4NO$	619.05	(E)-2-(2-(1H-indol-3-yl)vinyl)-4,6-bis(4-bromophenyl)pyrylium boron tetrafluoride salt	yes	>64
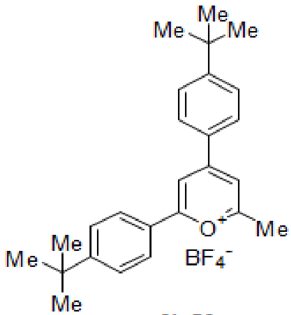 **6jc56**	$C_{26}H_{32}BF_4O$	446.33	2,4-bis(4-(tert-butyl)phenyl)-6-methylpyrylium boron tetrafluoride salt	no	>64
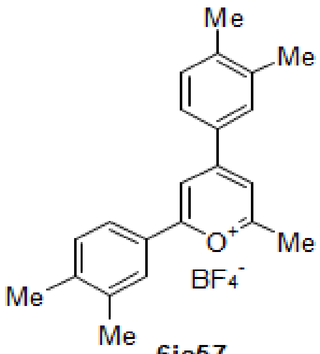 **6jc57**	$C_{22}H_{23}BF_4NO$	390.22	2,4-bis(3,4-dimethylphenyl)-6-methylpyrylium boron tetrafluoride salt	no	>64

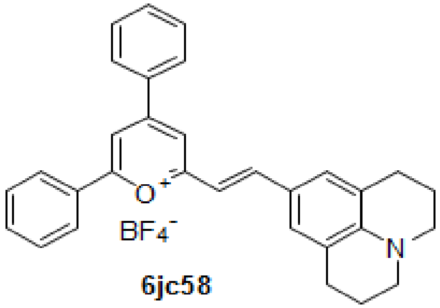 6jc58	$C_{31}H_{28}BF_4NO$	517.36	(E)-2-(2-(1,2,3,5,6,7-hexahydropyrido[3,2,1-ij]quinolin-9-yl)vinyl)-4,6-diphenylpyrylium boron tetrafluoride salt	yes	1
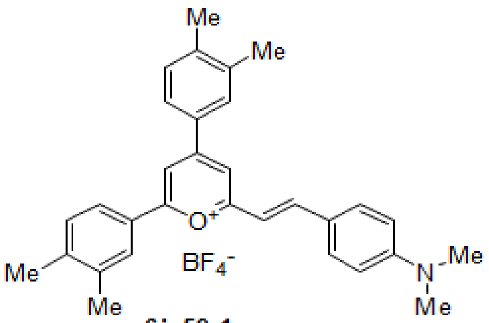 6jc59-1	$C_{31}H_{32}BF_4NO$	521.40	(E)-2-(4-(dimethylamino)styryl)-4,6-bis(3,4-dimethylphenyl)pyrylium boron tetrafluoride salt	yes	2
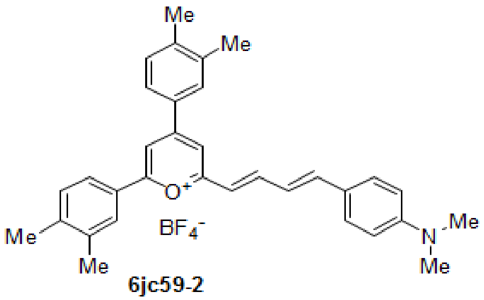 6jc59-2	$C_{31}H_{28}BF_4NO$	547.43	2-((1E,3E)-4-(4-(dimethylamino)phenyl)buta-1,3-dien-1-yl)-4,6-bis(3,4-dimethylphenyl)pyrylium boron tetrafluoride salt	yes	32

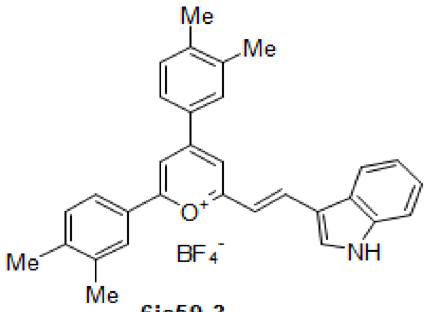<br>6jc59-3	$C_{33}H_{34}BF_4NO$	517.36	(E)-2-(2-(1H-indol-3-yl)vinyl)-4,6-bis(3,4-dimethylphenyl)pyrylium boron tetrafluoride salt	yes	4
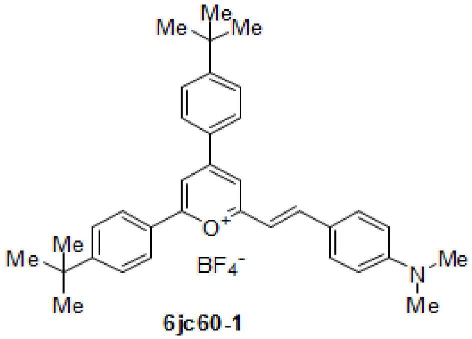<br>6jc60-1	$C_{35}H_{40}BF_4NO$	577.50	(E)-2,4-bis(4-(tert-butyl)phenyl)-6-(4-(dimethylamino)styryl)pyrylium boron tetrafluoride salt	yes	8
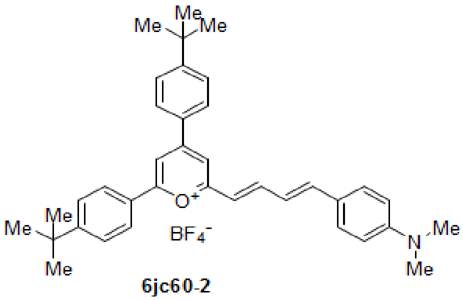<br>6jc60-2	$C_{37}H_{42}BF_4NO$	603.54	2,4-bis(4-(tert-butyl)phenyl)-6-((1E,3E)-4-(4-(dimethylamino)phenyl)buta-1,3-dien-1-yl)pyrylium boron tetrafluoride salt	yes	64

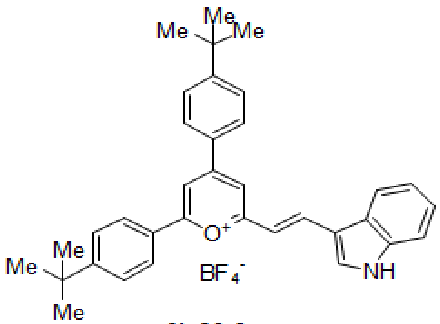<br>**6jc60-3**	$C_{35}H_{36}BF_4NO$	573.47	(E)-2-(2-(1H-indol-3-yl)vinyl)-4,6-bis(4-(tert-butyl)phenyl)pyrylium boron tetrafluoride salt	yes	32
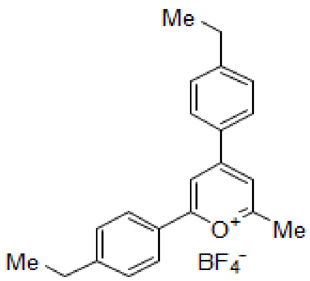<br>**6jc61**	$C_{22}H_{23}BF_4O$	390.22	2,4-bis(4-ethylphenyl)-6-methylpyrylium boron tetrafluoride salt	no	>64
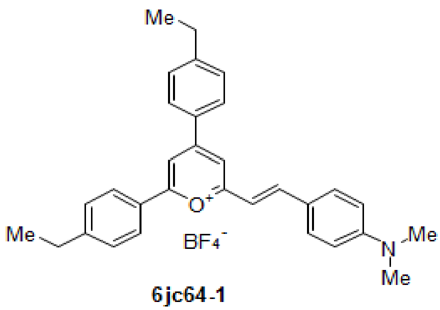<br>**6jc64-1**	$C_{31}H_{32}BF_4NO$	521.40	(E)-2-(4-(dimethylamino)styryl)-4,6-bis(4-ethylphenyl)pyrylium boron tetrafluoride salt	yes	1

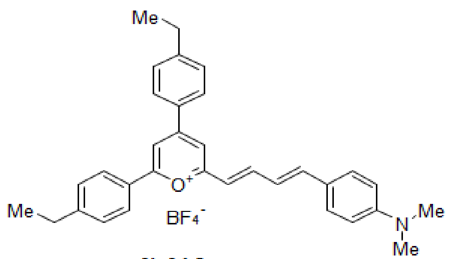 6jc64-2	$C_{33}H_{34}BF_4NO$	547.43	2-((1E,3E)-4-(4-(dimethylamino)phenyl)buta-1,3-dien-1-yl)-4,6-bis(4-ethylphenyl)pyrylium boron tetrafluoride salt	yes	8
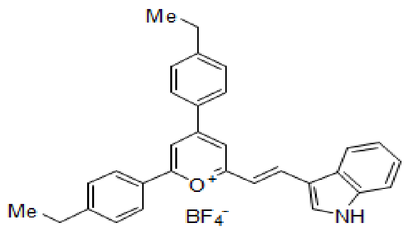 6jc64-3	$C_{31}H_{28}BF_4NO$	517.36	(E)-2-(2-(1H-indol-3-yl)vinyl)-4,6-bis(4-ethylphenyl)pyrylium boron tetrafluoride salt	yes	4
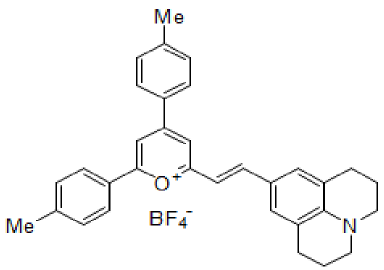 6jc65-1	$C_{33}H_{32}BF_4NO$	545.42	(E)-2-(2-(1,2,3,5,6,7-hexahydropyrido[3,2,1-ij]quinolin-9-yl)vinyl)-4,6-di-p-tolylpyrylium boron tetrafluoride salt	yes	1

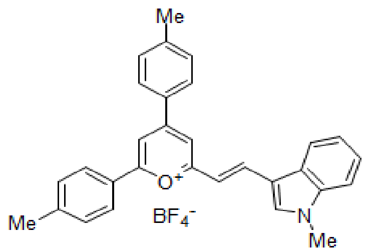 **6jc65-2**	$C_{30}H_{26}BF_4NO$	503.34	(E)-2-(2-(1-methyl-1H-indol-3-yl)vinyl)-4,6-di-p-tolylpyrylium boron tetrafluoride salt	yes	0.5
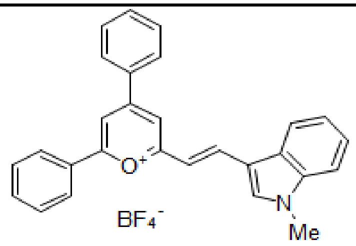 **6jc66-1**	$C_{28}H_{22}BF_4NO$	475.28	(E)-2-(2-(1-methyl-1H-indol-3-yl)vinyl)-4,6-diphenylpyrylium boron tetrafluoride salt	yes	0.5
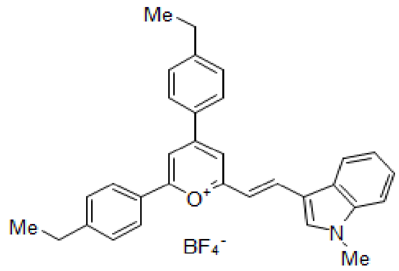 **6jc66-2**	$C_{32}H_{30}BF_4NO$	531.39	(E)-2,4-bis(4-ethylphenyl)-6-(2-(1-methyl-1H-indol-3-yl)vinyl)pyrylium boron tetrafluoride salt	yes	1
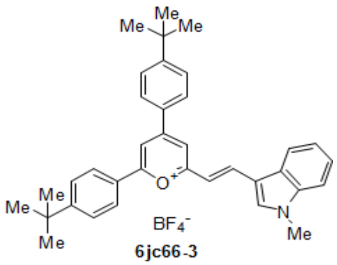 **6jc66-3**	$C_{36}H_{38}BF_4NO$	587.50	(E)-2,4-bis(4-(tert-butyl)phenyl)-6-(2-(1-methyl-1H-indol-3-yl)vinyl)pyrylium boron tetrafluoride salt	yes	16

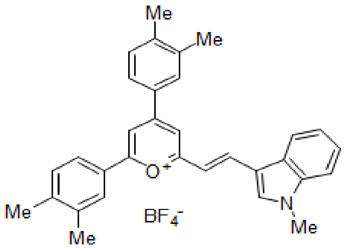 6jc66-4	$C_{32}H_{30}BF_4NO$	531.39	(E)-2,4-bis(3,4-dimethylphenyl)-6-(2-(1-methyl-1H-indol-3-yl)vinyl)pyrylium boron tetrafluoride salt	yes	1
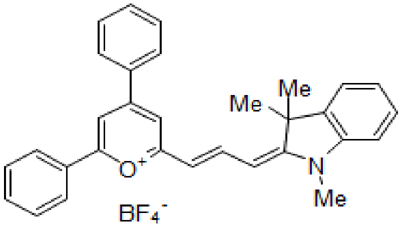 6jc67	$C_{31}H_{28}BF_4NO$	430.57	2,4-diphenyl-6-((1E,3E)-3-(1,3,3-trimethylindolin-2-ylidene)prop-1-en-1-yl)pyrylium boron tetrafluoride salt	yes	0.5
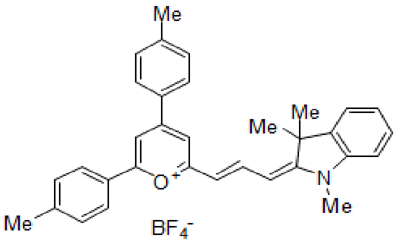 6jc67-A	$C_{33}H_{32}BF_4NO$	448.57	2,4-di-p-tolyl-6-((1E,3E)-3-(1,3,3-trimethylindolin-2-ylidene)prop-1-en-1-yl)pyrylium boron tetrafluoride salt	yes	1
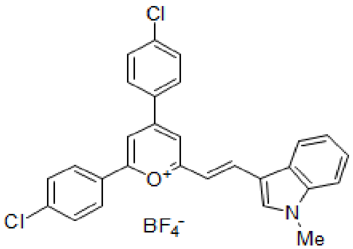 6jc68-1	$C_{28}H_{20}BCl_2F_4NO$	544.18	(E)-2,4-bis(4-chlorophenyl)-6-(2-(1-methyl-1H-indol-3-yl)vinyl)pyrylium boron tetrafluoride salt	ND	64

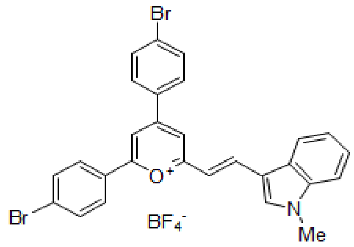 6jc68-2	$C_{28}H_{20}BBr_2F_4NO$	633.08	(E)-2,4-bis(4-bromophenyl)-6-(2-(1-methyl-1H-indol-3-yl)vinyl)pyrylium boron tetrafluoride salt	ND	64
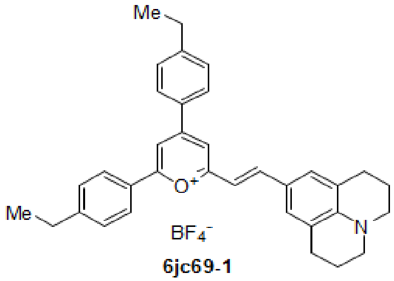 6jc69-1	$C_{35}H_{36}BF_4NO$	573.74	(E)-2,4-bis(4-ethylphenyl)-6-(2-(1,2,3,5,6,7-hexahydropyrido[3,2,1-ij]quinolin-9-yl)vinyl)pyrylium boron tetrafluoride salt	yes	1
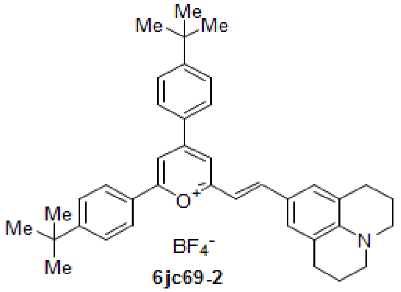 6jc69-2	$C_{39}H_{44}BF_4NO$	629.58	(E)-2,4-bis(4-(tert-butyl)phenyl)-6-(2-(1,2,3,5,6,7-hexahydropyrido[3,2,1-ij]quinolin-9-yl)vinyl)pyrylium boron tetrafluoride salt	yes	32
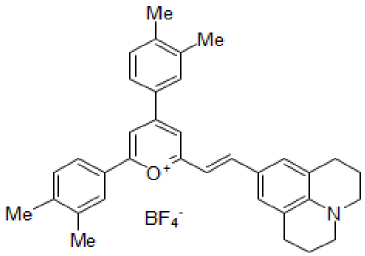 6jc69-3	$C_{37}H_{40}BF_4NO$	573.47	(E)-2,4-bis(3,4-dimethylphenyl)-6-(2-(1,2,3,5,6,7-hexahydropyrido[3,2,1-ij]quinolin-9-yl)vinyl)pyrylium boron tetrafluoride salt	yes	0.5

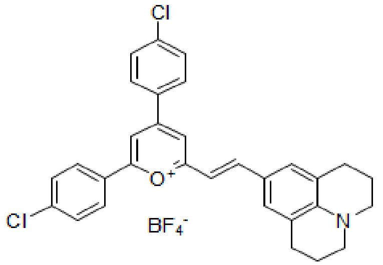 **6jc69-4**	$C_{31}H_{26}BCl_2F_4NO$	586.25	(E)-2,4-bis(4-chlorophenyl)-6-(2-(1,2,3,5,6,7-hexahydropyrido[3,2,1-ij]quinolin-9-yl)vinyl)pyrylium boron tetrafluoride salt	yes	1
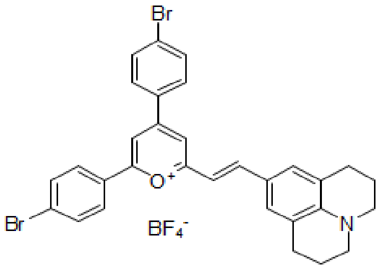 **6jc69-5**	$C_{31}H_{26}BBr_2F_4NO$	675.16	(E)-2,4-bis(4-bromophenyl)-6-(2-(1,2,3,5,6,7-hexahydropyrido[3,2,1-ij]quinolin-9-yl)vinyl)pyrylium boron tetrafluoride salt	yes	4
